# Reconstructing flexible pathways of Aurignacian blade and bladelet production at Vogelherd

**DOI:** 10.1371/journal.pone.0331921

**Published:** 2025-09-16

**Authors:** Benjamin Schürch, Svenja Schray, Nicholas J. Conard

**Affiliations:** 1 Working Group of Early Prehistory and Quaternary Ecology, Department of Geosciences, University of Tübingen, Tübingen, Germany; 2 Senckenberg Centre for Human Evolution and Palaeoenvironment at the University Tübingen, Tübingen, Germany; University of Haifa, Zinman Institute of Archaeology, ISRAEL

## Abstract

The beginning of the Upper Paleolithic represents a key period in human history. At this time, we can grasp the technological concepts that *Homo sapiens* used in the early Upper Paleolithic. The age of the Aurignacian in combination with the three-dimensional ivory artworks, musical instruments and personal ornaments in the Swabian Jura sites emphasize the importance of this region for understanding and defining the Upper Paleolithic. During that time blade and bladelet production became the central interest of lithic production. The study of these lithic reduction sequences is essential for understanding technological inventions and socio-economic behaviors of early anatomically modern humans in Central Europe. So far, however, the lithic technology from the Aurignacian of the Swabian Jura has only been studied in detail at the site of Geißenklösterle. In this paper, we provide an exhaustive study based on the rich lithic assemblage from Vogelherd Cave combining both the *chaîne opératoire* approach and attribute analysis. This work highlights the importance of carefully sorting minimal raw material units and engaging in systematic refitting. These observations allow us to reconstruct entire reduction sequences including the biographies of both cores and tools. The source and physical characteristics of lithic raw materials greatly influenced decision-making during the reduction process. As in many other Paleolithic contexts, Aurignacian knappers thoroughly exploited imported raw materials while exhausting low quality local material to a lesser degree. Comparisons with other assemblages from the region help to facilitate the characterization of the Swabian Aurignacian. This comparison allows us to separate regional adaptations from more site-specific behaviors.

## Introduction

Understanding lithic reduction sequences is pivotal to reconstruct human lifeways in the Paleolithic. The onset of the Upper Paleolithic in Central Europe marks a shift from a Middle Paleolithic flake based industry, to blade and bladelet industries. For the southwest (SW) German Middle Paleolithic, it has become increasingly evident in recent decades that other reduction concepts such as Quina, Discoid, core-on-flakes and blade production also play an important role [1–8]. Preceded by an occupational hiatus [9,10], the Aurignacian marks the beginning of the Upper Paleolithic in southern Germany with a lithic production that is dominated by laminar production [11–14].

Core typology is often used to provide information about the organization of lithic assemblages. However, to get a more complete picture of the *chaîne opératoire*, more than just core typology is needed. For SW Germany, J. Hahn could impressively show this with his analysis of the lithic assemblage of Geißenklösterle [12]. He used refits and minimal raw material units to gain unique insights into core reduction. Here, we present similar analyses on the lithic assemblage of the Aurignacian of Vogelherd with an updated methodology. The artifacts we analyzed are from the Aurignacian layers IV and V and date to between 41,000 and 35,000 cal BP [9,10,15]. In addition to analyzing the cores themselves, we conducted a refitting study after sorting parts of the Vogelherd assemblages into minimal raw material units [e.g., 16]. This enables us to reconstruct the *chaîne opératoire* in the Aurignacian of Vogelherd and allows to compare the lithic production at Geißenklösterle and Vogelherd. Since there is no fine chronological sequence at Vogelherd due to the early excavation and the methodology involved [17–22], the comparison with the Geißenklösterle Aurignacian makes it possible to highlight characteristics and show possible temporal trends of the Swabian Aurignacian.

One important technological innovation in the Upper Paleolithic is the production of microlithic artifacts (bladelets) on volumetric cores and carinated pieces [23–27]. The role of carinated pieces, which was investigated by use-wear analysis, is discussed in detail in a separate article. We will refer to these results repeatedly, as they also have implications for the lithic production system. One of the central questions is how the lithic reduction is organized and how we can explain this organization in a socio-economic context.

### Overview on the Swabian Aurignacian lithic reduction processes

In 1988, Hahn published a monograph focusing on the Aurignacian layers of Geißenklösterle from the first half of his excavations [12]. He presented a detailed study of core reduction by studying 38 cores and core fragments, raw material units and refitted lithic sequences. This work is still of central importance today and has been updated by new analyses [28,29]. Further studies of the lithic assemblages of other sites like Hohle Fels [13,30,31] or Hohlenstein-Stadel [32] are relevant and add to the findings published by Hahn in 1988.

The production process at Geißenklösterle is described the following [11,12]: nodules are selected and tested before the transport to the cave. A striking platform is created. Production is initiated along a natural or prepared crest. The direction of production is adapted to the volume of the matrix. A soft hammer is used. Dorsal reduction often precedes blade reduction. After the convexity of the matrix is not usable anymore, the core is re-configured. Depending on the volume, this takes place in the form of preparation of the striking surface by flake removals or core tablets as well as preparation of the core foot, flanks or crest. These crests are often only prepared on one side and can be referred to as *neo-crête*. The small size of the nodules dictates minimized preparation to reduce the loss of volume. Following the preparation, a new phase of blade production takes place. The cycle of preparation and production can be repeated until the striking surface and/or production surface cannot be restored. The core can be rotated to start production along a new surface, or it will be discarded. This production concept resulted into three schemes of production after Hahn: 1.) Core with one production and one striking surface. 2.) Core with two striking and one production surface. 3.) Core with two striking and two production surfaces in a 90 degree towards each other. Hahn did not distinguish between blade and bladelet production.

Teyssandier presented a further technological analysis of the Geißenklösterle Aurignacian as part of his dissertation [28,29]. He describes the blade production as follows: preparation is kept to a minimum. Natural surfaces of the matrix are exploited making the preparation of an initial crest redundant. The direction of the production is *semi*- or *demi-tournant* [after 23] to maintain convexity together with the detachment of elongated cortical flakes and crests. The former is more frequent than the latter. Crests are often only prepared one sided and located on the lateral face of the core. The angle of the striking platform is around 80°. Before each production the platform is prepared by detaching small flakes or sometimes core tablets. Direct soft organic hammer is used. The preparation of striking accidents can result in bipolar removals, but the production is usually unipolar.

Additionally, Teyssandier identifies different types of bladelet production. The most common production concepts are prismatic cores (similar to blade production) and carinated pieces (including carinated and nosed end-scrapers; after [23,26]). Less frequent is the bladelet production on carenoid burins, Kostienki pieces [12,33,34] and bladelet production that is interposed within blade production.

One of the central conclusions from Hahn on core reduction is the following:

“*Der Kernabbau scheint vor allem durch die geringe Knollengröße gesteuert zu sein, was zu einer opportunistischen Zerlegungsstrategie führte*” [12,35], [Core reduction appears to be primarily influenced by the small nodule size, which led to an opportunistic reduction strategy].

Whether opportunistic reduction is also evident in Vogelherd or can be confirmed by our analyses at Geißenklösterle is central to our understanding of the *chaîne opératoire* and lithic reduction systems.

## Materials

### Vogelherd

**Fig 1:**
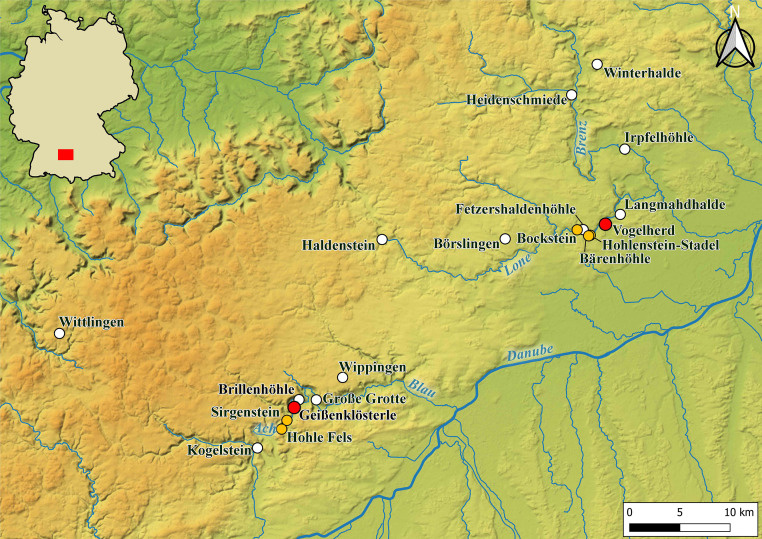
Map of SW Germany with selected Paleolithic sites. Central sites of the study in red, sites mentioned in the text in orange color. Background map: (https://doi.org/10.5281/zenodo.3460301; © ROCEEH/ University of Tübingen; [36]).

**Fig 2:**
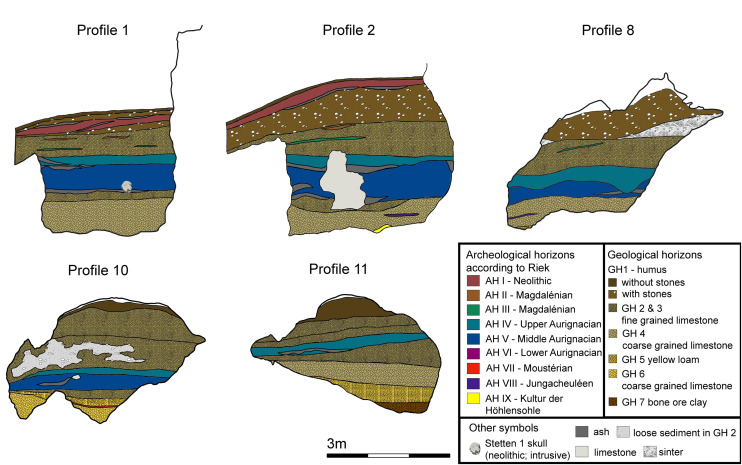
Vogelherd. Profiles (top) and plan (bottom) of the Aurignacian horizons IV and V depicted in light blue (AH IV) and darker blue (AH V) (profiles and plan modified after Riek 1934).

Vogelherd was almost completely excavated in 1931 by Gustav Riek and the University Tübingen [21]. The site is located in the Lone Valley in SW Germany (Fig 1). Archeological horizons (AH) IV and V of Vogelherd are attributed to the Aurignacian [9,10,14,15,21,37]. Horizons I-III contain Neolithic, Magdalenian and Gravettian finds. Horizons VI-IX can be attributed to the Middle Paleolithic. The Aurignacian layers date to between 41,000 and 35,000 cal BP [10,15,18]. For this study, the assemblages of layer IV, IV/V and V (n = 5710) were analyzed together, because we could find several refits that connect both layers (38 connections). The layers are evenly distributed across the cave and are present in all profiles (Fig 2). The excavation revealed outstanding artifacts from the Aurignacian period. The number of ivory figurines [14,21,38], lithic, bone and ivory artifacts [14,39] is high for the early Upper Paleolithic. During Riek’s excavation the sediments from the cave were dumped outside the cave on the slope between the southwestern and southern entrances of the cave.

The reassessment of the site has revealed some mixing between the horizons [18]. A low number of Middle Paleolithic tools and cores are mixed into the Aurignacian layers and few tool types indicate mixing of younger material into the Aurignacian layers. Previous studies suggested that the few backed elements at Vogelherd point towards a local transition from the Aurignacian to the Gravettian [40]. The techno-typological study of AH IV and V showed that only 2,29% of the tools of the site (n = 58 of 2535 tools) could possibly be attributed to other technocomplexes. The majority of lithic and organic artifacts as well as the radiocarbon dates from the Aurignacian horizons can be attributed to the Aurignacian occupation [10,15,39,41–44].

Riek’s backdirt was excavated by N. J. Conard from the University of Tübingen between 2005 and 2012 and again in 2022 and 2023 [45–50]. The excavations were carried out until geologic bedrock, or until the old topsoil were reached. Within the backdirt, different sediments could be detected. On top of the stratigraphy of the backdirt, a topsoil layer of about 10 cm thickness (HU) could be observed [51,52]. Below HU is the topsoil layer of light brown sediment interbedded with a lot of calcareous debris (HL/KS). This layer made up the main part of the sediment of the backdirt [51]. Under HL/KS there was a dark brown, loamy sediment of about 20 cm thickness with a high proportion of calcareous debris (DKS). Even though they lack their original context, a large part of the finds from these sediments can be attributed to the Aurignacian. The study of the finds from the backdirt is still ongoing. For this study, artifacts from Conard’s excavation of the backdirt were only included when they were part of refits or minimal raw material units and in rare cases if they were of specific importance for the understanding of the technology of the site. These artifacts were not included in the tables, which only show the assemblage of Rieks’s excavation (IV and V).

Additionally, artifacts from the collections of the Naturfreunde Heidenheim (1931 excavation), the collection Seeberger (backdirt) and the collection Huber (backdirt) were analyzed for this study. The studied stone artifacts from Vogelherd are stored in the collection of the University of Tübingen. Parts of the assemblage are displayed at the Urgeschichtliches Museum in Blaubeuren and in the Museum of the University of Tübingen. Previous analyses of the material from Vogelherd of layers IV and V can provide an overview on the lithic assemblage of the site [9,14,15,19,21,40,49,53–71]. However, with the exception of a preliminary study [72] detailed analyses of the core technology and the reduction concepts are absent. The number of artifacts studied from the four major previous analyses are presented in Table 1. The information on the size of the assemblage varies between the different researchers. Other analyses focused on specific tool types are not listed here [e.g., 61,73]. No permits were required for the described study, which complied with all relevant regulations.

**Table 1:** Vogelherd. Numbers of lithic artifacts and tools from the different analysts of Vogelherd.

Analyst	Assemblage	IV	IV/V	V	Total n
**Riek 1934**	complete assemblage	3555	not det.	2317	5872
	tools	1731	not det.	731	2462
**Sonneville-Bordes 1971**	complete assemblage	not det.	not det.	not det.	not det.
	tools	1223	not det.	685	1908
**Hahn 1977**	complete assemblage	3387	not det.	2133	5520
	tools	1729	not det.	910	2639
**Chang (Herkert et al. 2015)**	complete assemblage	3031	not det.	1920	4951
	tools	not det.	not det.	not det.	not det.
**This study**	complete assemblage	3398	105	2207	5710
	tools	1675	47	812	2535

### Geißenklösterle

The Paleolithic deposits of Geißenklösterle Cave were discovered in 1958 [74]. After E. Wagner conducted excavations to evaluate the state of the deposits in 1973, J. Hahn joined him in 1974 leading the excavations until 1991 [12,74]. Between 2000 and 2002 a team under the direction of N. J. Conard from the University of Tübingen returned to the site focusing on the transition from the Upper to the Middle Paleolithic layers of the site [74,75]. The Geißenklösterle deposits comprise a stratigraphy from the Mesolithic to the Middle Paleolithic. Seven Aurignacian archaeological horizons (AH) were defined in six geological horizons (GH). Hahn groups these layers into two main occupation horizons AH II (IIn, IIa, IIb) and III (IId, III, IIIa, IIIb) that were split by taphonomic processes [12]. The Aurignacian layers are dated to between 42,500 and 35,000 cal BP [76], which is contemporaneous to other sites in the Swabian Jura [9,10,13,77].

## Methods

For the analysis we used the *chaîne opératoire* approach [78,79] as well 50 discrete and metric attributes (Table 2) [12,80]. 14 of these attributes were applied only to the core category. The dimensions were taken from the pieces according to Hahn [34]. We used the 12 mm threshold to separate blades and bladelets [81]. All attributes were entered into a Microsoft Access Database. Statistical analysis was conducted in Excel, SPSS and JMP.

**Table 2:** Analyzed attributes of the Vogelherd assemblage (p/a = presence/absence; m = measurement; d = description, n = number of the attribute).

General attributes	Blank attributes	Tool	Core
Origin of excavation or collection	Platform type (d)	Tool type (d)	Type of blank produced (d)
Technological description (d, e.g., core, tool, ...)	Striking remnant/platform modification (d)	Modification 1 (d)	Core type (d)
Length in mm (m)	Width of striking remnant/platform in mm (m)	Position of Mod. 1 (d)	Number of removal surfaces (n)
Width in mm (m)	Thickness of striking remnant/platform in mm (m)	Direction Mod. 1 (d)	Direction of production (d)
Thickness in mm (m)	Blank profile (d)	Length of Mod. 1 in mm (m)	Length of production surface in mm (m)
Weight in g (m)	Pronunciation of bulb (d)	Height Mod. 1 in mm (m)	Width of production surface in mm (m)
Preservation (p/a)	Bulbar scar (p/a)	Depth Mod. 1 in mm (m)	Thickness of production surface in mm (m)
Heat impact (p/a)	Lip (p/a)	The same procedure was applied for further modifications	Exterior platform angle (m)
Frost impact (p/a)	Hertzian cone fracture (p/a)		Preparation of the platform (d)
Patination (p/a)	Dorsal reduction (d)		Number of end-products/negatives visible (n)
Percentage of cortex (m, e.g., 0%, 1–25%, 26–50%, ...)	Form of distal end (d)		Width negative of end- product 1 (2, 3) in mm (m)
Raw material (d)	Exterior platform angle (m)		Length negative of end-product 1 (2, 3) in mm (m)
Raw material variety (d)	Percussion technique (based on the previously listed characteristics) (d)		Secondary use as hammerstone (p/a)
Refit with (artefact ID)			Directionality of the negatives on production surface (d)
Chaîne opératoire (d; see Table 4)			

Cores (n = 400) were classified using a combination of different attributes that were used for the analysis of other Aurignacian sites [12,23,25,29,82,83]. The cores were mainly assigned to the following types: narrow-sided core, semi-circumferential core, wide-faced flat core, multi-platform core; carinated piece, prepared core, tested core, truncated-faceted core (or Kostienki-end) and combinations of the types (see results for illustrations of the different core types).

### Narrow-sided core

These cores were reduced on a narrow face, where a single negative can also pitch over onto the wide face of the core. Maintenance of these cores and the convexity are kept through preparing the wide face of the core and by removing core tablets. Narrow blades and bladelets with mainly a triangular cross section are produced from these cores. Both nodules and larger flakes are used as starting volume. Some of these cores produced on flakes have similarities with large burins, especially burins on truncation.

### Semi-circumferential core

These cores display a reduction of the narrow and the wide face of the core. They can be referred to as *semi-tournant* [23]. The production of these cores is based on the convexities of the narrow side and the transitional part between the narrow and wide face. Most of the blades are not produced on the wide face of the core. Target products are produced on the narrow side and have the same characteristics as the ones from narrow-sided cores. In addition, target products were produced from the transitional part of the core; they can be curved and asymmetrical.

The transition of the reduction surfaces is visible on the dorsal face of the blanks when looking at the width of the previous negatives. Blades struck from the wide face of the core were often postulated as the aim of the Aurignacian blade production [67,84]. They are wider and sometimes with a trapezoidal cross-section; besides they are often used for large end-scrapers, carinated pieces respectively.

### Wide-faced flat core

These cores can be characterized as exhausted semi-circumferential cores. During the reduction of a semi-circumferential core, it sometimes is highly reduced in the transitional part so that the convexities on the lateral part and the transitional part are completely exhausted, like observed with cores from Fumane Cave [7,25]. Examples of the reduction of a semi-circumferential core into a wide-faced flat core is known from Geißenklösterle Cave with refit A1 [11,12] core A1, plate 3.3−4, [85]. The reduction of these exhausted cores does not necessarily correspond to the *débitage frontal* [86]. *Débitage frontal* will only occur on cores with a natural convexity and pebbles with a long and narrow shape. With such a natural convexity of the core, it would be reduced like a narrow-sided core, where the exhausted cores look like a wide-faced flat core. Examples for refitting of cores reduced that way were found in Geißenklösterle [12 core A9, plate 6] and Abri Pataud [87, Fig. VI-1, p. 779].

### Multi-platform core

These are cores that were rotated after an initial reduction sequence to use a new reduction face thereafter. These cores have at least two reduction surfaces. The new reduction face is mostly created on a ridge that was produced by the previous reduction.

### Prepared core (Vollkerne)

The term prepared core describes cores that were completely configured for production, but the reduction never started or was unsuccessful from the beginning, e.g., the first production attempt ended in a hinge.

### Carinated piece

Under this term we subsume all sub-types of carinated end-scrapers, nosed end-scrapers and carinated burins. These pieces are bladelet cores with a unidirectional semi-circumferential production pattern [27,88–91]. Here, Carinated and nosed end-scrapers are defined by a thickness of the scraper end of over 12 mm. This number was determined by the result of a separate paper, were we analyzed carinated artifacts and end-scrapers with use-wear analysis [82]. If the scraper end is thicker than 12 mm the artifact is defined as a core and not a tool. All carinated burins are considered cores independent of their thickness. For a more detailed description see the referenced paper [82].

### Truncated-faceted core (Kostienki end)

These pieces are defined by at least one truncated end that is placed on the ventral face of a blank. The truncation is used as a platform for the reduction of straight and thin bladelets on the dorsal face. In some cases, a ridge created by the truncation on the ventral face is used for the production of bladelets as well. This is similar to carinated burins. The bladelets produced can be curved, twisted or in some cases straight. It is therefore possible to produce two morphological different types of bladelets from these cores. Hahn used the term Kostienki knife [33] to refer to this artifact type at Geißenklösterle [12,34,65]. The artifacts described here are not knifes but cores. We therefore refer to them as truncated-faceted cores [92] or Kostienki ends [93]. For a detailed discussion of the term Kostienki, we refer to overviews by Frick [93] and Klaric [83]. For some of these cores, bladelets are not only reduced on the dorsal face but also on the truncation of the cores. Examples of bladelets from this core type are present (e.g., Fig 24, 1) as well but not abundant due to the size selection in Riek’s excavation. So far, we have not been able to find any exact parallels to this reduction concept where a bladelet production is also conducted on the truncation itself in combination with the bladelet production on the dorsal side of the blank [92,94]. However, there are similarities with the *Paviland burin* [89,95] and the *pièces de la Bertonne* [96–98].

Other core categories identified at Vogelherd represent either Middle Paleolithic core types (Levallois or Quina) mixed into the Aurignacian assemblage, or cores that cannot be classified into one of the forementioned categories (single platform, ventral core, dorsal core, exhausted core, simple flake core). It should be noted that the core categories chosen here should not be equated with one reduction concept each, since they can reflect different stages of reduction [see results and 99] and are mostly the result of an asymmetrical core reduction [100,101].

In addition to the typological and technological data recorded [34,102], we carried out working stage analyses for a selection of cores [11–13,25,103,104]. This analysis is complemented by refits and by the results of the sorting of raw material units and minimal raw material units [12,16,105]. Minimal raw material units are defined as all artifacts that belong to a unit most likely originating from one nodule. Since there is no way to prove this with absolute certainty for each individual artifact, Jurassic chert variants with a very characteristic pattern, coloration, cortex and specific inclusions were selected for the minimal raw material units presented here. Furthermore, many refits within these units show that there is a high relatedness inside these units.

## Results

### Aurignacian lithic assemblage of Vogelherd

The Aurignacian lithic artifacts from Vogelherd are distributed between layers IV (n = 3398), V (n = 2207) and artifacts labeled IV/V (n = 105). Jurassic chert is by far the most common raw material in the assemblage with three different variants of Jurassic chert, the gray or beige variant of Jurassic chert, the brown Jurassic chert (Bohnerzhornstein) and Siliceous limestone (Kieselkalk). These raw materials make up 85,69% of all raw materials (Table 3). We also identified tabular Jurassic chert from Bavaria (n = 185; 3,22% of the assemblage), the origin of a sample of these were confirmed using IR-analysis [106]. Besides these sub-types of Jurassic chert, the second most used raw material is limestone. This is very unusual, not only for the Aurignacian, but also for the Paleolithic in SW Germany. Keuper chert, from the Upper Triassic, is present as well. Radiolarite only plays a minor role in the assemblage, which is typical for the Aurignacian, whereas it is more common in the Middle Paleolithic, the Gravettian and the Magdalenian in the region [107,108]. Tertiary chert is present with a low percentage. The macroscopic determination of six artifacts was validated in a separate study [106]. Muschelkalk chert (Middle Triassic chert), probably originating from the Neckar gravels is present in small amounts [107].

**Table 3:** Vogelherd. Raw materials of all lithic artifacts of AH IV-V.

Raw Material	IV	IV/V	V	Total n	%
**Jurassic chert**	1540	41	1131	2712	47,50%
**Brown Jurassic chert**	1403	35	726	2164	37,90%
**Limestone**	125	10	84	219	3,84%
**Keuper chert (Lower Triassic chert)**	67	2	56	125	2,19%
**River gravel**	57		58	115	2,01%
**Radiolarite**	55	6	44	105	1,84%
**Quartzite**	51	3	42	96	1,68%
**Sandstone**	25	3	13	41	0,72%
**Siliceous limestone**	14	3	15	32	0,56%
**Not determined/unknown**	19	1	9	29	0,51%
**Quartz**	12		15	27	0,47%
**Tertiary chert**	14		7	21	0,37%
**Lacustrine chert**	10	1	2	13	0,23%
**Muschelkalk chert (Middle Triassic chert)**	6		5	11	0,19%
**Total**	**3398**	**105**	**2207**	**5710**	**100%**

We classified the different phases of the *chaîne opératoire* approach (Table 4) to show the state of reduction of the assemblage. It is apparent that phase 0 is frequently represented. These are natural debris or unworked river gravels. Decortification and core configuration (phase 1) is only represented with 11.4%. Target blanks (phase II) are mostly blades (n = 723), bladelets (n = 128) or flakes (n = 73). The latter are mainly elongated or blade like and were also sorted into this phase. None of them are formal tools. A total of 355 pieces are classified to phase IV, discard of cores. However, 400 cores are listed below. This can be explained by the fact that 45 cores were also used as tools. In phase V, 2535 formal tools are listed, these make up 44,41% of the assemblage. This is a very high number and is probably caused by the selection process in 1931. In general, the selection process of the 1931 excavation led to an underrepresentation of small and unmodified artifacts, especially bladelets.

**Table 4:** Vogelherd. Phase of *Chaîne opératoire* of all lithic artifacts of AH IV-V (Phase 0 = debris and unmodified river gravels; Phase I = Configuration flakes with higher cortex coverage; Phase II = mainly unmodified blades and bladelets; Phase III = Configuration flakes with lower cortex coverage; Phase IV = Cores; Phase V = Formal tools).

*Chaîne opératoire*	IV	IV/V	V	Total n	%
**Phase 0; Raw material procurement**	206	12	186	404	7,08%
**Phase I; Decortification, Core configuration**	370	10	271	651	11,40%
**Phase II; Removal of target blanks**	542	16	366	924	16,18%
**Phase III; Core reconfiguration**	370	10	461	841	14,73%
**Phase IV; Discard of cores**	233	10	111	355	6,22%
**Phase V; Use**	1677	47	812	2535	44,40%
**Total**	**3398**	**105**	**2207**	**5710**	**100%**

On more than half of the assemblage cortex is not present (56,34%) (Table 5). Cortex coverage of 100% is present in 190 of the cases (3,33%), however, 86 of them stem from unmodified river pebbles. This leaves us with 104 decortification flakes with 100% percent of cortex coverage and 129 pieces with a coverage of between 75 and 99%.

**Table 5:** Vogelherd. Cortex coverage of all lithic artifacts of AH IV-V.

Cortex in %	IV	IV/V	V	Total n	%
**n.d./unclear**	13	2	9	24	0,42%
**0**	1966	52	1199	3217	56,34%
**0-24**	707	17	404	1128	19,75%
**25-49**	344	18	321	683	11,96%
**50-74**	183	5	151	339	5,94%
**75-99**	79	3	47	129	2,26%
**100**	106	8	76	190	3,33%
**Total**	**3398**	**105**	**2207**	**5710**	**100%**

Only 36,73% of the assemblage is completely preserved (Table 6). Often (61,03%) only one part of the artifact (distal, medial, proximal) is preserved, whereas pieces that are broken along the central axis (1,34%) are less frequent. The high degree of fragmentation is, however, mainly not caused by frost. Frost damage could only be detected on 0,12% of the assemblage and influence or damage by heat or fire on 2,42%. The use-wear study we conducted on the end-scrapers of the assemblage showed varied preservation [82], however, the surfaces are mostly preserved well. The high degree of fragmentation is therefore best explained by use and resulting fragmentation and possibly trampling.

**Table 6:** Vogelherd. Preservation of all lithic artifacts of AH IV-V.

Preservation	IV	IV/V	V	Total n	%
**Complete**	1183	42	872	2097	36,73%
**Distal part preserved**	777	21	375	1173	20,54%
**Medial part preserved**	640	8	377	1025	17,95%
**Proximal part preserved**	426	14	296	736	12,89%
**Broken multiple times**	243	16	217	476	8,34%
**Longitudinal fracture**	38	2	35	75	1,31%
**Unclear**	91	2	35	128	2,24%
**Total**	**3398**	**105**	**2207**	**5710**	**100%**

The tool types present at Vogelherd are variable (Table 7, for detailed list see supplementary file 1). For our study, we included also pebble tools to give a full overview of all modified pieces. These are especially useful for reconstructing percussion techniques. Besides the tools that are common all over the Upper-Paleolithic (end-scrapers and burins in general) [33,34], there are diverse tools that are characteristic for the Aurignacian, like nosed end-scrapers, Dufour bladelets, one Font-Yves bladelet, pointed blades, strangulated blades and a large variety of tools that combine two tool ends. Few tools do not fit into the Aurignacian and were assigned either to Middle Paleolithic tool types or middle Upper Paleolithic tool types.

**Table 7:** Vogelherd. Simplified overview of the tool types of AH IV-V. Detailed overview of tool types can be found in the supplementary (S1 Table).

Tools simplified overview	IV	IV/V	V	Total n	%
**Pebble tools**	70	4	43	117	4,62%
**End-scrapers and truncated pieces**	361	9	166	536	21,14%
**Burins**	181	8	102	291	11,48%
**Laterally retouched tools**	376	13	207	596	23,51%
**Pointed tools**	191	3	50	244	9,63%
**Splintered piece**	160	2	83	245	9,66%
**Middle Paleolithic tool types**	25	3	15	43	1,70%
**Middle/Late Upper Paleolithic tool types**	15	0	0	15	0,59%
**Combination tool/tool**	274	5	134	413	16,29%
**Combination tool/core**	22	0	13	35	1,38%
**Total**	**1675**	**47**	**813**	**2535**	**100%**

### Vogelherd core technology

#### Core assemblage.

In total there are 400 cores in the Aurignacian layers of Vogelherd. 123 from V, 267 from IV and 10 from IV/V. 163 of the cores can be attributed as carinated pieces (n for the layers: IV = 123; V = 34; IV/V = 6). Jurassic chert (n = 244) and brown Jurassic chert (n = 99) together make up 85,75% of the core assemblage and are by far the most used raw materials (Table 8). This is comparable to the numbers of these materials in the whole assemblage with 85,4%. Other raw materials like limestone, radiolarite or quartzite rather play a minor role in the assemblage. However, it is notable that limestone is the third most used raw material.

**Table 8:** Vogelherd. Raw material of cores of AH IV-V.

Raw material of cores	IV	IV/V	V	Total n	%
**Jurassic chert**	161	4	79	244	61,00%
**Brown Jurassic chert**	66	5	28	99	24,75%
**Limestone**	14		4	18	4,50%
**Radiolarite**	10		4	14	3,50%
**Quartzite**	4	1	6	11	2,75%
**Keuper chert (Lower Triassic chert)**	6		2	8	2,00%
**Tertiary chert**	3			2	0,75%
**Not determined/unknown**	1			1	0,25%
**River gravel**	1			1	0,25%
**Siliceous limestone**	1			1	0,25%
**Total**	**267**	**10**	**123**	**400**	**100%**

Only 215 cores were reduced directly on nodules, slabs, or debris (Table 9). The rest were produced on flakes, blades or bladelets. The use of blanks for production is noticeably not limited to the carinated pieces and truncated-faceted cores (n = 157) but also other cores were produced on blanks (n = 28).

**Table 9:** Vogelherd. Matrices used for cores of AH IV-V.

Matrix	IV	IV/V	V	Total n	100,00%
**Nodule/slab**	124	4	76	204	51,00%
**Flake**	85	4	22	111	27,75%
**Blade**	53		20	73	18,25%
**Angular debris**	4	2	5	11	2,75%
**Bladelet**	1			1	0,25%
**Total**	**267**	**10**	**123**	**400**	**100%**

We subsumed the cores under the following categories that were defined in more detail above: regular volumetric reduction (semi-circumferential, narrow-sided, wide-faced flat and multi-platform), carinated pieces, truncated-faceted cores (Kostienki-end), a variety of cores tested or only minorly reduced (tested core, simple flake core, single platform, ventral core and dorsal core), some intrusive Middle Paleolithic core types (Levallois, discoid, Quina) and combinations of different core types (Table 10). Carinated cores are the most common core type, followed by semi-circumferential and narrow-sided cores. Truncated-faceted, multi-platform and wide-faced flat cores are common in both layers as well. Combinations of different core types can occur. They can be identified in Table 10 by the slash separating the different types.

**Table 10:** Vogelherd. Core types of AH IV-V (* These cores can be a combination of tool and core, 35 of the 400).

Core types	IV	IV/V	V	Total	%
Carinated*	100	5	28	133	33,25%
**Semi-circumferential**	25	1	18	44	11,00%
**Narrow-sided**	14	1	15	30	7,50%
**Unknown-broken**	18		12	30	7,50%
**Core blank = prepared core**	22	1	6	29	7,25%
Truncated-faceted*	19		6	25	6,25%
**Multi-platform**	12		10	21	5,25%
**Carinated/carinated**	14		5	20	5,00%
**Tested core**	11		7	18	4,50%
**Wide-faced flat**	6		4	10	2,50%
**Carinated/truncated-faceted**	6		2	8	2,00%
**Simple flake core**	3		2	5	1,25%
**Ventral core**	5			5	1,25%
**Single platform**	2		1	3	0,75%
**Levallois**	1		2	3	0,75%
**Wide-faced flat/burin like**	2			2	0,50%
**Discoid**	1		1	2	0,50%
**Quina**		1	1	2	0,50%
**Exhausted core**	2			2	0,50%
**Burin like**	2			2	0,50%
**Narrow-sided/carinated**		1	1	2	0,50%
**Carinated/truncated-fac./carinated**	1			1	0,25%
**Narrow-sided/burin like**			1	1	0,25%
**Semi-circumferential/burin like**	1			1	0,25%
**Dorsal core**			1	1	0,25%
**Total**	**267**	**10**	**123**	**400**	**100%**

#### Refits and minimal raw material units.

From Vogelherd, including all excavations, collections and archeological horizons, we have 141 lithic refits with 334 artifacts included. 49 of the refits are broken artifacts and 92 are refitted sequences [109]. 123 refits have at least one artifact from layer IV, IV/V or V. These refits help to reconstruct the configuration and reduction processes. In this section we are combining refits with data from minimal raw material units. We selected three minimal raw material units of Jurassic chert (JH1d, JH1w, JH1m) for a detailed presentation. For all three units one core, primary or secondary core preparation, blades, bladelets and tools are present. For two units (JH1d, JH1w) we also analyzed use-wear traces for the end-scrapers and carinated end-scrapers [82].

**Fig 3:**
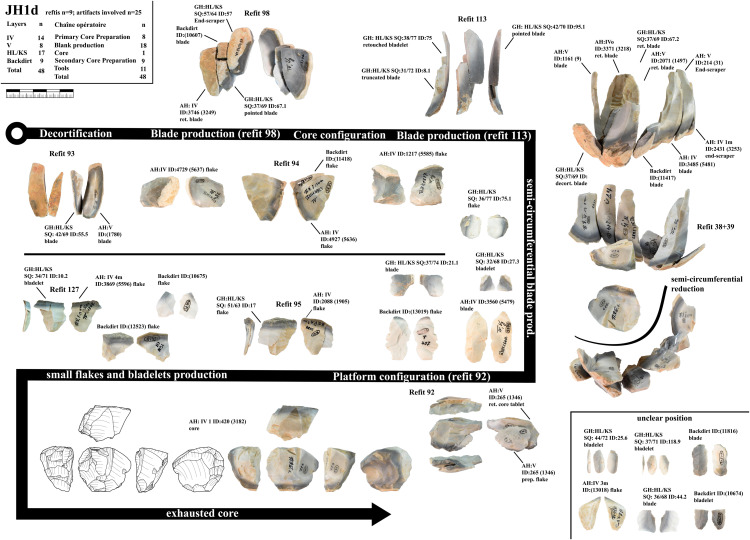
Vogelherd. Minimal raw material unit JH1d. The 48 artifacts and refits are used to reconstruct the reduction process (Photos and drawings: B. Schürch).

***JH1d*:** We could identify 48 artifacts from this unit, which is characterized by a light brown cortex and a characteristic black band under the cortex as well as different shades of gray in the center of the nodule (Fig 3). 25 artifacts could be refitted back together in nine refits (S10–S12 Figs). One core and eleven tools are present in this unit. The reduction of this unit started with the decortification and blade production visible in refit 93 and 98. Three tools are present in refit 98 (retouched blade, end-scraper, pointed blade). The end-scraper from refit 98 (GH:HL/KS SQ:57/64 ID:57) was analyzed for use and hafting traces. The piece showed traces of hide working and hafting traces as well as intense resharpening that removed most use traces. After this first reduction and decortification more blades were reduced (refit 113) and modified. This happened before the production of the pieces in refit 38 + 39, which can be traced by inclusions and black banding in the raw material. Then the blade sequence (refit 38 + 39) was started which shows a semi-circumferential reduction. We positioned the core behind this blade sequence according to the black banding and cortex on the core, the distance to refit 38 + 39 is however not re-constructable. Preparation of the striking platform (refit 94, 92) lead to a continuous shrinking of the core. The last sequence that we can reconstruct is the reduction of bladelets and flakes (refit 95 and refit 127). Due to the black banding and different colored zones in the bladelets, they can be directly connected to the last reduction visible on the core. We, therefore, reconstruct a continuous reduction from blade to bladelets with at least one larger re-configuration of the striking platform, if not more. This reduction resulted in an exhausted core that is difficult to read (to reconstruct the reduction sequence) by itself and is also not easily sorted in one core category. Formal tools produced from this core are retouched blades, end-scrapers and pointed blades.

**Fig 4:**
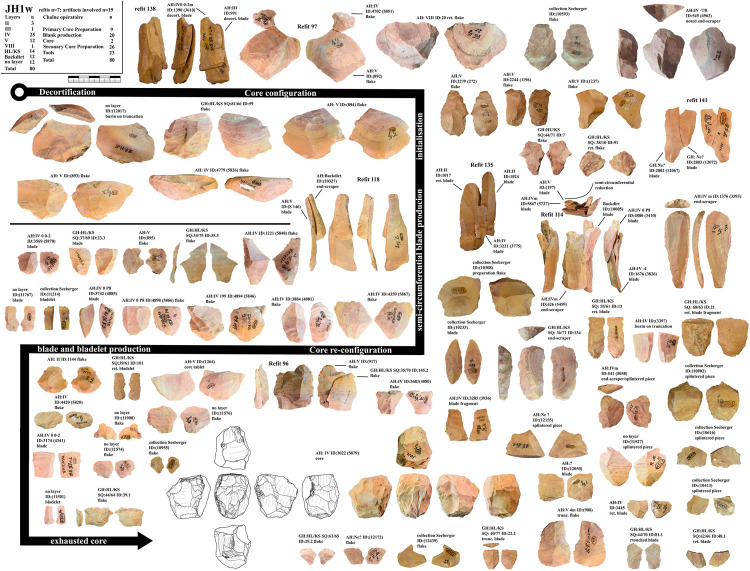
Vogelherd. Minimal raw material unit JH1w. The 80 artifacts and seven refits are used to reconstruct the reduction process (Photos and drawings: B. Schürch).

***JH1w*:** We could identify 80 artifacts from this unit. 19 of them could be refitted back together in five refits (S13–S15 Figs). The minimal raw material unit is characterized by a brown cortex and a banding that is characterized by the alternation of brown, beige, and red bands as well as a pink and gray inclusion in the center of the nodule (Fig 4). There is also a gray, coarse-grained inclusion on some artifacts that is characteristic. Two cores and ten tools are present in this unit. Most of the artifacts come from AH IV and V. Three artifacts originate from AH II, one from III and one from AH VIII. The start of the production cannot be reconstructed. However, refit 138 with two refitted blades shows the decortification process and several large configuration flakes are present, some of which could be refitted (refit 96 and 97). One of this large preparation flakes, a nosed carinated piece (AH:IV ID: 545 (4963)), was utilized for bladelet production. The blade production continued with a crested blade (AH:backdirt ID:(10327)) and the blades in refit 118. The crested blade was likely situated between the narrow and the wide face. This started the semi-circumferential reduction documented in refit 114. End-scrapers from these two refits and another end-scraper were analyzed for hafting and use traces (AH:IVm ID:626 (5459); AH:IVm ID:1376 (3393); backdirt collection Seeberger ID:(10327)). These all showed hafting traces and traces of hide working. Due to the refits, we also could reconstruct the original length of the end-scrapers. The presence of end-scrapers, splintered pieces and a burin point towards different tasks conducted with the artifacts from this unit as well does the bladelet production from a carinated nosed end-scraper. Again, the core (AH:IV ID:3022 (5879)) from this unit does not allow for a reconstruction of the reduction sequence without the refits and the data from the minimal raw material unit. The direction of the reduction was changed at least once. The core in its current state documents the re-configuration and a preparation of a crest. However, this was unsuccessful due to its cuboid shape. If the re-configuration was successful, there would have been a sequence of bladelet production. Formal tools produced from this core are retouched blades, end-scrapers, a burin and splintered pieces

**Fig 5:**
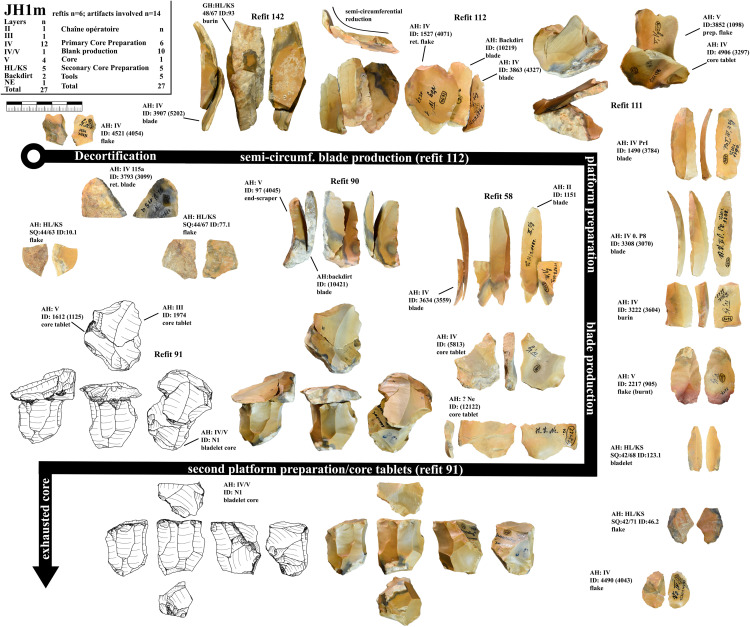
Vogelherd. Minimal raw material unit JH1m. The 27 artifacts and refits are used to reconstruct the reduction process (Photos and drawings: B. Schürch).

***JH1m*:** We could identify 27 artifacts from this unit (Fig 5). 14 of them could be refitted back together in five refits (S16–S18 Figs). One core and four tools are present in this unit. Most of the artifacts come from AH IV, but AH V; IV/V; III and II are also present. The raw material unit is characterized by its brown, beige and gray color variation, a white cortex, and yellow-beige and black inclusions under the cortex with a gray zone in the center of the nodule. For this unit, we can reconstruct three separate sequences of blade removals. First, we can reconstruct the production of blanks and tools in refit 90 and refit 142, where much of the cortex is still present. It is followed by the reduction of pieces in refit 58 and 112. The core platform was re-configured several times during the reduction of these blade sequences, which is testified by refit 111 and 91 as well as two other core tablets (AH:IV ID:(5813); AH:? Ne ID:(12122)). This led to a shrinking of the length of the core. In this case two core tablets were directly refitted to the core (refit 91). Refit 112 and refit 90 also testify to the semi-circumferential reduction of the blade sequences. The core produced small blades and large bladelets in the last reduction. The core was probably discarded due to two prominent hinges on the reduction surface. For this raw material unit five tools (one end-scraper, two burins and two lateral retouched blanks) are present.

**Fig 6:**
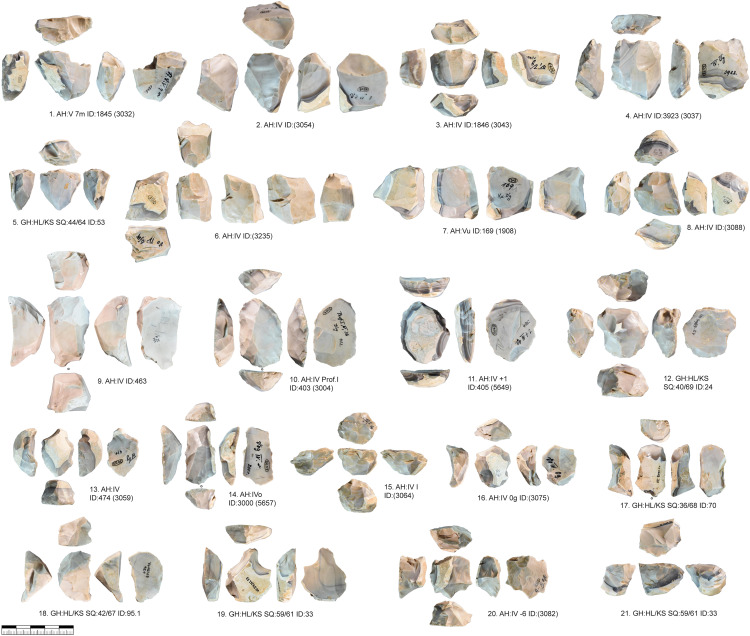
Vogelherd. Cores of raw material unit JH1a: 1-3, 5, 8 semi-circumferential cores; 6-7. multi-platform cores; 5. wide-faced flat core; 9-21. carinated cores (Photos: B. Schürch).

***JH1a*:** This raw material unit contains 17 cores from layer IV and V and eight more cores from the backdirt (Fig 6 and 9, 1–3). The unit does, therefore, not reflect one reduction event but several. This unit contains a total of 138 pieces, including blanks and tools, from layers IV and V. The material of this unit is very fine-grained and does not contain inclusions that could have a negative impact on reduction. The gray material has a characteristic dark gray banding under the cortex. This high quality raw material probably originates from Abensberg Arnhofen, located about 127 km east of Vogelherd, as results using IR-spectroscopy [106] and macroscopic determinations were implying. The core types from this unit are diverse (truncated faceted, semi-circumferential, wide faced flat, multi-platform or carinated). All cores from this unit, however, are relatively small and are no larger than 40 mm (length of the reduction surface). Blades from this unit, with a length up to 75 mm, are often longer than the reduction surface. For most cores we can see a well-planned bladelet production, only in a few cases blade negatives are preserved due to the intense reduction. The semi-circumferential cores demonstrate, that bladelets are not only produced on carinated but also non carinated cores. The cores of this unit are reduced to the maximum and larger flakes are used for bladelet production with carinated and truncated-faceted cores.

#### Carinated pieces.

**Fig 7:**
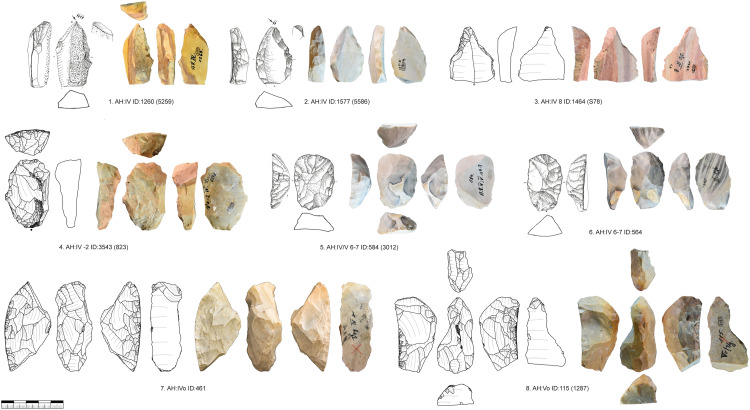
Vogelherd. Carinated pieces. 1-2. Busked burins; 3. Nosed end-scraper; 4. Carinated end-scraper on core tablet; 5-8. Carinated end-scrapers with one or two carinated ends. 2, 5-8 Jurassic chert; 1, 3-4 Brown Jurassic chert (Photos: B. Schürch; Drawings: 1,2,5,6 modified after Hahn 1977; 3-4,7-8 B. Schürch).

To separate the carinated pieces (cores) from the end-scrapers (tools) we conducted a separate study [82]. The result of this traceological study was the identification of a threshold of the thickness of the scraper head at 12 mm between carinated pieces and end-scrapers. Carinated burins showed no traces of use and are therefore all categorized as cores. There are 163 carinated pieces in the assemblages of layer IV and V with 186 core ends (Fig 7). Carinated end-scrapers (including carinated nosed end-scrapers) with 145 pieces (78,38%) make up the majority of the carinated core assemblage (Table 11). Carinated burins (including all subtypes) with 40 pieces (21,62%) are less common.

**Table 11:** Vogelherd. Carinated cores broken down by type and carinated core ends. The n of the carinated core ends is higher than for the core types.

Carinated core types	IV	IV/V	V	Total n	%
**Carinated end-scraper**	49	4	20	74	45,12%
**Nosed end-scraper**	22		3	25	15,24%
**Carinated burin**	16	1	2	19	11,59%
**Carinated end-scraper/carinated end-scraper**	7		1	8	4,88%
**Carinated end-scraper/nosed end-scraper**	6		1	7	4,27%
**Carinated end-scraper/truncated-faceted**	5		2	7	4,27%
**Vachons burin**	5			5	3,05%
**Carinated burin/carinated burin**	2	1	1	4	2,44%
**Carinated end-scraper/carinated burin**	2		2	4	2,44%
**Busked burin**	3			3	1,83%
**Preform carinated end-scraper**	1		2	3	1,83%
**Nosed end-scraper/nosed end-scraper**	1			1	0,61%
**Vachons burin/nosed end-scraper**	1			1	0,61%
**Vachons burin/busked burin**	1			1	0,61%
**Carinated end-scraper/carinated burin/trunc.-faceted**	1			1	0,61%
**Busked burin/truncated-faceted core**	1			1	0,61%
**Vachons burin/truncated-faceted core**	1			1	0,61%
**Total**	**124**	**6**	**34**	**164**	**100%**
**Carinated core ends**	**IV**	**IV/V**	**V**	**Total n**	**%**
**Carinated end-scraper**	77	4	27	108	58,06%
**Nosed end-scraper**	30		4	34	18,28%
**Carinated burin**	20	3	6	29	15,59%
**Busked burin**	4			4	2,15%
**Vachon burin**	8			8	4,30%
**Preform carinated end-scraper**	1		2	3	1,61%
**Total**	**140**	**7**	**39**	**186**	**100%**

**Fig 8:**
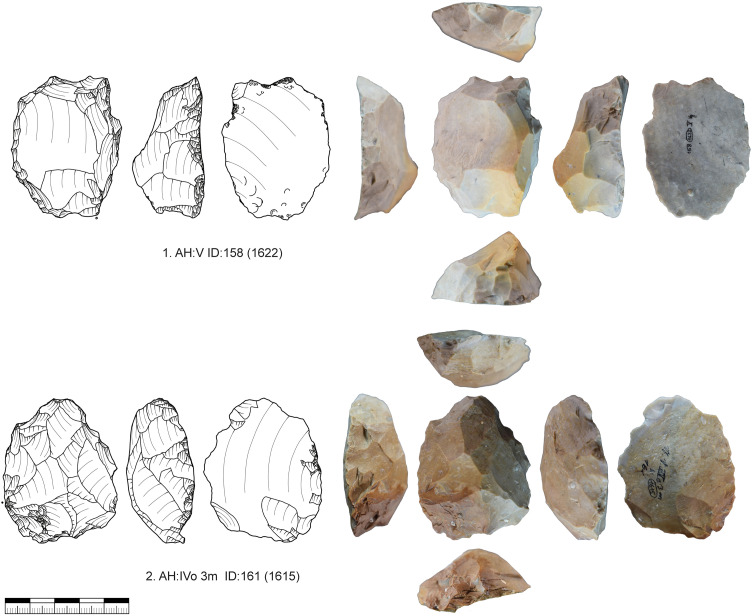
Vogelherd. Preforms of carinated end-scrapers. 1. Preform of a double ended carinated end-scraper with remnant of a crest (dorsal) and several impact rings on the ventral face; 2. Preform of a double ended carinated end-scraper with several hinges (dorsal) and platform preparation; 1-2. Jurassic chert (Photo & drawings: B. Schürch).

The size-variability of carinated end-scrapers and carinated burins is quite large. The smaller carinated end-scrapers are just over the 12 mm threshold and the biggest are over 50 mm in scraper head thickness. This leads to a wide variety of the size and shape of bladelets produced from these cores. We identified two preforms of carinated end-scrapers (Fig 8). These are mostly showing lateral preparation of the carinated ends and one shows several *eraillure* scars (Fig 8, 1). The very thick carinated cores (> 40 mm thickness of the scraper head) were mostly produced on nodules or large frost debris. The smaller and medium sized ones are mostly produced on blades or flakes (Table 12).

**Table 12:** Vogelherd. Blanks of carinated pieces.

Blanks of carinated pieces	IV	IV/V	V	Total n
**Nodule/slab**	**18**	**1**	**6**	**25**
**Debris**	**3**	**2**	**1**	**6**
**Flakes**	**64**	**3**	**14**	**81**
**Decortification flake (~75–100% cortex)**	5		1	6
**Crested flake**	1			1
**Core tablet**	1			1
**Other flakes**	57	3	13	73
**Blades**	**37**		**13**	**50**
**Decortification blade (~75–100% cortex)**	1			1
**Crested blade**	5		1	6
**Core tablet**			1	1
**Other blades**	31		11	42
**Bladelet (burin spall)**	1			1
**Total**	**123**	**6**	**34**	**163**

**Fig 9:**
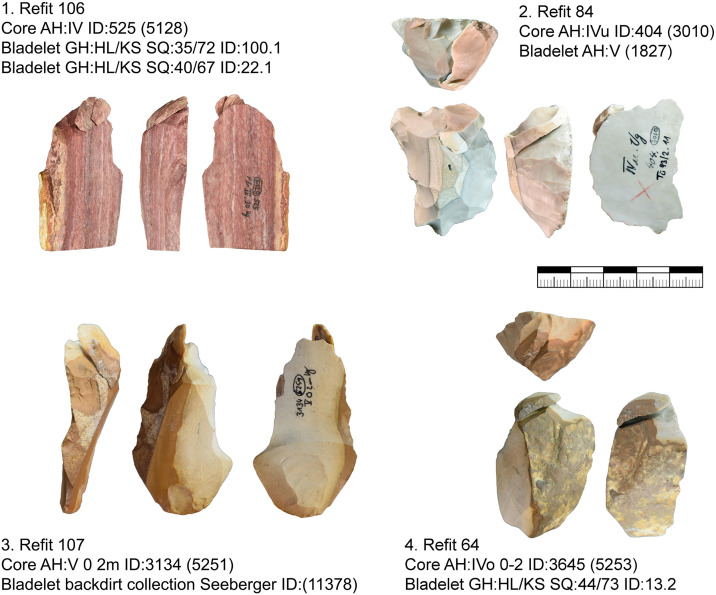
Vogelherd. Examples of refitted carinated pieces and bladelets (Photos: B. Schürch).

The striking platforms of carinated cores are mostly plain (n = 108) or faceted (n = 53), one is a joint plane, and one is unclear. Negatives of a crest to initiate the reduction were only present in two cases. Preparation of the core base, like for busked burins, was present in 13 cases. In six cases we could refit carinated pieces directly with bladelets (Fig 9). These refits were analyzed in detail in the separate study [82]. Carinated cores or the ends of carinated cores are often combined with a second core end. Most of the time this is a second carinated core end, but it can also be combined with a truncated-faceted end or other core types.

#### Truncated-faceted cores.

**Fig 10:**
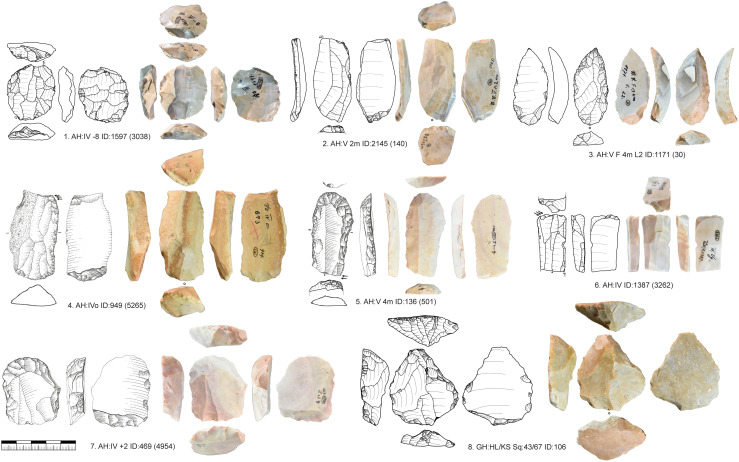
Vogelherd. Truncated-faceted cores. 1. Truncated-faceted core (dorsal) with a Vachon burin like end, 2. Combination of burin on break and truncated-faceted core, 3. Combination of pointed blade and truncated-faceted core, 4. Combination of busked burin and truncated-faceted core, 5. Combination of end-scraper and truncated-faceted core, 6. Combination of truncated-faceted core and burin, 7-8. Combination of carinated piece and truncated-faceted core. 1-3,5-8 Jurassic chert (1-3 from JH1a); 4. Brown Jurassic chert (Photos: B. Schürch; Drawings: 4, 5 and 7 modified after Hahn 1977; 1-3, 6 and 8: B. Schürch).

**Fig 11:**
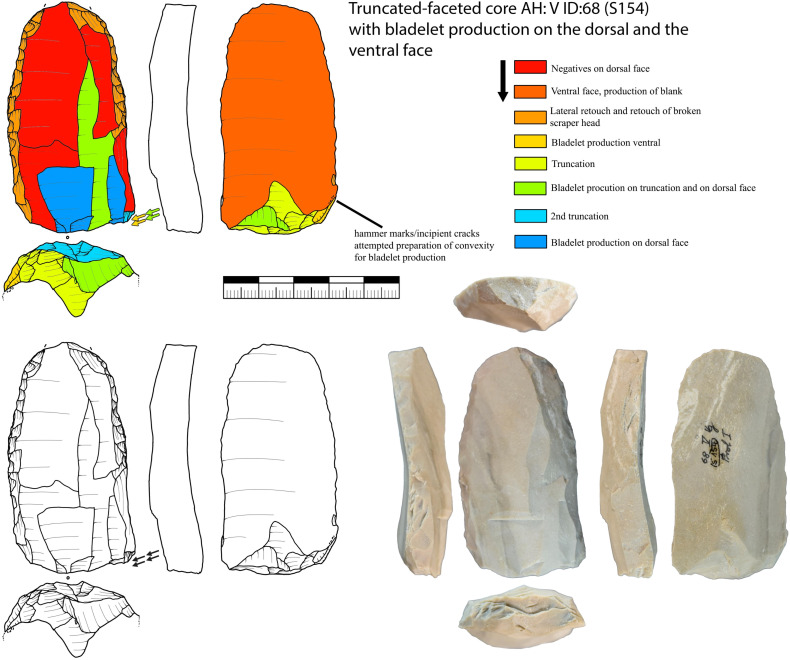
Vogelherd. Working stage analysis of a truncated-faceted core with a broken end-scraper from Jurassic chert. Besides the bladelets produced on the dorsal at least four bladelets were produced on the truncation comparable to a carinated burin (Photo & drawing: B. Schürch).

Truncated-faceted cores (n = 34; IV = 26, V = 8) as described above have one faceted striking platform on the ventral face of a blank (Fig 10). All these cores were configured on blanks (blade or flake). Often truncated-faceted core ends are combined with a carinated end-scraper (n = 8) or a tool end (n = 16). On the dorsal surface straight and thin bladelets are produced. The ridges on the dorsal side of the blank are used to initiate this reduction. The faceted striking platform is in some cases also used to produce smaller twisted bladelets comparable to a carinated burin. From one example (Fig 11) we can reconstruct that first a carinated burin like production was conducted, as a second step the truncation was renewed and bladelets were produced from the dorsal surface. As a last step, very short and wide bladelets were produced again like from a carinated burin. This alternating reduction between the reduction of bladelets on the dorsal face and a carinated burin like reduction is also visible for other pieces (Fig 10; 1, 5, 6). The truncation, therefore, enables two possibilities of bladelet production. For one the production of long straight bladelets on the dorsal face and for an other the production of smaller and twisted bladelets on the truncation. By renewing the truncation, the angle of reduction on the dorsal surface can be reconfigured and a new convexity may be prepared at the same time. The truncated-faceted reduction of Vogelherd enables bladelet production using the width, thickness and the length of the blank, leading to extremely reduced and prepared cores (Fig 10, 1). For some of the artifacts it also seems possible that the truncated end was used to thin the blank for hafting purposes. However, for some of the artifacts it was possible to reconstruct that the truncation happened after the configuration of the opposite end. Some of the truncated-faceted cores were also analyzed in a traceological study [82]. The results indicate that the truncated-faceted ends had no tool or hafting function. For now, the technological evidence points towards a core function.

#### Low quality raw materials.

**Fig 12:**
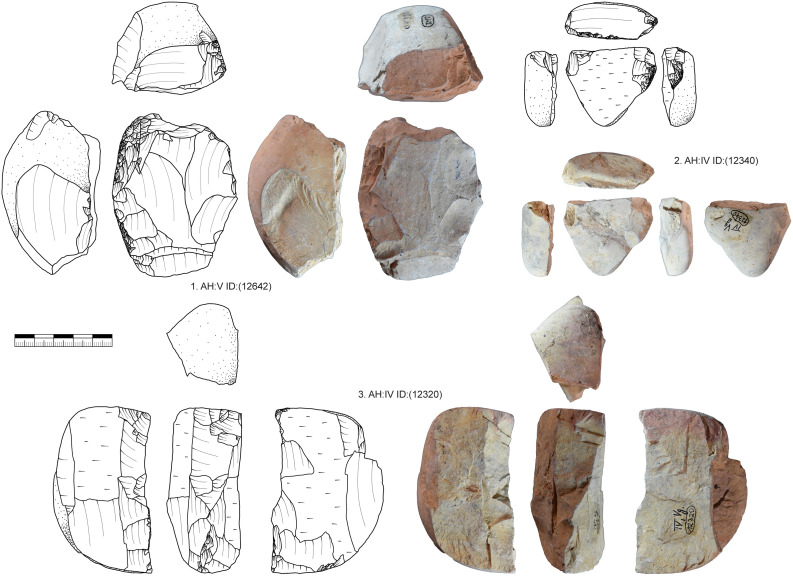
Vogelherd. Prepared limestone cores. 1. Prepared core with crest and showing previous removals of large flakes. 2. Core with a partially prepared crest and two hinges that stopped the reduction. 3. Core with a prepared crest and several hinges of failed blade production (Photo & drawing: B. Schürch).

The presence of cores made from low quality raw materials and especially limestone (n = 18) presents us with several questions (Fig 12). Besides limestone the other low-quality material is very coarse-grained Jurassic chert and Keuper chert as well as Radiolarite with inclusions and natural cracks. From these, limestone can be best separated from the higher quality materials. For Middle or Upper Paleolithic sites of the Swabian Jura limestone is not a common raw material used for lithic production besides for hammerstones. The use as hammerstones is also common in other regions [e.g., 110]. However, limestone plays a certain role in the assemblage of Vogelherd, which is also confirmed by the amount of this material in layer IV and V (n = 219; 3,84%). Of the 219 limestone artifacts 100 are present in the form of nodules. 43 are used as hammerstones, retouchers or splintered pieces. No limestone artifacts are knapped formal tools. 63 limestone flakes are present in the assemblage. The core types made of limestone are prepared cores (n = 8), tested cores (n = 4), multi-platform (n = 2) or unknown/broken (n = 4). The amount of unfinished or only prepared cores is very high. Even though limestone does not have the same properties as Jurassic chert and the flakes produced do not have the same properties as chert flakes, the limestone was used for knapping. However, none of the 63 limestone blanks are blades, all are flakes.

#### Percussion technique.

The two main sources used for the reconstruction of the percussion technique are the platform attributes and the hammerstones and organic hammers in the assemblage. The hardness of the hammer is determined by a combination of different attributes for blanks defined by Pelegrin [111,112]. These are platform type, striking remnant modification, width & thickness of striking remnant, pronunciation of bulb, bulbar scar, lip, Hertzian cone fracture and exterior platform angle. Using the criteria of Pelegrin, hard hammer, soft organic hammer and soft hammer percussion are present at Vogelherd. For blades and bladelets soft organic hammer and soft hammer are most common. For removing decortification and configuration flakes hard hammer is common. Other experimental studies suggest that Pelegrin’s separation of percussion techniques cannot reliably determine the hammer [113]. Pelegrin’s experiments have also not yet been carried out on the here analyzed Jurassic chert. Weighing up the various arguments, in our opinion, it is not always possible to make a clear distinction between the percussion techniques.

**Fig 13:**
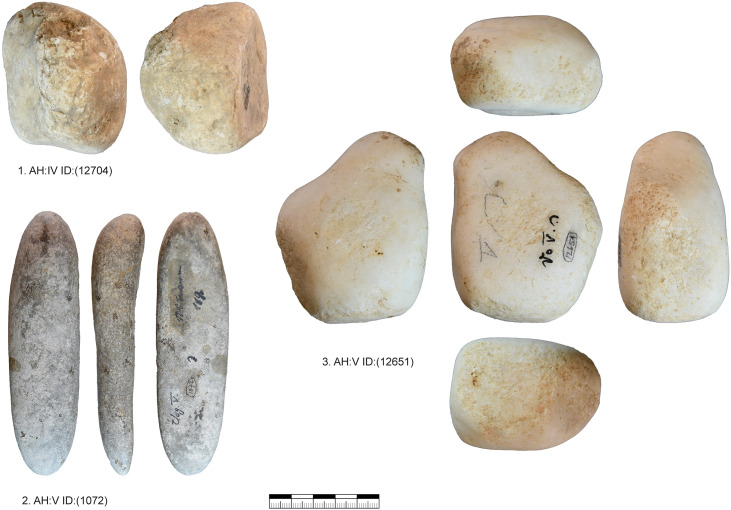
Vogelherd. Hammerstone and retouchers. 1. Limestone hammer stone, 2. Retoucher from undetermined river pebble; 3. Quartz hammer stone (Photo: B. Schürch).

In addition to the percussion technique reconstructed from blade and bladelet attributes which is associated with a particular degree of imprecision [113], we also list the different hammers/retouchers found in the assemblage (Table 13). In total 95 hammerstones and three stone retouchers were recovered from the Aurignacian of Vogelherd (Fig 13). The most used raw material is limestone, quartz, and quartzite. A large portion cannot be determined, without further detailed analysis, these are subsumed under the category undetermined river pebbles. Therefore, both hard stone hammer and soft mineral hammer are present in the assemblage. In addition Niven [114] identified 148 organic retouchers made from bone, teeth and ivory from the assemblage of layer IV, VI/V and V. A subsample was recently analyzed by Toniato [115] and before by Taute [116].

**Table 13:** Vogelherd. Lithic raw material of hammerstones and retouchers.

Raw material	n hammerstones	n retouchers
**Limestone**	33	1
**Undetermined river pebble**	30	2
**Quartz**	11	
**Quartzite**	8	
**Sandstone**	4	
**Other/undetermined**	4	
**Radiolarite**	3	
**Keuper chert**	2	
**Jurassic chert**	1	
**Total**	96	3

### Metric analysis of cores, blades and bladelets

We recorded several metric and discrete attributes for all cores from Vogelherd (S4 Table). These attributes enable us to discuss the intensity of reduction (e.g., size, reduction angle) and also show us the size before the reduction (tested cores). We also measured the largest target negative on each core, if present and determinable. By comparing these attributes to the sizes of blades and bladelets we can gain further insights into the reduction process (see discussion). To present and discuss the data we mainly use the median of the attributes. However, we also give other values (mean, standard error, standard deviation, variance, span, minimum and maximum) to show the variability of cores. The statistics for all cores show the dimensions. We present the core measurements with and without carinated and truncated-faceted cores to enable comparisons with Geißenklösterle, because Hahn [12] did not list the carinated pieces and truncated-faceted cores (Kostienki) in his study. We added one table with all the attributes broken down by core type.

#### Size differences between core types.

One obvious difference between the different core types is that the cores that only produced bladelets (carinated pieces and truncated-faceted cores) have smaller reduction surfaces, are mostly produced on smaller blanks or nodules, which influences the size. From the minimum values of the width of the largest target negative, we can see that most core types produced bladelets. Production of small bladelets (width smaller than 7 mm) is most pronounced for carinated pieces and truncated-faceted cores (Table 14). However, the median width of target blanks for most core types (narrow-sided = 10,5 mm; semi-circumferential = 12,5 mm; wide-faced-flat = 12,8 mm) show that the last reduction steps often removed small blades or bladelets. Narrow sided, carinated and truncated cores generally have reduction surfaces with a smaller width. Another difference that stands out is that the reduction angles of the carinated pieces are smaller (Table 15). Tested cores tend to be larger and heavier than the other types. Other differences corresponding to the core classification are visible in the metric attributes and were tested with a Kruskal-Wallis test (S2 Table).

**Table 14:** Vogelherd. Overview of metric core attributes with and without carinated pieces and truncated-faceted cores.

Cores with truncated-faceted cores and carinated pieces											
		Nodule/blank length in mm	Blank/ nodule width in mm	Blank/ nodule thickness in mm	Weight in g	Reduction face length in mm	Reduction face width in mm	Reduction surface thickness in mm	Reduction angle in °	Width largest target negative in mm	Length largest target negative in mm
n	present	424	424	424	422	406	407	404	369	286	279
	absent	0	0	0	2	18	17	20	55	138	145
Mean		47,3	36,8	25,8	74,6	33,9	30,6	27,8	74,6	11,7	27,0
Standard error of mean		0,8	0,8	0,9	7,4	0,9	0,9	1,0	0,6	0,6	0,8
Median		**43,8**	**33,7**	**19,9**	**29,3**	**31,6**	**27,5**	**21,3**	**75,0**	**8,5**	**23,6**
Std. deviation		17,2	15,9	18,2	151,9	18,5	18,1	19,2	12,4	9,9	13,5
Variance		295,9	253,4	331,5	23088,4	340,5	329,0	370,4	152,7	97,5	181,0
Span		105,4	99,6	113,5	1542,1	114,5	103,6	116,1	68,0	63,9	90,9
Minimum		14,8	6,9	3,0	2,2	5,6	2,9	3,2	42,0	1,3	6,1
Maximum		120,1	106,5	116,5	1544,3	120,1	106,5	119,3	110,0	65,2	97,0
**Cores without truncated-faceted cores and carinated pieces**											
		**Nodule/blank length in mm**	**Blank/ nodule width in mm**	**Blank/ nodule thickness in mm**	**Weight in g**	**Reduction face length in mm**	**Reduction face width in mm**	**Reduction surface thickness in mm**	**Reduction angle in °**	**Width largest target negative in mm**	**Length largest target negative in mm**
n	present	215	215	215	214	208	208	208	204	132	122
	absent	0	0	0	1	7	7	7	11	83	93
Mean		48,8	42,7	35,0	122,3	45,2	40,1	37,3	80,5	17,4	35,5
Standard error of mean		1,4	1,2	1,4	13,8	1,2	1,4	1,5	0,7	1,0	1,3
Median		**44,2**	**38,3**	**29,3**	**48,3**	**41,8**	**36,5**	**32,4**	**80,5**	**14,4**	**33,7**
Std. deviation		20,1	18,3	20,4	201,3	17,9	19,6	21,4	10,7	11,9	14,1
Variance		405,5	334,2	418,0	40503,6	318,8	382,3	458,8	113,7	141,8	199,4
Span		105,4	99,6	107,3	1538,9	105,7	99,6	108,1	64,0	62,6	86,7
Minimum		14,8	6,9	9,2	5,4	14,4	6,9	11,2	46,0	2,6	10,3
Maximum		120,1	106,5	116,5	1544,3	120,1	106,5	119,3	110,0	65,2	97,0

**Table 15:** Vogelherd. Overview of metric core attributes for the most important core types.

Cores												
Core types			Nodule/blank length in mm	Blank/nodule width in mm	Blank/nodule thickness in mm	Weight in g	Reduction face length in mm	Reduction face width in mm	Reduction surface thickness in mm	Reduction angle in °	Width largest target negative in mm	Length largest target negative in mm
**Carinated**	n	Present	184	184	184	183	183	183	180	154	144	147
		Absent	0	0	0	1	1	1	4	30	40	37
	Mean		45,0	31,5	17,3	27,0	21,7	20,7	18,6	67,2	6,7	20,1
	Standard error of mean		1,0	0,7	0,6	1,7	0,6	0,7	0,7	0,8	0,2	0,7
	Median		**43,0**	**29,8**	**15,2**	**20,1**	**19,6**	**19,4**	**15,5**	**67,0**	**6,2**	**18,7**
	Std. deviation		13,2	10,1	8,2	23,4	8,7	8,8	9,1	10,4	2,7	8,1
	Variance		173,2	102,6	68,0	546,2	76,4	77,9	83,7	108,1	7,4	65,1
	Span		73,0	73,1	79,7	230,4	45,9	39,8	59,5	52,0	15,3	42,9
	Minimum		16,6	7,9	5,6	2,2	5,6	4,7	6,0	42,0	1,3	6,1
	Maximum		89,5	81,0	85,3	232,6	51,5	44,5	65,5	94,0	16,6	49,0
**Flake Core**	n	Present	19	19	19	19	19	19	19	19	13	13
		Absent	0	0	0	0	0	0	0	0	6	6
	Mean		40,8	41,5	18,2	30,8	41,0	39,5	18,0	78,0	23,4	25,9
	Standard error of mean		2,1	2,6	1,0	3,6	1,9	2,7	1,0	2,1	2,1	2,1
	Median		**39,8**	**39,5**	**17,0**	**29,2**	**40,2**	**37,9**	**16,6**	**78,0**	**21,3**	**25,6**
	Std. deviation		9,0	11,4	4,5	15,7	8,1	11,9	4,5	9,3	7,5	7,6
	Variance		80,8	130,8	20,1	245,8	65,1	142,1	19,9	86,9	56,7	57,5
	Span		32,9	42,8	15,6	63,1	30,6	42,8	15,2	35,0	25,3	25,0
	Minimum		27,1	22,4	11,3	9,1	29,4	22,4	11,7	60,0	13,3	13,7
	Maximum		60,0	65,2	26,9	72,2	60,0	65,2	26,9	95,0	38,5	38,6
**Multi-platform**	n	Present	23	23	23	23	22	22	22	21	16	14
		Absent	0	0	0	0	1	1	1	2	7	9
	Mean		44,3	45,0	38,3	113,9	41,2	43,6	39,1	85,6	18,5	32,8
	Standard error of mean		2,7	4,3	3,2	28,0	2,9	4,5	3,2	1,7	2,4	3,3
	Median		**44,2**	**40,3**	**36,4**	**73,9**	**43,6**	**40,0**	**35,5**	**86,0**	**17,1**	**32,7**
	Std. deviation		13,1	20,4	15,4	134,1	13,5	21,3	15,2	7,7	9,5	12,3
	Variance		171,4	416,9	236,8	17986,2	182,2	451,6	231,9	59,3	90,7	150,6
	Span		47,1	83,8	67,7	567,3	59,1	89,6	67,7	35,0	45,9	43,2
	Minimum		26,8	22,7	21,3	18,9	14,8	16,8	21,3	70,0	3,4	19,0
	Maximum		73,9	106,5	89,0	586,2	73,9	106,5	89,0	105,0	49,3	62,2
**Narrow-sided**	n	Present	33	33	33	33	33	33	33	32	29	30
		Absent	0	0	0	0	0	0	0	1	4	3
	Mean		51,1	33,2	33,6	72,4	46,2	25,5	36,7	78,8	12,0	40,5
	Standard error of mean		3,2	3,0	2,2	16,2	2,2	1,8	2,1	1,9	1,4	2,5
	Median		**50,1**	**29,7**	**30,9**	**49,9**	**44,9**	**24,0**	**36,2**	**80,0**	**10,5**	**41,9**
	Std. deviation		18,2	17,1	12,6	93,2	12,8	10,6	12,3	10,9	7,5	13,5
	Variance		332,0	291,0	158,9	8694,8	163,9	111,4	152,3	118,2	55,8	181,6
	Span		94,6	87,4	48,8	522,5	56,7	47,4	49,4	46,0	34,1	59,5
	Minimum		21,4	6,9	15,5	8,7	21,4	6,9	14,6	57,0	3,7	18,6
	Maximum		115,9	94,2	64,3	531,2	78,0	54,2	64,0	103,0	37,8	78,0
**Other**	n	Present	6	6	6	6	5	5	5	6	5	5
		Absent	0	0	0	0	1	1	1	0	1	1
	Mean		52,4	55,1	38,9	214,7	45,1	57,6	50,3	71,7	27,9	38,2
	Standard error of mean		9,0	8,3	12,5	90,4	4,0	14,7	11,4	3,5	9,3	2,5
	Median		**46,6**	**55,5**	**30,6**	**189,3**	**41,2**	**73,5**	**55,4**	**67,5**	**18,7**	**35,4**
	Std. deviation		22,0	20,4	30,5	221,4	8,8	32,8	25,4	8,5	20,9	5,7
	Variance		483,5	417,8	931,0	49013,8	78,2	1076,0	646,9	72,3	436,8	32,0
	Span		59,6	39,1	73,8	512,3	20,2	81,2	67,7	19,0	43,2	12,9
	Minimum		34,0	35,1	11,6	11,9	35,4	10,6	13,5	64,0	9,4	32,1
	Maximum		93,6	74,2	85,4	524,2	55,6	91,8	81,2	83,0	52,6	45,0
**Prepared core**	n	Present	29	29	29	28	28	28	28	27	4	4
		Absent	0	0	0	1	1	1	1	2	25	25
	Mean		56,4	38,1	39,2	114,9	54,7	34,3	42,9	81,4	27,4	30,0
	Standard error of mean		4,0	2,7	3,5	23,4	3,9	3,0	3,2	1,7	9,7	3,5
	Median		**50,2**	**36,8**	**37,0**	**62,6**	**48,3**	**35,3**	**40,9**	**80,0**	**26,8**	**29,8**
	Std. deviation		21,6	14,6	18,6	123,8	20,8	15,7	16,9	8,8	19,4	7,0
	Variance		465,6	212,6	347,3	15329,4	431,5	245,1	286,1	76,7	377,1	49,0
	Span		71,8	68,5	72,0	565,9	75,7	63,9	66,7	42,0	43,6	13,1
	Minimum		33,9	14,1	13,0	22,9	30,0	11,7	18,3	68,0	6,2	23,7
	Maximum		105,7	82,6	85,0	588,8	105,7	75,6	85,0	110,0	49,8	36,8
**Semi-circumferential**	n	Present	45	45	45	45	44	44	44	44	39	33
		Absent	0	0	0	0	1	1	1	1	6	12
	Mean		41,2	38,5	29,7	59,7	40,4	38,4	30,4	80,7	13,1	34,9
	Standard error of mean		1,7	1,8	1,4	8,4	1,7	1,9	1,4	1,5	0,9	2,2
	Median		**39,6**	**36,2**	**28,4**	**39,1**	**38,4**	**36,4**	**29,4**	**82,0**	**12,2**	**31,6**
	Std. deviation		11,3	11,8	9,6	56,6	11,5	12,6	9,5	10,1	5,5	12,5
	Variance		128,8	139,9	92,1	3200,2	131,7	159,1	90,6	102,1	30,3	155,2
	Span		57,9	63,9	39,2	281,2	55,9	75,9	38,2	46,0	25,2	50,2
	Minimum		16,1	19,9	10,3	9,3	16,1	9,5	11,2	52,0	3,0	16,1
	Maximum		74,0	83,8	49,5	290,5	72,0	85,3	49,5	98,0	28,1	66,3
**Tested core**	n	Present	18	18	18	18	18	18	18	18	1	1
		Absent	0	0	0	0	0	0	0	0	17	17
	Mean		76,0	65,2	72,3	469,1	63,8	64,6	76,0	85,9	43,9	35,1
	Standard error of mean		5,8	4,1	6,7	80,8	6,0	4,7	6,9	2,3		
	Median		**78,3**	**68,4**	**73,4**	**355,1**	**63,8**	**62,7**	**78,3**	**87,0**	**43,9**	**35,1**
	Std. deviation		24,8	17,5	28,6	342,7	25,6	20,0	29,3	9,8		
	Variance		613,3	306,5	816,8	117438,3	654,3	400,0	857,5	95,6		
	Span		88,1	63,6	95,7	1073,9	94,6	68,2	98,5	43,0	0,0	0,0
	Minimum		32,0	29,1	20,8	88,1	25,5	29,1	20,8	62,0	43,9	35,1
	Maximum		120,1	92,7	116,5	1162,0	120,1	97,3	119,3	105,0	43,9	35,1
**Truncated-faceted**	n	Present	25	25	25	25	15	16	16	12	10	11
		Absent	0	0	0	0	10	9	9	13	15	14
	Mean		50,3	25,6	9,8	14,7	25,6	19,6	9,9	71,2	8,8	24,0
	Standard error of mean		2,9	1,3	0,8	2,1	3,9	2,5	0,8	2,9	1,2	2,6
	Median		**52,3**	**25,0**	**8,5**	**10,8**	**25,5**	**15,6**	**9,7**	**72,0**	**9,1**	**23,3**
	Std. deviation		14,5	6,7	3,8	10,6	15,1	10,0	3,2	10,2	3,7	8,6
	Variance		210,1	45,3	14,1	112,4	227,7	99,6	10,1	104,2	14,0	74,6
	Span		59,1	21,0	14,0	37,2	55,0	33,9	12,7	34,0	12,2	32,7
	Minimum		29,3	16,0	3,0	4,5	7,9	2,9	3,2	54,0	2,9	7,9
	Maximum		88,4	37,0	17,0	41,7	62,9	36,8	15,9	88,0	15,1	40,6
**unknown-broken**	n	Present	30	30	30	30	27	27	27	25	14	12
		Absent	0	0	0	0	3	3	3	5	16	18
	Mean		43,2	45,2	27,2	125,6	37,1	43,3	30,5	77,1	25,0	37,7
	Standard error of mean		4,4	4,0	3,3	52,4	4,1	4,6	4,3	3,0	5,2	6,3
	Median		**34,4**	**38,5**	**25,7**	**30,4**	**32,9**	**36,6**	**26,2**	**73,0**	**19,1**	**32,8**
	Std. deviation		24,0	22,0	17,9	286,9	21,3	24,0	22,5	15,2	19,5	21,9
	Variance		576,6	486,2	318,8	82302,5	453,7	574,4	506,2	232,4	380,2	478,9
	Span		80,9	86,0	94,6	1538,9	81,2	86,0	92,4	62,0	62,6	81,8
	Minimum		14,8	19,3	9,2	5,4	14,4	19,3	11,4	46,0	2,6	15,2
	Maximum		95,6	105,3	103,8	1544,3	95,6	105,3	103,8	108,0	65,2	97,0
**Wide-faced flat**	n	Present	12	12	12	12	12	12	12	11	11	9
		Absent	0	0	0	0	0	0	0	1	1	3
	Mean		45,8	46,6	31,0	98,1	41,9	43,7	29,8	79,0	17,4	39,3
	Standard error of mean		3,9	4,1	4,4	30,3	4,6	4,7	4,7	2,2	3,7	6,5
	Median		**42,4**	**41,4**	**26,3**	**54,3**	**39,5**	**37,8**	**25,2**	**80,0**	**12,8**	**38,5**
	Std. deviation		13,5	14,3	15,4	105,1	16,0	16,4	16,3	7,4	12,2	19,5
	Variance		182,7	203,6	236,5	11043,9	256,2	267,7	267,2	55,2	148,1	381,1
	Span		45,0	46,1	48,9	304,8	56,9	53,1	51,6	22,0	37,3	62,1
	Minimum		28,7	28,3	15,5	18,3	16,8	21,3	12,7	68,0	6,0	10,3
	Maximum		73,7	74,4	64,4	323,1	73,7	74,4	64,4	90,0	43,3	72,4

#### Comparison of dimensions of blade, bladelets and cores.

**Fig 14:**
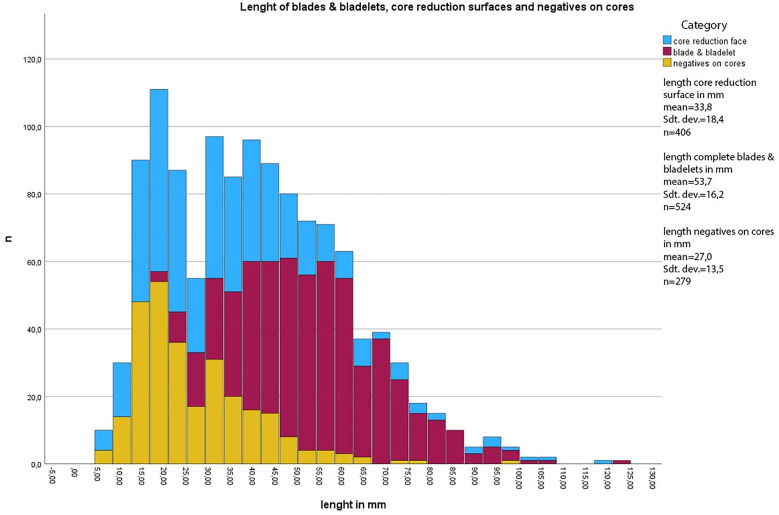
Vogelherd. Length of the core reduction face, blades & bladelets and negatives on cores together.

One of the best indications of the original size of the raw materials are the tested cores. These are most of the time of low quality if large parts of the original volume are preserved. They are larger (length of the reduction face) and heavier (=volume) than all other core types. Although that difference is not strongly pronounced. Other evidence of the original size of the cores comes from comparisons with core negatives and the size distribution of blades and bladelets (see below) (Table 16). To reconstruct the original core size, we combined core measurements with the size of negatives and the size of the blades and bladelets to compare them. It should be noted that bladelets are heavily underrepresented due to the excavation techniques in 1931. For Fig 14 only completely preserved blades and bladelets were included (n = 524). The length of the reduction faces (n = 406) and the size of the negatives on cores (n = 279) are plotted together with the blades and bladelets. The largest blade (length of 122 mm) and the smallest bladelet (length of 18 mm) show the size range of the blanks. Below a length of 25 mm almost no bladelets were collected. Negatives and length of cores give a more realistic insight into the assemblage. Especially the width of the negatives on the cores shows that production of bladelets was of central interest. Fig 14 and Tables 15 and 16 also show that the large blade cores are mostly missing, the large cores preserved are mostly tested cores. This is also supported by length of the target blanks, in this case blades, that are with few exceptions shorter than 65 mm. There is one noticeable dip between 25 and 30 mm length (Fig 14). This is best explained by the differences between carinated and non-carinated blade and bladelet production as carinated cores produce much shorter products than the other cores (Table 15).

**Table 16:** Vogelherd. Comparison of length and width of complete blades & bladelets, negatives on cores and length of core reduction faces. Width of complete blades & bladelets and negatives on cores. The width of core reduction faces is marked in gray because it is not comparable to the other values.

Comparison blades bladelets and core attributes				
Category			Length in mm	Width in mm
Cores reduction face	n	Present	406	407
		Absent	18	17
	Mean		33,9	30,6
	Standard error of mean		0,9	0,9
	Median		**31,6**	27,5
	Std. deviation		18,5	18,1
	Variance		340,5	329,0
	Span		114,5	103,6
	Minimum		5,6	2,9
	Maximum		120,1	106,5
Blades and bladelets	n	Present	524	524
		Absent	2	2
	Mean		53,7	19,5
	Standard error of mean		0,7	0,3
	Median		**53,1**	**19,0**
	Std. deviation		16,3	7,2
	Variance		264,6	51,6
	Span		104,1	62,5
	Minimum		18,4	4,0
	Maximum		122,5	66,5
Negatives on Cores	n	Present	279	286
		Absent	121	114
	Mean		27,0	11,7
	Standard error of mean		0,8	0,6
	Median		**23,6**	**8,5**
	Std. deviation		13,5	9,9
	Variance		181,0	97,5
	Span		90,9	63,9
	Minimum		6,1	1,3
	Maximum		97,0	65,2

The dimensions of core tablets and crested blades can be used to reconstruct a minimum for the original size of cores as well (Table 17). The size of core tablets shows the dimension of the former platform and crested blades give a minimum for the length of the core. The core tablets show that one core tablet removed roughly 12 mm of the former core length on average. Depending on the number of core tablets removed this gives the rate for the decrease in length of the reduction faces. From the refits we could reconstruct that one or two tablets were removed before the production was started again. The length and width of the core tablets also reveal that they were mostly removed from medium large to smaller cores. The length of the crested blades reveal that these can be found on almost all core sizes with an average of 52 mm size spanning from the largest one with 95 mm length to the smallest one with 30 mm length.

**Table 17:** Vogelherd. The size of completely preserved crested blades and core tablets.

Core tablets and crested blades					
Category			Length in mm	Width in mm	Thickness in mm
Core tablet	n	Present	54	54	54
		Absent	0	0	0
	Mean		39,5	39,6	12,1
	Standard error of mean		1,4	2,1	0,8
	Median		**38,4**	**39,7**	**11,7**
	Std. deviation		10,6	15,2	6,1
	Variance		113,4	230,7	37,3
	Span		45,3	82,7	37,7
	Minimum		15,5	8,0	4,3
	Maximum		60,8	90,7	42,0
Crested blade	n	Present	55,0	55,0	55,0
		Absent	0,0	0,0	0,0
	Mean		52,0	21,1	10,0
	Standard error of mean		2,0	0,8	0,4
	Median		**51,4**	**21,3**	**9,7**
	Std. deviation		14,6	5,7	3,1
	Variance		214,1	33,0	9,4
	Span		65,0	26,1	15,1
	Minimum		30,1	9,8	2,5
	Maximum		95,1	35,8	17,6

### Geißenklösterle assemblage

To provide a baseline for metric comparison, we took the data provided in Hahn 1988. The core statistics do not include the carinated and truncated-faceted cores. In total, Hahn analyzed 38 cores from AH II and III (Table 18). We compared the size of the cores from Geißenklösterle and Vogelherd (not including the carinated and truncated faceted cores) with a Mann-Whitney U test (S3 Table). The cores from Geißenklösterle are significantly longer and heavier than the cores from Vogelherd. This could be interpreted as the result of a more intense reduction at Vogelherd, or the use of bigger nodules at Geißenklösterle. Hahn listed the carinated pieces (carinated end-scrapers and thick nosed end-scrapers) separately. For the carinated pieces we used the descriptive statistics done by Hahn. For the other cores, because the measurements are listed for every single piece, we calculated the numbers ourselves. Hahn did not give the measurements of truncated-faceted cores, or as he calls them Kostienki knives, so their measurements were not included here.

**Table 18:** Geißenklösterle. Cores, carinated pieces and thick nosed end-scraper measurements from Hahn 1988.

Cores without carinated pieces, nosed end-scrapers and truncated-faceted cores								
		Nodule/blank length in mm	Blank/nodule width in mm	Blank/nodule thickness in mm	Weight in g	Reduction face length in mm	Reduction face width in mm	Reduction surface thickness in mm
n	Present	38	38	38	36	38	38	38
	Absent	0	0	0	2	0	0	0
Mean		52,2	46,0	36,2	111,9	52,2	46,0	36,2
Standard error of mean		2,2	3,1	2,2	20,3	2,2	3,1	2,2
Median		**52,0**	**43,5**	**34,5**	**76,5**	**52,0**	**43,5**	**34,5**
Std. deviation		13,6	19,0	13,5	121,8	13,6	19,0	13,5
Variance		185,7	361,7	182,6	14833,3	185,7	361,7	182,6
Span		59,0	86,0	64,0	606,0	59,0	86,0	64,0
Minimum		29,0	22,0	15,0	14,0	29,0	22,0	15,0
Maximum		88,0	108,0	79,0	620,0	88,0	108,0	79,0
**Carinated end-scrapers after Hahn 1988**								
		**Nodule/blank length in mm**	**Blank/nodule width in mm**	**Blank/nodule thickness in mm**	**Reduction face width in mm**	**Reduction surface thickness in mm**	**Retouch height in mm**	
n	Present	8,0	8,0	8,0	8,0	8,0	8,0	
	Absent	0,0	0,0	0,0	0,0	0,0	0,0	
Mean		49,2	28,7	19,9	24,8	19,9	25,1	
Std. deviation		13,2	7,3	8,5	8,0	3,4	3,3	
V		26,8	25,3	42,9	32,4	16,8	13,3	
Minimum		37,6	17,8	12,0	14,6	15,6	21,1	
Maximum		82,1	43,3	31,0	39,9	27,0	33,0	
**Thick nosed end-scrapers after Hahn 1988**								
		**Nodule/blank length in mm**	**Blank/nodule width in mm**	**Blank/nodule thickness in mm**	**Reduction face width in mm**	**Reduction surface thickness in mm**	**Retouch height in mm**	
n	Present	9,0	9,0	9,0	9,0	9,0	9,0	
	Absent	0,0	0,0	0,0	0,0	0,0	0,0	
Mean		49,4	32,2	17,1	18,3	15,7	19,9	
Std. deviation		6,8	7,6	3,3	5,9	3,1	2,9	
V		13,7	23,7	19,3	32,1	19,6	14,3	
Minimum		36,8	24,6	11,8	8,0	12,1	15,3	
Maximum		62,5	45,6	22,6	27,5	22,6	26,0	

**Fig 15:**
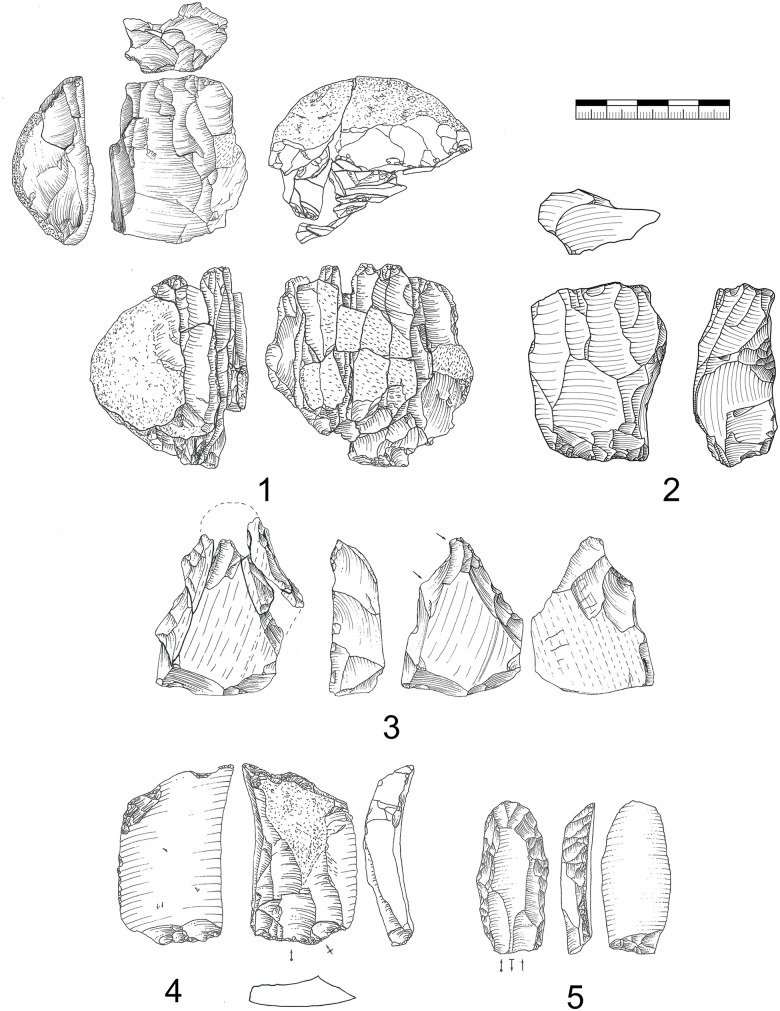
Geißenklösterle. 1. Refits of unit A1, semi-circumferential reduction is visible from the top view mostly from AH III. 2. Semi-circumferential core with a crested back from AH IId (part of AH III). 3. Nosed end-scraper with refitted flakes from AH II and III. 4. Truncated-faceted core from AH IIb. 5. Carinated end-scraper/truncated-faceted core from AH IIb. 1. Radiolarite, 2-5. Jurassic chert (Drawings: 1 and 3-5 after Hahn 1988; 2 S. Schray).

With new analyses still ongoing, we were not able to integrate quantitative data of the refits. We will refer to aspects previously published by former studies and present examples when suitable. We selected two refits that represent the reduction concepts of Geißenklösterle. The first refit is the A1 unit that is comprised by a blade/bladelet core with several refitted blades and bladelets (Fig 15, 1). From the refit of the radiolarite nodule the semi-circumferential reduction can be reconstructed. In this case the core is classified as wide-faced flat and not semi-circumferential due to the trimming of the side of the core. The second refit includes a carinated nosed end-scraper with four refitted configuration flakes (Fig 15, 3). In this specific case the production of bladelets is combined with the production of a bladelet from the former striking surface of the nosed end-scraper and a truncation on the opposite side where two bladelets were removed comparable to a burin.

## Discussion

The presented data on the Aurignacian of Vogelherd Cave enables us to discuss several aspects of Aurignacian core technology and compare the results from Vogelherd, that are without high stratigraphic resolution, with other Aurignacian sites and especially Geißenklösterle. Other sites like Hohle Fels, Keilberg Kirche, Hohlenstein-Stadel, Hohlenstein-Bärenhöhle, Bockstein Törle and Sirgenstein are also important for discussing the technological choices of modern human groups in the Aurignacian of southern Germany [13,14,30,32,65,117]. Therefore, we will present several characteristics of the Aurignacian of Vogelherd in the following and compare them with the other Aurignacian sites of the region.

### Reconstruction of reduction and configuration process

With our results we tried to reconstruct the configuration and reduction processes. The several refits, minimal raw material units and the large number of cores as a source for the reconstruction make it necessary to further discuss the results and add examples to further contextualize the results.

#### Core configuration and fully configurated/prepared cores (semi-circumferential, narrow-sided, wide-faced flat and multi-platform cores).

**Fig 16:**
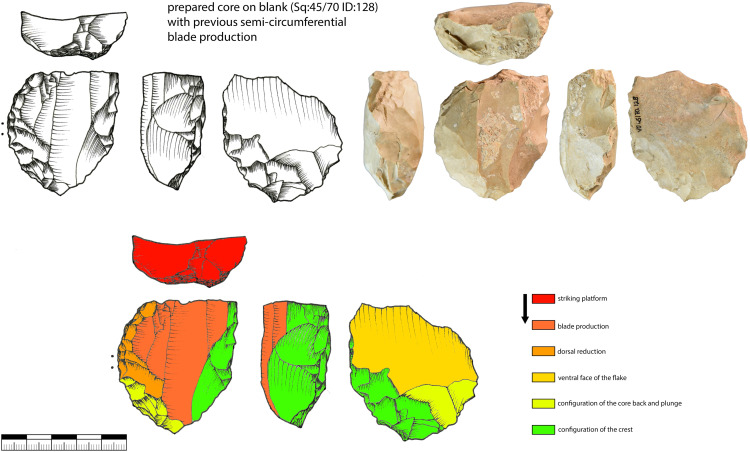
Vogelherd. Working stage analysis of a flake modified into a prepared core with a crest and a crested back (Photo & drawing: B. Schürch).

Reconstructing the configuration and the reduction of the large volumes (larger and around 100 mm) was difficult. Most of the cores preserved from this size class are either tested raw materials or multi-platform cores. For the cores with a large volume, we observed that natural convexities were used to initiate the reduction. This preserves volume for the reduction. However, for mid-sized and smaller volumes we could reconstruct that they were often fully configured and prepared. This is also confirmed by the size distribution of crested blades (Table 19). Besides the information directly preserved on the cores, we recorded crested blades (n = 176) and core tablets (n = 59) in AH IV-V that are either part of the initial configuration or the reconfiguration of the cores. Preparation of a crest was one of the most used methods of configuration. Negatives of cresting were visible on 36 cores. This crest was either installed on the narrow side of the core or between the narrow and the wide face. The refits and the fully configured cores also reveal that the core back is often carefully configured by a second crest or a crested back/side. This second crest on the back or the side of the core is, however, not intended for reduction, because the angle between the striking and reduction surface is not managed to be under 90°. This crested core back or side seems to be useful for managing the convexities on the core front (reduction face), comparable to a Levallois core, and possibly for handling the core. It can also be used to control the convexity on the back of the core to enable the asymmetrical core reduction. The best example for this core configuration comes from the backdirt (Fig 16). This piece is a large flake that removed large parts of the reduction face of a semi-circumferential blade core. This flake is then re-configured into a core again using the already existing convexities and preparing a crested back, the core plunge and a crest. The striking surface can be either plain or faceted, however faceted is much more common.

**Table 19:** Lithic raw materials and shells of IV and V of Vogelherd and their implications for land use in the Aurignacian.

Lithic raw material	Distance	n	%	Material	Sources	Method
**Jurassic chert**	5-15 and >15km	4692	82,17%	Jurassic chert	This paper; Burkert 2001; Burkert & Floss 2005	Macroscopic
**Tabular Jurassic chert**	≈ 130 km	185	3,24%	Tabular Jurassic chert (JH1a; JH1q; JH1h; JH1l; JH2c, refit 123)	This paper, Schürch et al. 2022	Macroscopic + FTIR
**Local river gravels**	≈ 200m-5 km	491	8,60%	Limestone; Siliceous limestone; Keuper chert; river gravels	This paper; Burkert 2001; Burkert & Floss 2005	Macroscopic
**Danube river gravels**	> 8 km	228	3,99%	Radiolarite; Quartz; Quartzite	This paper; Burkert 2001; Burkert & Floss 2005	Macroscopic
**Tertiary chert**	≈ 50 km	21	0,37%	Tertiary chert, Randecker Maar	This paper, Schürch et al. 2022	Macroscopic + FTIR
**Muschelkalk chert**	≈ 80 km	11	0,19%	Muschelkalk chert (Middle Triassic chert)	This paper; Burkert 2001; Burkert & Floss 2005	Macroscopic
**Total of 5710**		5628	98,56%			
**Shells**	**Distance**	**n**	**%**	**Genus**	**Source**	**Method**
**Glycymeris fossil Mainz basin**	≈200 km	3	100	Glycymeris	Schürch et al. 2021 & 2023	Malacological

The crested back and the crest to initiate the reduction, together with the configuration negatives on the front and back, show parallels to the configuration of a Levallois core [100]. The same parallel was identified in the initial Upper Paleolithic (IUP) [101,118]. Therefore, this specific configuration of mid to small sized cores at Vogelherd may be a feature of the early Upper Paleolithic. Other singular examples with a crested back or side from the Swabian Jura are also present in Geißenklösterle AH III [12, Fig 15] and Hohle Fels AH IV [13] but have not been discussed in detail before. This characteristic seems to be most pronounced at Vogelherd and might be explained by the intensity of raw material use.

**Fig 17:**
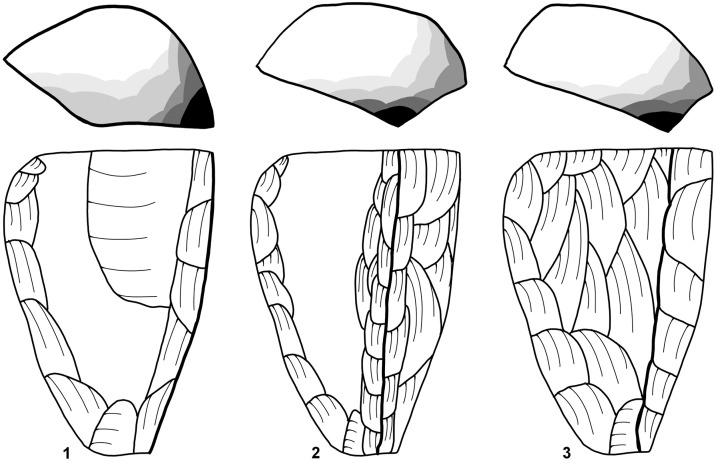
Different variants of prepared cores. Configuration of crested back/side and core plunge can occur for all variants: 1. Crest is prepared on the lateral of the core. 2. Crest is prepared between the narrow and wide surface of the core. 3. Convexity is prepared between the narrow and wide surface of the core. Top view shows the hypothetical reduction of the cores.

**Fig 18:**
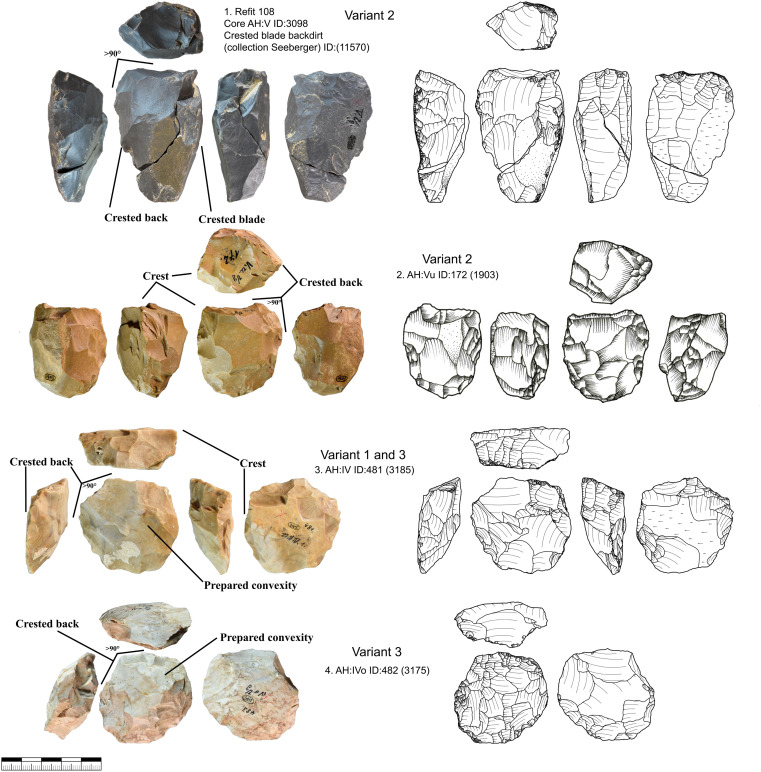
Prepared cores with a prepared crest and a prepared crested back. Crested backs are marked with the > 90° angle to show that they cannot be used to initialize reduction. Crests and prepared convexities are marked to show where the reduction was attempted to initialize or was initialized. For 1 the crested overshot blade stopped the reduction; for 2 the crest could not bring the reduction angle under 90°; for 3 and 4 hinges stopped the reduction (Photo & drawing: B. Schürch).

We have identified three different variations of core configuration. These have a preparation of a crest in common to start the reduction. All these variants can have a crested back/side and a prepared core plunge (Fig 17). They can all lead to a very similar semi-circumferential or asymmetrical reduction and are therefore only differentiated if the reduction failed at the beginning of the sequence. For variant 1 a crest is prepared between the core front and back. For variant 2 the crest is prepared between the narrow side and the wide face of the core front. For variant 3 a convexity is prepared between the narrow and wide face, similar to the preparation of a preferential Levallois core. However, the platform preparation is distinct from Levallois cores. Variants 2 and 3 are likely the result of problems in managing the convexity between the narrow and wide surface of the cores, that lead to hinges between the faces and made a reconfiguration necessary. For Geißenklösterle Hahn reconstructed variant 1 to be the most common [12]. For Hohle Fels IV Bataille reconstructed a variant comparable to variant 3, but without extensive preparation of the crested back and core front/back [13, Fig 24]. From Vogelherd we know several examples of the three variants (Fig 18).

#### Re-configuration and discard of cores.

**Fig 19:**
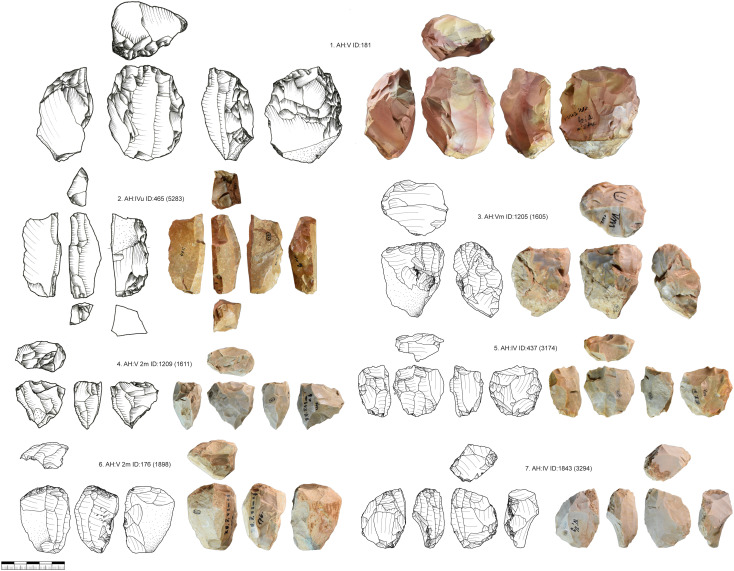
Vogelherd. Cores with striking errors. 1. Wide-faced flat core with crested back. After the reduction of two blades, the next removals could not remove the hinges between the wide and narrow surface of the core. 2. Narrow-sided core on large flake with crested back. After a hinge the core was turned 180°, but the hinge could not be removed from the new platform. 3. Semi-circumferential core, with crest between the narrow and wide surface. Removals could not remove hinges between narrow and wide surface. Several incipient cracks on the wide face show that the crest could not be re-prepared. 4. Semi-circumferential core with two hinges between narrow and wide face. 5. Semi-circumferential core with crested back, after a hinge between the narrow and wide face the core was turned 180°, but the hinge could not be removed. 6. Narrow-sided core, after several hinges between the wide and narrow surface, it was tried to re-configure the crest between the narrow-surface and the core back. Several incipient cracks near the crest show that this was not successful. 7. Semi-circumferential core with crested back. After the semi-circumferential reduction, a new crest was configured. This was not successful. 1-7 Jurassic chert (Photos & drawings: B. Schürch).

One central question is whether the four core types (semi-circumferential, narrow-sided, wide-faced flat and multi-platform cores) represent different reduction concepts or different stages of the same concept. The best information on this can be provided by discarded cores, when mismanagement of convexities or hinges stopped the reduction. The striking errors are visible in most of the four categories (Fig 19). The prepared cores (Fig 18) also display the same striking errors. Depending on the reduction intensity these striking errors have different characteristics. For the highly reduced cores, especially the ones reduced to the absolute maximum (see minimal raw material units) are almost cubic or cuboid and cannot be easily sorted into a core category. If striking errors occur earlier the core can be assigned to a core type.

The trend we want to make visible with this approach is the following: either cores are utilized to the absolute maximum (see minimal raw material units), or the cores had to be discarded due to failures in core management.

**Fig 20:**
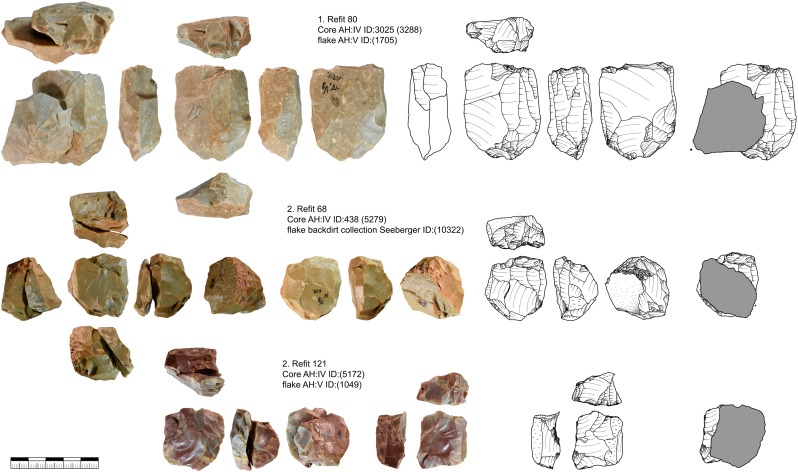
Vogelherd. Refits that show the preparation of the reduction face by the removal of a large flake. 1. Jurassic chert, 2-3. Radiolarite (Photos & drawings: B. Schürch).

**Fig 21:**
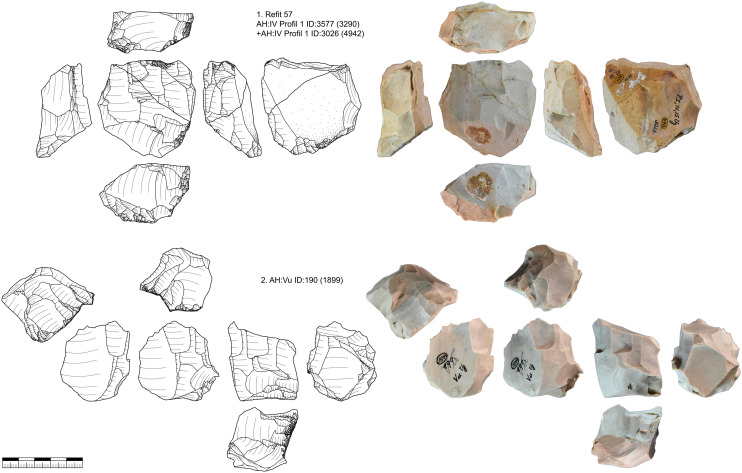
Vogelherd. Multi-platform cores: 1. Refitted core. After a semi-circumferential reduction the core was turned 90° and a second reduction surface was utilized at the core plunge. 2. After a semi-circumferential reduction, the core was turned 90° and the former core back was utilized to produce small blades and bladelets. 1-2. Jurassic chert (Photos & drawings: **B.** Schürch).

We selected several examples, where we could reconstruct a possible reason for discard and an effort was made to re-start the reduction. This should not imply that no blades or bladelets were removed from these cores before. Mismanagement or striking errors usually occur between the narrow and wide face of the cores, as this transitional part can only be re-configured with great difficulty. To avoid this mismanagement during reduction the following preparation steps were conducted:

- Neo crests: At the edge of the narrow surface, there is often an angle that allows the convexity to be restored at the same edge. Neo crests can also be prepared between the narrow and wide face. (Figs 18 and 19)
- Managing the convexity of the reduction face: From the crested back/side reaching onto the wide face (Fig 20), flakes can be removed to control the convexity around the transition between the narrow and wide face. These removals can remove the former reduction surface. (Figs 18–20)
- Removal of core tablets: With the removal of core tablets, it is possible to correct the reduction angle and to remove hinges that are close to the platform (Figs 3–5 and 23).
- Second platform: Creating a second platform at the core plunge/base with the intention to remove the hinges or overhanging convexity (Fig 18).
- Rotation: Abandoning the reduction surface, rotating the core, and utilizing a second surface to restart reduction. This results in multi-platform cores (Fig 21).

However, at the transition between the narrow and wide face, it is often only possible to remove a core tablet to restore the convexity, or a second striking surface needs to be set up at the base of the core to restore the convexity from the opposite direction. This second striking surface is most often used for the restoration of convexity and only in few cases used for extensive bidirectional blade or bladelet production. Most of the bidirectional cores in the assemblage are in fact unidirectional cores with a second platform at the core plunge, that aimed to remove hinges and correct convexities of the first reduction.

For multi-platform cores the rotation and creation of a new reduction surface are a possibility to continue using the existing volume even if the convexity of the original reduction surface could no longer be maintained. The described approaches can be reconstructed on the Vogelherd assemblage, both directly on the cores and with the help of refits.

#### Core generations and continuous decrease in size.

**Fig 22:**
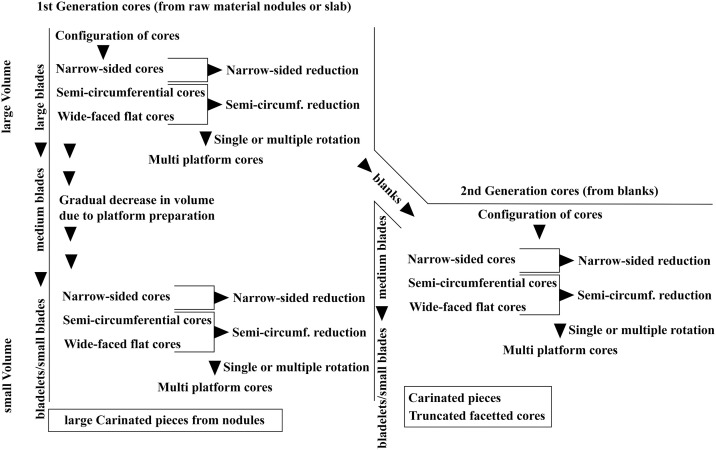
The two generations recognizable at Vogelherd. Left side: First Generation of cores often showing a gradual decrease in size of the cores. Right side: Second Generation of cores, here carinated pieces and truncated-faceted cores are more common.

At Vogelherd, we have identified various characteristics of the reduction system. These include the presence of a first and second generation of reduction (Figs 21 and 22). The first generation (n = 215) refers to cores produced from nodules or slabs. The second generation (n = 185) refers to cores that were produced from blanks of the first generation. These are largely but not exclusively carinated pieces and truncated-faceted cores. In Middle Paleolithic assemblages this is often termed ramification [3,79,119–121]. This ramification of the reduction process at Vogelherd seems to be very pronounced. In addition, we were able to determine that there is a continuous decrease of the size of the cores during the reduction (Fig 22). This is due to the repeated preparation of the striking surfaces through the removal of core tablets. In other words, there can be a continuum from blades to bladelet cores. We also found that some cores are configured in a very elaborate manner before removal can begin (see above). Various fully configured cores from the first generation testify to this. This is not limited to first generation cores only. Another observation is that narrow-sided, semi-circumferential and wide faced cores can be considered different stages in a reduction, sometimes visible in working stage analysis (see supplementary, S1–S9 Figs). This has recently been demonstrated experimentally [99]. The different core types can, therefore, be interpreted as different reduction steps of one reduction strategy. This is also visible sometimes directly at the cores (Fig 19).

#### Separated blade and bladelet production?

**Fig 23:**
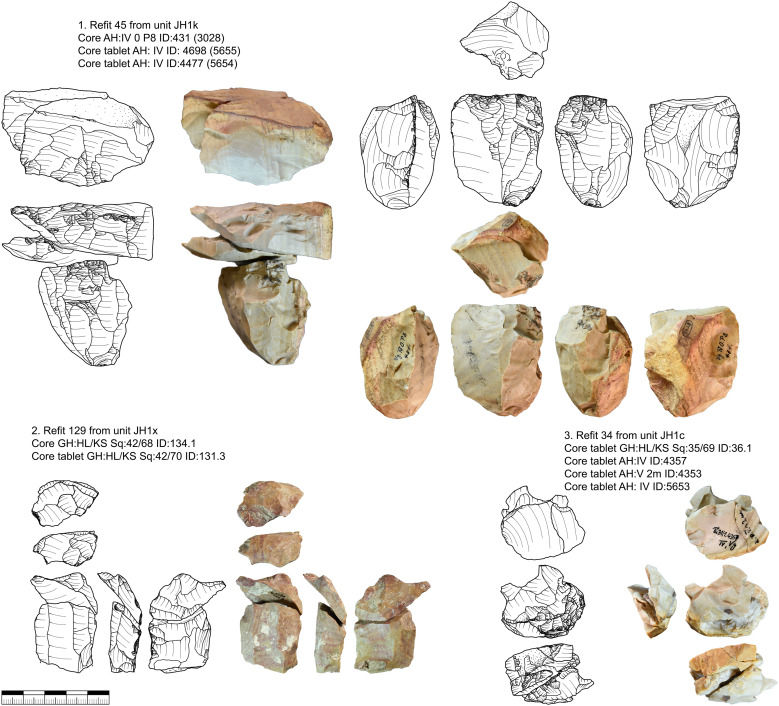
Vogelherd. Cores with platform configuration. 1. Refit 45: Semi-circumferential bladelet/blade core with crested back and negative of a partial removal/configuration of the reduction face, refitted with two core tablets. 2. Refit 129: Semi-circumferential bladelet core with refitted core tablet. 3. Refit 34: Four refitted core tablets. 1, 2. Jurassic chert; 3. Brown Jurassic chert (Photos & drawings: B. Schürch).

**Fig 24:**
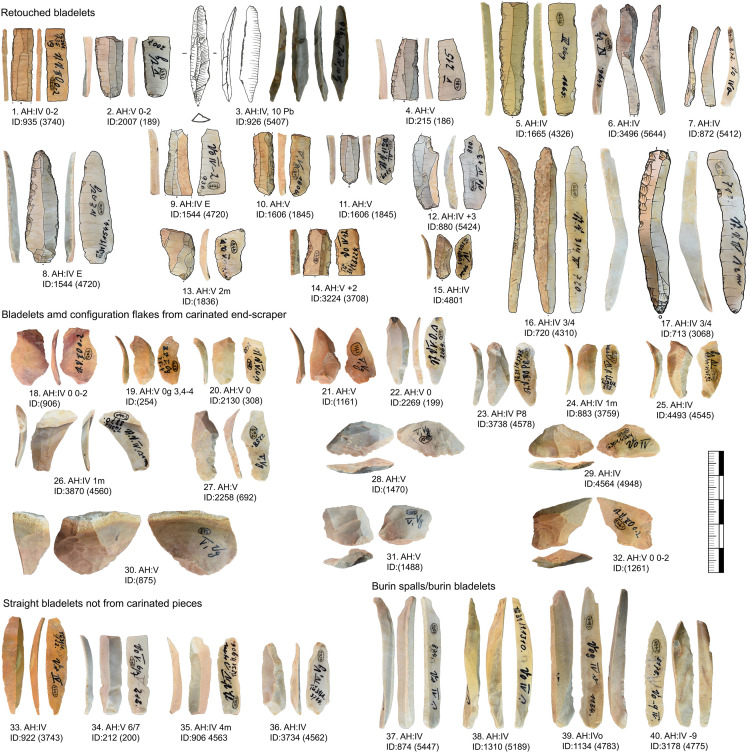
Vogelherd. Bladelets. 1-3 Dufour bladelets; 4-5, 9-15 retouched bladelets; 6-7, 16-17 retouched burin spalls; 8 Font-Yves bladelets; 18-27 bladelets from carinated end-scrapers; 29-32 configuration flakes from carinated end-scrapers; 33-36 straight bladelets from non-carinated pieces; 37-40 burin spalls. 2-17, 22-23, 31, 34-40 Jurassic chert; 1, 18-21, 24-25, 33 brown Jurassic chert (Photos: B. Schürch; Drawings: 1-2, 4-40. B. Schürch; 3 modified after Hahn 1977).

More than one possibility to produce bladelets is observed in the Vogelherd assemblage: we could reconstruct that blade and bladelet production is not separated from each other in Vogelherd. This means blade and bladelets can be produced in one reduction cycle or on the same cores (Figs 22–24).

1. **Carinated cores:** Carinated end-scrapers and carinated burins are the most common way to produce bladelets in the assemblage. Most of the time flakes or thick blades are used as blanks for these cores.
2. **Continuous reduction from blade to bladelet cores**: We can directly reconstruct this by means of refits. These refits show the reduction of core tablet, which leads to a reduction in length. In a few extreme cases, these cores even could be misinterpreted as flake cores due to the reduction of the striking surface.
3. **Different products depending on the convexity of the reduction face**: We note this for the semi-circumferential cores. Here sometimes bladelets and blades were produced on the same cores. The bladelets were often reduced on the narrow side and blades on the wide side. For Geißenklösterle Teyssandier defined this as intermediate bladelet production [29]
4. **Non-carinated bladelet cores**: These bladelet cores show the same variety of cores as the rest, i.e., semi-circumferential, narrow-sided, wide-faced flat and multi-platform cores. Most often it is found for fine-grained (or high quality) raw materials. It is possible that the cores were reduced more intensively, in other words that they shrank (point 2 of this list), or that smaller raw material aggregates were utilized. This is evident in the fine-grained raw material units JH1a and JH1b (JH1a and b are not considered minimal raw material units), which have produced many small cores, non-carinated as well as carinated and truncated-faceted cores.
5. **Combination of non-carinated and carinated cores:** There are few cases where regular non-carinated cores are used to produce bladelets from the core base or back.
6. **Truncated-faceted cores:** These cores are most often configured on flakes or blades just like the carinated cores. Often these are combined with a carinated end-scraper or a carinated burin.
7. **Burins:** Burins are another way to produce bladelets/burin spalls. In the assemblage there are a variety of different burin types. For this analysis we only counted the very large burins into the core category. These show lots of similarities to narrow-sided cores. For the other burins that are listed under the tool category a function as core is possible as well. However, this needs to be studied in more detail. From the assemblage we also could recover some retouched burin bladelets, that hint towards a core function of burins.

The continuous production of blade and bladelets due to the decrease in size of the cores is often described as a characteristic of the Protoaurignacian [23]. Our observations show, however, that this is also an evident feature in the Aurignacian of Vogelherd (41.000–35.000 cal BP) that is linked to intense raw material use and not to cultural or technological differences between the Proto- and Early Aurignacian. The bladelets from Vogelherd (n = 193 in IV and V) are underrepresented in the assemblage. In total there are 15 modified bladelets in AH IV and V. These are mostly bladelets retouched dorsally on one edge. There are three Dufour bladelets [14,122] in the assemblage and one Font-Yves bladelet. Two burin bladelets show a modification of the tip. The other bladelets are without formal modifications. The analysis of some bladelets, refitted with carinated piece showed, that formal modifications are not always present on bladelets utilized as tools [82]. Even if bladelets are underrepresented in the assemblage, the typical Aurignacian range of variation is present in the assemblage. Twisted bladelets, lateral preparation from carinated pieces, straight bladelets and burin bladelets are present in the assemblage as well.

#### Narrow sided, burin like cores, carinated burins or burins?

**Fig 25:**
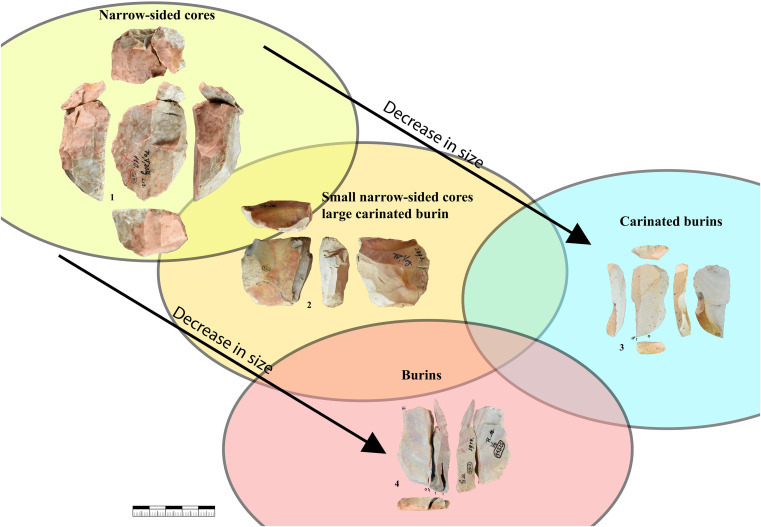
Overlap of different burin types and narrow-sided cores depending on definition and the functional aspect. 1. Narrow sided core with a carinated end on the core base (refit 103; core AH:V 113 (1620); flake: backdirt collection Seeberger ID:(11147), 2. Small narrow-sided core (AH:IV ID:2893 (3231)), 3. Busked burin (GH:HL/KS Sq:65/62 ID:24), 4. Truncated burin with two refitted burin spalls (refit 27; burin AH:unknown ID:(11525); burin spall AH:IV −2 ID:3914 (5413); burin spall AH:IV ID:865 (5409). 1-4 Jurassic chert (Photos & drawings: B. Schürch).

When we analyze the cores that are mostly reduced on a narrow face, we run into the same problem we are facing for carinated pieces and end-scrapers: When are we dealing with cores and when are we dealing with tools? It also is not always unambiguous to separate these types (Fig 25). Bataille & Conard pointed out for the Hohle Fels IV assemblage that the burins could also be considered as cores [13]. Depending on the definition researchers use (typological, technological, separation by shapes and size of produced bladelets/blades) these types can overlap. To get a better understanding of this bladelet production, only use-wear studies could really help to clarify which prerequisites burins or burin spalls need to have in order to be used. We discussed this question for carinated end-scrapers and end-scraper [82]. In this analysis we also included carinated burins. None of them showed traces of use around the reduction surface of the bladelets. Another analysis of the burin and burin spalls would be needed to understand the role of burins and how to separate them from burin like cores and small narrow-sided cores.

#### Flake cores in the Aurignacian?

The reduction sequence at Vogelherd is oriented towards the production of bladelets and blades. However, flakes are used regularly as tools in the assemblage (n = 292; 11,52% of all tools). Of the 18 flake cores few can be explained by mixing with Middle Paleolithic (Levallois cores, Quina cores). The remaining cores are most of the time made from limestone or are very intensely reduced cores (Fig 12; or raw material unit JH1d). For the very intensely reduced cores, the recurrent configuration of the platform leads to the impression that cores were mainly used for flake production [65]. Therefore, we cannot confirm results that implied flake production is targeted at Vogelherd [69]. The flakes produced at Vogelherd are by-products or result from core configuration and they get occasionally used as tools or bladelet cores. At other Aurignacian sites, however the production of flakes is more pronounced and targeted than at Vogelherd [14,123].

### Economic decisions and their influence on the *chaîne opératoire*—Indication for the reconstruction of site function

**Fig 26:**
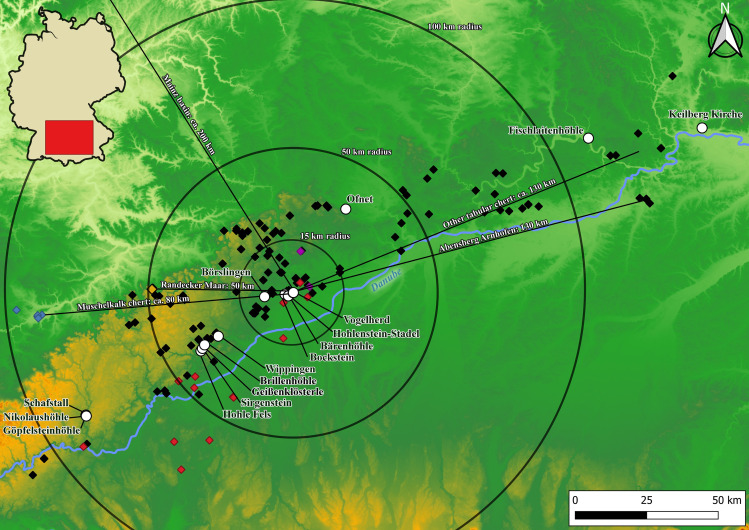
Map of Southern Germany. Aurignacian sites and the long-distance raw material connections of Vogelherd (raw materials: black = Jurassic chert; red = Radiolarite and other river gravels; purple = Keuper chert from river gravels; orange = Tertiary chert from the Randecker Maar; blue = Muschelkalk chert from the Neckar; outcrop data from: this paper; Burkert 2001; Schürch et al. 2022; basemap: © European Union, Copernicus Land Monitoring Service 2020, European Environment Agency (EEA)“, f.ex. in 2018: “© European Union, Copernicus Land Monitoring Service 2018, European Environment Agency (EEA)” with funding by the European Union).

Binford build a baseline for the reconstruction of lithic raw material procurement [124–128]. The concept of embedded procurement of lithic raw material is often applied in Paleolithic studies. For the Aurignacian of Vogelherd we have different sources for the reconstruction of raw material use. The first source is our macroscopic determination of the used raw material, which is not ideal because they cannot be empirically tested. These are supplemented by empirical studies on the raw material sourcing using IR-spectroscopy [106]. The second source are raw material units that show the intensity of use on site and in some cases point towards specific raw material sources. The third source is raw material quality. In most cases we have good to medium quality raw material (material that is fine to coarse grained, with none or few inclusions) used at the site. Low quality raw material is also used at the site. These raw materials are limestone, Keuper chert, fissured radiolarite and only partially silicified Jurassic chert. By bringing together all of these, we can try to reconstruct land use patterns. Table 19 and Fig 26. shows the different raw materials used at the site, if they could be assigned to an outcrop, geologic source, or a local range of the foragers (Fig 26).

**Fig 27:**
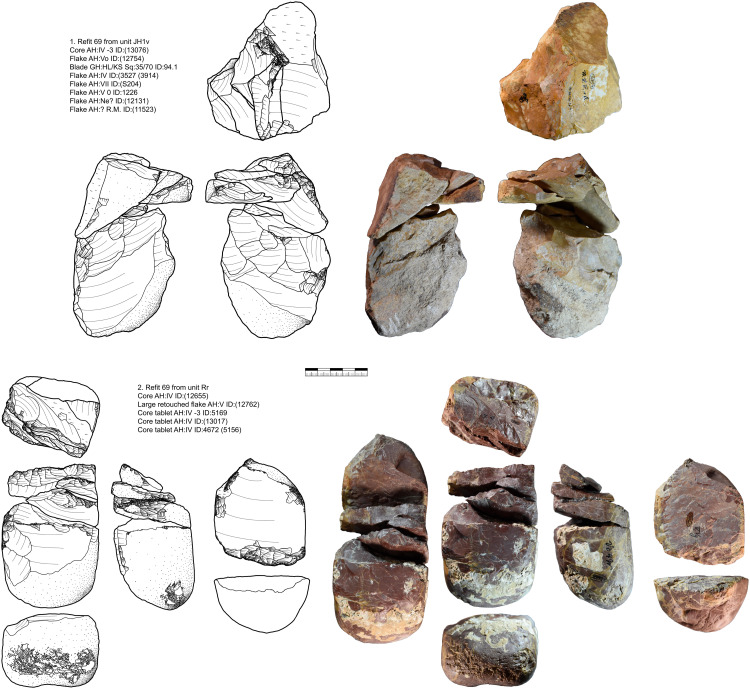
Vogelherd. Refit sequences of two cores of low-quality raw material. 1 Brown Jurassic chert, 2 Radiolarite (Photos & drawings: B. Schürch).

For specific raw materials (raw material units) we have already shown the intensity of reduction. Although it is hard to classify this, a trend emerges that separates the high and medium quality materials from the low-quality materials. For the minimal raw material units that are of good quality (JH1d, JH1w, JH1m) we could show that they were intensely reduced to the maximum. Tools from these units were produced, hafted, used, resharpened and discarded on site. These units but also others not presented in detail, show a pattern of intense use of the site, pointing towards a longer lasting use of the site and not only short stopovers. For the tabular Jurassic chert that is a raw material transported roughly 130 km, especially visible in unit JH1a, the same trend emerges. Also, this very fine-grained material was used and reduced to the maximum. For the raw materials from local river gravels and the river gravels of the Danube, another pattern emerges. These raw materials are often of poor quality. They were brought to the site and reduction of this material produced almost no formal tools [129]. This can be illustrated by two refits (Fig 27). The first is a brown Jurassic chert (Fig 27, 1) that is refitted from eight artifacts. This nodule is composed, for the most part, of poor quality and coarse grained chert. After the configuration of the platform the nodule was discarded on site due to the poor quality. The second is a radiolarite nodule (Fig 27, 2). This cobble is traversed by fissures. Also, here after the configuration of the platform, the core was discarded. In this case the nodule was used as a hammerstone and one large flake as a tool, comparable to a chopping tool. From this and other examples, we can deduce that low-quality raw materials were brought to the Vogelherd and only subsequently underwent extensive testing. The same applies for the limestone cores. The number of tools from these low-quality materials is very low, most tools from these materials are hammerstones.

From this pattern of use we can reconstruct that material suited for tool production was handled with great care and that it was used to the maximum extent. This is true for good quality local materials and also for raw materials that were brought to the site over large distances. The low-quality raw materials were brought to the site without testing them thoroughly and were often discarded in the initial stages of the reduction due to their quality. The use of these materials from the nearby river gravels might have been an adaption to the longer stays at the site [129].

These pattern points towards a longer lasting use of the site as a seasonal base camp [126,129]. Other evidence for this comes from organic artifacts. Especially ivory working, that is time consuming [39], was conducted at the site intensively. The whole *chaîne opératoire* of ivory working is present at the site with ivory tusks cached at the site, the reduction of ivory tusks into workable volumes, half-finished ivory rods, ivory rods, half-finished ivory beads, over 600 finished ivory beads (most of them double perforated), ivory figurines and ivory tools [21,39,42–44,49,130,131]. Also, for the organic points, mainly split-based, the whole *chaîne opératoire* is present at the site [41]. In total there are 60 split-base points and even more other indetermined points or preforms [41], more than in all other sites of the Swabian Jura combined. Further evidence can be added by the features that Riek recorded in AH IV and V. He recorded caches of mammoth ivory, organic projectiles and also tools (end-scapers and pointed blades) [21]. Especially the “*Mammutknochenhaufen*” (=mammoth bone pile) a structure build with mammoth bones and teeth containing a bundle of projectile points towards the caching of artifacts at the site for future use.

The evidence suggests that the site was used over longer periods of time and not only for stopovers. The lack of stratigraphical resolution and the fact that almost all sites in the Swabian Jura are palimpsests, makes it difficult to go in more detail. However, it is likely that the Aurignacian of the site represents a mixture of longer and shorter stays. By the patterns emerging from the different *chaînes opératoires* and the hunting activities at the site [114,132] we can conclude that Vogelherd was a central place in the Aurignacian. Niven’s analysis of the fauna showed that the site was mainly used during the late summer and fall, primarily reindeer and horse were exploited. For reindeer this correlates with their migration. The “[…] *people invested considerable time and labor to intensively exploit reindeer and horse for fat in addition to meat protein in a consistent fashion over many millennia at the site*” [132]. This again fits well with the longer stays reconstructed by the lithic *chaîne opératoire* and raw material usage*.* The site use was embedded in an annual recurring use of the cave in late summer and fall with longer lasting stays at the site, maybe over several months. Leaving back damaged hunting gear, intensely used lithic artifacts, while also caching ivory, organic point and other gear for the use in the next season. In the future we hope that Niven’s analysis [114,132] can be supplemented by faunal analysis from the backdirt providing new insights of hunting of small game, which is underrepresented in the 1931 excavation due to size selection [48,133,134].

The raw materials also allow a reconstruction for land use. This points towards the use of an area spanning from the Neckar to the Altmühl valley covering an area of roughly 180 km from West to East. The only evidence pointing further northwards are fossil Glycymeris shells (AH V) originating from the Mainz basin [52,135]. None of the Glycymeris shells from AH V are perforated. The only two perforated specimens come from the backdirt of the site. It is therefore possible that these shells are not exclusively used as ornaments but were cached for future use or may have had different functions, e.g., they possibly could have served as containers [135].

### Vogelherd and Geißenklösterle core reduction strategies

Both Vogelherd and Geißenklösterle show large overlap in their reduction strategies. Mostly narrow sided, semi-circumferential and carinated reduction are dominant at both sites. For the cores at Vogelherd and to a lesser extend at Geißenklösterle, we could also record the transition between the different core categories (narrow-sided, semi-circumferential and wide faced flat). That is why we agree with Hahn’s statement that the reduction strategy can be described as opportunistic [12,35]. During the reduction process the core morphology can change, and the reduction was adapted to the convexities of the core. Another term to express this is the term ramification. Therefore, the opportunistic behavior described by Hahn could be described a highly ramified [78] reduction system.

The presence of truncated-faceted cores in both sites is worth mentioning. At Geißenklösterle these truncated-faceted cores are more common in the upper Aurignacian (Fig 15, 4-5) [12,28,29,34]. In Vogelherd, we can observe the same trend of a presence in the upper Aurignacian, but it is not that pronounced. The presence of over 30 truncated core ends in Vogelherd emphasizes this form of bladelet production as a feature of the Aurignacian in the Swabian Jura.

Carinated pieces in Vogelherd are more abundant in the upper Aurignacian but they are also present in the lower Aurignacian. At Geißenklösterle both Hahn and Teyssandier observed more carinated and nosed end-scraper in the lower Aurignacian [12,29]. Carinated burins or busked burins are rare in Geißenklösterle. In Vogelherd they are more abundant in the upper Aurignacian. For the carinated pieces the ratios are not comparable because different definitions for carinated artifacts were used. As we used the 12 mm threshold to separate cores (carinated pieces) from tools at Vogelherd and Hahn used a typological definition, both datasets of carinated pieces are subject to different selection criteria. The minimum thickness of the carinated scraper heads at Geißenklösterle is 15,6 mm for carinated end-scrapers. For thick nosed end-scrapers the minimum thickness is 12,1 mm. This is not much different than Vogelherd but applying the 12 mm threshold at Geißenklösterle could change the number of carinated pieces especially in the upper Aurignacian were the carinated pieces are absent using the current definition.

The number of bladelets of the two assemblages cannot be compared due to the sampling strategy of Riek where no or only rarely smaller artifacts were sampled, and no sieving took place. At Vogelherd only 193 bladelets were recovered in 1931. At Geißenklösterle, the analysis of lithics retrieved from water screening is currently ongoing and will yield reliable numbers for bladelets. Retouched bladelets are generally rare and typical Aurignacian types like Dufour or Font-Yves bladelets are almost absent in both assemblages. At Vogelherd we could recover three Dufour bladelets (IV = 2; V = 1) and one Font-Yves point (Krems point) from AH IV. At Geißenklösterle Dufour bladelets are rare as well and only one was reported by Hahn [12] and also Teyssandier [29], whereas Moreau reported three [40,136]. These retouched forms therefore do not seem to have a significant role in both assemblages.

The spectrum of formal tools of both sites overlaps with pointed blades, splintered pieces, burins, end-scrapers and laterally retouched blanks. This is not only true for the lithic but also concerning the organic artifacts, music instruments, personal ornaments and figurative art [38,39,74].

In general, the cores, not including truncated-faceted cores and carinated cores, are significantly larger at Geißenklösterle than at Vogelherd (see results and S3 Table). At Geißenklösterle the median of the length of the reduction face is 52 mm (mean = 52,21 mm) and at Vogelherd its 41,75 mm (mean = 45,18 mm). The same trend is present for the weight of the cores, at Geißenklösterle the median is 76,5 g (mean = 111,94g) and at Vogelherd 48,30 g (mean = 122,32g). The mean at Vogelherd is heavily influenced by the presence of several large but low-quality raw materials tested but not intensely reduced. The trend of smaller or more intensely reduced cores at Vogelherd is also supported by the refits and minimal raw material units. The crested backing and the continuous reduction from blade to bladelet cores is documented more often in Vogelherd than at Geißenklösterle. However, both are still present at Geißenklösterle. The presence of refitted sequences at both sites allow to reconstruct on site knapping at Vogelherd and Geißenklösterle. At Geißenklösterle this information can be also localized in the cave and knapping zones can be identified. At Vogelherd this spatial information is missing. However, Riek also recorded several knapping areas, but without further detailed descriptions [21]. The amount of lithic production on site is also a factor that needs to be accounted for when comparing the sites. While at Vogelherd the upper Aurignacian (AH IV) is roughly twice as rich as the lower Aurignacian (AH V), it is reversed at Geißenklösterle where the lower Aurignacian (AH III) is roughly twice as rich as the upper Aurignacian (AH II). This can be linked to more intense on-site knapping in AH III of Geißenklösterle also visible from the complete chains of production while the production chain in AH II is incomplete with more diverse raw materials [9,29].

To summarize, both lithic assemblages and the reduction strategies at both sites are similar, with few clear temporal trends. Differences in the assemblages may be best explained by differences in site use. For Geißenklösterle the site was mainly used in winter and spring focusing on wild horse, reindeer and mammoth as the main prey and a high presence of cave bears [137]. The stays at the site either had a longer duration or the people repeatedly visited the cave over winter and spring [137]. To the contrary at Vogelherd the site was mainly used during late summer and fall, and wild horse and reindeer were the main prey [132]. We also reconstructed longer stays at Vogelherd with the caching of artifacts for future stays at the site. A use cycle of one group that utilized both sites would be possible but could only be proven by direct refits. Although seasonal trends of site use can be reconstructed, they do not exclude occasional use of the site during other seasons with various activities. It must also be highlighted that there are many other sites that played an important role for subsistence in the Aurignacian. In addition, the role for Aurignacian subsistence of the poorly preserved open-air sites in the region is unclear [138–141].

Concluding the comparisons of the sites it seems that the typo-technological expressions of the Aurignacian in the Swabian Jura is relatively stable with opportunistic core reduction to produce blades and larger bladelets as well as a diverse array to produce bladelets on carinated pieces, truncated-faceted cores and burin cores. This could be explained by the fact that either the basal Aurignacian adaption [35,142] is relatively stable over the Aurignacian or that the regional adaption [142,143] of the subsistence economy is the same for both sites. The palimpsest at Vogelherd makes it difficult to evaluate the role of chronology in the expression of the Aurignacian assemblage. This also makes it difficult to discuss in a larger framework like with the French Aurignacian sequence. The relatively few retouched bladelets in the assemblages of the Swabian Aurignacian, could, however, be addressed as a general difference between both regional expressions.

## Conclusion

The technological study of the Vogelherd assemblages is the most comprehensive analysis of this assemblage excavated more than 90 years ago. This research focused on the reduction sequences and contextualized them studying all 5,710 lithic artifacts from layer IV and V of the site. Using these assemblages, we could reveal the limitations of core typology and enable a more comprehensive understanding of the reduction processes in the Swabian Aurignacian. The typological definition of many cores is a result of the opportunistic reduction and re-configuration process and does not necessarily represent distinct reduction concepts. We therefore highlight the significance of refitting and sorting of minimal raw materials like J. Hahn also emphasized at Geißenklösterle [12]. Our work also reveals the complexity of the Aurignacian bladelet production. Carinated pieces play a central role for the bladelet production but do not reflect the full variability. We could also link the reduction sequences with the raw material economy. The reduction sequences are influenced by the quality of the lithic raw materials and the distance from the source. This shows advanced planning of raw material needs adapted to the individual situation.

Combining our new results with previous analyses on Vogelherd [15,39,41,114,132], a complex picture of site-use during the Aurignacian emerges. These indicate long and intense occupations of the site. The raw material procurement demonstrates the important role of the site in a far-reaching land use pattern with repeated occupations. These results may be supplemented in the future by functional analyses of the tools, which could then better reveal which and how intense different types of activities were carried out at the site. A more detailed analysis of the bladelets is needed in the future, studying whether this important class of artifacts was used as tools without formal modification. Combining the results from Vogelherd and Geißenklösterle allows to identify characteristics of the Swabian Aurignacian. These include opportunistic reduction sequences, a gradual decrease in size of the cores from blade to bladelet cores, the presence of truncated-faceted cores, and the low number of modified microliths. With these new findings, we hope to gain more in-depth insights into the Aurignacian occupation of the Swabian Jura in the future by combining them with the findings of other innovations of the early Upper Paleolithic, such as symbolic communication [58,144,145], in order to better understand the behavioral repertoire of early anatomically modern humans.

## Supporting information

S1 Table Detailed overview of tools. *The other 10 cores that are also under the tool category are Chopping tools that could have been exploited as flake core before.
(XLSX)

S2 Table Independent-Samples Kruskal-Wallis Test of the different core types and core attributes from Vogelherd. (XLSX)

S3 Table Independent-Samples Mann-Whitney U Test of the cores (not including carinated and truncated faceted cores) from Vogelherd and Geißenklösterle. (XLSX)

S4 Table Raw data of the Vogelherd core types and measurements. (XLSX)

S1 Fig Narrow-sided blade/bladelet core with two reduction faces (Photos and drawings: B. Schürch). (TIF)

S2 Fig Semi-circumferential bladelet core (Photos and drawings: B. Schürch). (TIF)

S3 Fig Multi-platform blade core with two reduction surfaces (Photos and drawings: B. Schürch). (TIF)

S4 Fig Wide-faced flat bladelet core (Photos and drawings: B. Schürch). (TIF)

S5 Fig Narrow-sided blade/bladelet core (Photos and drawings: B. Schürch). (TIF)

S6 Fig Narrow-sided blade/bladelet core with carinated core at the back (Photos and drawings: B. Schürch). (TIF)

S7 Fig Prepared core/blade core (Photos and drawings: B. Schürch). (TIF)

S8 Fig Prepared core (Photos and drawings: B. Schürch). (TIF)

S9 Fig Core discarded in transition from narrow-sided to a semi-circumferential core (Photos and drawings: B. Schürch). (TIF)

S10 Fig Artifacts from the raw material unit JH1d with refits 98, 113, 93 and 94 (Photos: B. Schürch). (TIF)

S11 Fig Artifacts from the raw material unit JH1d with refits 38 and 39 (Photos: B. Schürch). (TIF)

S12 Fig Artifacts from the raw material unit JH1d with refits 95, 127 and 92 (Photos: B. Schürch). (TIF)

S13 Fig Artifacts from the raw material unit JH1w with refits 138, 97 and 135 (Photos: B. Schürch). (TIF)

S14 Fig Artifacts from the raw material unit JH1w with refits 118 and 114 (Photos: B. Schürch). (TIF)

S15 Fig Artifacts from the raw material unit JH1w with refits 141 and 96 (Photos: B. Schürch). (TIF)

S16 Fig Artifacts from the raw material unit JH1m with refits 142, 112 and 90 (Photos: B. Schürch). (TIF)

S17 Fig Artifacts from the raw material unit JH1m with refits 111 and 58 (Photos: B. Schürch). (TIF)

S18 Fig Artifacts from the raw material unit JH1m with refit 91 (Photos and drawing: B. Schürch). (TIF)

## References

[1] Çep B. Das mittelpaläolithische Silexinventar des Bocksteins im Lonetal (Schwäbische Alb). Vielfalt der Formen oder Fortbestand einer technologischen Idee? Beiträge zur Ur- u Frühgesch Mitteleuropas 75, Varia neolithica VIII. 2014:79–92.

[2] Çep B. Das Mittelpaläolithikum auf der Schwäbischen Alb. In: Baales M, Pasda C, editors. “All der holden Hügel ist keiner mir fremd” Festschrift zum 65 Geburtstag von Claus-Joachim Kind. Universitätsforschungen zur prähistorischen Archäologie. Bonn: Verlag Dr. Rudolf Habelt; 2019. p. 99–107.

[3] Çep B, Schürch B, Münzel SC, Frick JA. Adaptive capacity and flexibility of the Neanderthals at Heidenschmiede (Swabian Jura) with regard to core reduction strategies. PLoS One. 2021;16(9):e0257041. doi: 10.1371/journal.pone.0257041 34492092 PMC8423277

[4] Frick JA, Schürch B, Çep B. The Middle Paleolithic at Große Grotte (Blaubeuren, southern Germany). New insights from lithic technology and implication for assemblage classification. J Paleolithic Archaeol. 2022.

[5] Conard NJ, Schmid VC, Bolus M, Will M. Lithic assemblages from the Middle Paleolithic of Geißenklösterle Cave provide insights on Neanderthal behavior in the Swabian Jura. Quartär. 2019;66:51–80. doi: 10.7485/QU66_3

[6] Conard NJ, Janas A, Marcazzan D, Miller CE, Richard M, Schürch B, et al. The cultural and chronostratigraphic context of a New Leaf Point from Hohle Fels cave in the Ach Valley of southwestern Germany. Mitteilungen der Gesellschaft für Urgeschichte. 2021;30:41–66.

[7] Wetzel R, Bosinski G. Die Bocksteinschmiede in Lonetal (Markung Rammingen, Kreis Ulm). Teil I: Text. 1st. ed. Bosinski G, editor. Stuttgart: Müller & Graff; 1969. p. 230.

[8] Sesselfelsgrotte RJ III. Der G-Schichten-Komplex der Sesselfelsgrotte. Zum Verständnis des Micoquien. Freund G, Reisch L, editors. Saarbrücken: Saarbrückener Druckerei und Verlag; 1997. p. 472.

[9] Conard NJ, Bolus M. Radiocarbon dating the appearance of modern humans and timing of cultural innovations in Europe: new results and new challenges. J Hum Evol. 2003;44(3):331–71. doi: 10.1016/s0047-2484(02)00202-6 12657520

[10] Conard NJ, Bolus M. Radiocarbon dating the late Middle Paleolithic and the Aurignacian of the Swabian Jura. J Hum Evol. 2008;55(5):886–97. doi: 10.1016/j.jhevol.2008.08.006 18926559

[11] Hahn J, Owen LR. Blade technology in the Aurignacian and Gravettian of Geissenklösterle Cave, Southwest Germany. World Archaeol. 1985;17(1):61–75. doi: 10.1080/00438243.1985.9979950

[12] Hahn J. Die Geißenklösterle-Höhle im Achtal bei Blaubeuren 1 Fundhorizontbildung und Besiedlung im Mittelpaläolithikum und im Aurignacien. Stuttgart: Theiss; 1988. 262 p.

[13] Bataille G, Conard NJ. Blade and bladelet production at Hohle Fels Cave, AH IV in the Swabian Jura and its importance for characterizing the technological variability of the Aurignacian in Central Europe. PLoS One. 2018;13(4):e0194097. doi: 10.1371/journal.pone.0194097 29630601 PMC5891003

[14] Hahn J. Aurignacien, das ältere Jungpaläolithikum in Mittel- und Osteuropa. Köln [u.a.]: Böhlau; 1977. X, 355 p.

[15] Conard N, Niven L, Mueller K, Stuart A. The chronostratigraphy of the upper paleolithic deposits at Vogelherd. Mitteilungen der Gesellschaft für Urgeschichte. 2003;12:73–86.

[16] Adler DS, Prindiville TJ, Conard NJ. Patterns of spatial organization and land use during the Eemian interglacial in the Rhineland: New data from Wallertheim, Germany. Eurasian Prehistory. 2003;1:25–78.

[17] Schürch B, Conard NJ. Reassessing the cultural stratigraphy of Vogelherd Cave and the settlement history of the Lone Valley of SW Germany. Tübingen: European Society for the study of Human Evolution; 2022.

[18] Schürch B. Reassessing Vogelherd: Lithic technology, Raw materials and Stratigraphy: University Tübingen; unpublished Dissertation.

[19] Riek G. Paläolithische Station mit Tierplastiken und menschlichen Skelettresten bei Stetten ob Lontal. Germania. 1932;16:1–8.

[20] Riek G. Les civilisations paléolithiques du Vogelherd, près de Stetten-ob-Lonetal (Wurtemberg). Préhistoire. 1933;II(2):149–81.

[21] Riek G. Die Eiszeitjägerstation am Vogelherd im Lonetal. - Die Kulturen. Leipzig: Kabitzsch; 1934. VIII, 338 p.

[22] Conard NJ, Grootes PM, Smith FH. Unexpectedly recent dates for human remains from Vogelherd. Nature. 2004;430(6996):198–201. doi: 10.1038/nature02690 15241412

[23] Bon F. L’ Aurignacien entre mer et océan: réflexion sur l’unité des phases anciennes de l’Aurignacien dans le sud le la France. Paris: Société Préhistorique Française; 2002. 253 Seiten p.

[24] Hahn J. Das Aurignacien in Mittel- und Osteuropa [Hochschulschrift]. Köln; 1969.

[25] Falcucci A, Peresani M. Protoaurignacian Core Reduction Procedures: Blade and Bladelet Technologies at Fumane Cave. Lithic Technol. 2018;43(2):125–40. doi: 10.1080/01977261.2018.1439681

[26] le Brun-Ricalens F, Brou L. Burins carénés-nucléus à lamelles: identification d’une chaîne opératoire particulière à Thèmes (Yonne) et implications. Bulletin de la Société préhistorique française. 2003:67–83.

[27] Le Brun-Ricalens F, Bordes J-G, Sciences IUoPaP. Productions lamellaires attribuées à l’Aurignacien chaînes opératoires et perspectives technoculturelles; actes du XIVe congrès de l’UISPP, Université de Liège, 2-8 septembre 2001, Session 6 - paléolithique supérieur, Colloque C6.7. Luxembourg: Musée National d’Histoire et d’Art; 2005. 568 p.

[28] Teyssandier N. Les débuts de l’Aurignacien en Europe. Discussion à partir des sites de Geissenklösterle, Willendorf II, Krems-Hundssteig et Bacho Kiro. 2003.

[29] Teyssandier N. En route vers l’Ouest les débuts de l’Aurignacien en Europe. Oxford: Hedges; 2007. 312 p.

[30] Bataille G, Conard NJ, Bolus M, editors. A closer look at lithic variability in the Aurignacian of the Swabian Jura – the Hohle Fels example. Paris: UISPP; 2018.

[31] Bataille G, Conard NJ. Burin-core technology in Aurignacian horizons IIIa and IV of Hohle Fels Cave (Southwestern Germany). Quartär. 2021;65:7–49.

[32] Kind C-J, editor. Löwenmensch und mehr. Die Ausgrabungen 2008–2013 in den altsteinzeitlichen Schichten der Stadel-Höhle im Hohlenstein (Lonetal), Gemeinde Asselfingen, Alb-Donau-Kreis. Wiesbaden: Dr. Ludwig Reichert Verlag; 2019.

[33] Demars PY, Laurent P. Types d’outils lithiques du Paléolithique supérieur. CNRS, editor. Paris: CNRS; 1989.

[34] Hahn J. Erkennen und Bestimmen von Stein- und Knochenartefakten: Einführung in die Artefaktmorphologie. Tübingen: Verlag Archaeologica Venatoria: Institut für Urgeschichte der Universität Tübingen; 1991. 314 p.

[35] Tafelmaier Y. Technological variability at the beginning of the Aurignacian in Northern Spain: Implications for the Proto- and Early Aurignacian distinction [Hochschulschrift]. Mettmann: Neanderthal Museum; 2017.

[36] Sommer C. A map collection of the Paleolithic of the Swabian Jura In: Sommer C, editor. 1.0.0 ed. 2019.

[37] Conard NJ. Palaeolithic ivory sculptures from southwestern Germany and the origins of figurative art. Nature. 2003;426(6968):830–2. doi: 10.1038/nature02186 14685236

[38] Dutkiewicz E. Zeichen - Markierungen, Muster und Symbole im Schwäbischen Aurignacien. Tübingen: Kerns Verlag Tübingen; 2021.

[39] Wolf S. Schmuckstücke die Elfenbeinbearbeitung im Schwäbischen Aurignacien. Tübingen: Kerns; 2015. 316 p.

[40] Moreau L. Geißenklösterle das Gravettien der Schwäbischen Alb im europäischen Kontext. Tübingen: Kerns; 2009. 367 p.

[41] Kitagawa K, Conard NJ. Split-based points from the Swabian Jura highlight Aurignacian regional signatures. PLoS One. 2020;15(11):e0239865. doi: 10.1371/journal.pone.0239865 33170859 PMC7654757

[42] Dutkiewicz E. Markierungen, Muster und Symbole im Schwäbischen Aurignacien - Markings, Patterns, and Symbols of the Swabian Aurignacian. unpublished Dissertation. Eberhard Karls Universität Tübingen; 2018.

[43] Dutkiewicz E. The Vogelherd cave and the discovery of the earliest art - history, critics and new questions. World Heritage Paper. 2015;41(2):74–91.

[44] Wolf S, Kind CJ, Conard N. Schmuck aus dem Aurignacien von der Schwäbischen Alb im Vergleich mit Inventaren aus dem Lahntal und dem Rheinland. Archäologisches Korrespondenzblatt. 2013;43:295–313.

[45] Conard N, Malina M, Zeidi M. Neue Kunst und erste Einblicke in ungestörte Schichten am Vogelherd. Archäologische Ausgrabungen Baden-Württemberg. 2010;2009:57–61.

[46] Conard N, Zeidi M. Zur Fortsetzung der Ausgrabunen am Vogelherd im Lonetal. Archäologische Ausgrabungen Baden-Württemberg. 2011;2012:61–5.

[47] Conard N, Zeidi M, Bega J. Die letzte Kampagne der Nachgrabungen am Vogelherd. Archäologische Ausgrabungen Baden-Württemberg. 2012;2013:84–8.

[48] Boger U, Starkovich B, Conard N. New insights gained from the faunal material recovered during the latest excavations at Vogelherd Cave. Mitteilungen der Gesellschaft für Urgeschichte. 2014;23:57–81.

[49] Conard NJ. Tonnenweise Funde aus dem Abraum. Neue Grabungen im Vogelherd. Archäologie in Deutschland. 2016;6:26–7.

[50] Conard NJ, Zeidi M, Janas A. Neue Ausgrabungen am Vogelherd im Lonetal. Archäologische Ausgrabungen Baden-Württemberg. 2023;2022:57–9.

[51] Conard N, Zeidi M. Der Fortgang der Ausgrabungen am Vogelherd. Archäologische Ausgrabungen Baden-Württemberg. 2010;2011:61–4.

[52] -Schürch B, Wolf S, Schmidt P, Conard N. Mollusken der Gattung Glycymeris aus der Vogelherd-Höhle bei Niederstotzingen (Lonetal, Südwestdeutschland). Mitteilungen der Gesellschaft für Urgeschichte. 2021;29(2020):53–79.

[53] Bolus M, Conard NJ. Zur Zeitstellung von Geschossspitzen aus organischen Materialien im späten Mittelpaläolithikum und Aurignacien. Archäologisches Korrespondenzblatt. 2006;36:1–14.

[54] Conard NJ, Seidel E. Das Mammut vom Vogelherd Tübinger Funde der ältesten erhaltenen Kunstwerke; erschienen anlässlich der Ausstellung des Museums der Universität Tübingen MUT und des Instituts für Ur- und Frühgeschichte und Archäologie des Mittelalter; Museum Schloss Hohentübingen, 12. bis 21. Dezember 2008. 1. Aufl. ed. Tübingen: Museum der Universität Tübingen; 2008. 50 p.

[55] Conard NJZ, Mohsen BM. Die letzte Kampagne der Nachgrabungen am Vogelherd. Archäologische Ausgrabungen in Baden-Württemberg. 2012;2013:84–8.

[56] Conard N, Janas A, Zeidi M. Neues aus dem Lonetal: Ergebnisse von Ausgrabungen an der Fetzershaldenhöhle und dem Vogelherd. Archäologische Ausgrabungen Baden-Württemberg. 2014;2015:54–9.

[57] Conard NJ, Zeidi M, Janas A. Abschließender Bericht über die Nachgrabung am Vogelherd und die Sondage in der Wolftalhöhle. Archäologische Ausgrabungen Baden-Württemberg. 2015;2016:66–72.

[58] Hahn J. Kraft und Aggression: die Botschaft der Eiszeitkunst im Aurignacien Süddeutschlands? Tübingen: Archaeologica Venatoria; 1986. 254 p.

[59] Delporte H. Notes de géographie préhistorique: I. Les pointes d’Aurignac. Pallas Revue d’études antiques. 1958:11–29.

[60] Müller-Beck H. Eine “Wurzelindustrie” des Vogelherd-Aurignaciens. Fundberichte aus Schwaben. 1965;17:43–51.

[61] Kind C-J. Die Kratzer aus sechs Jungpaläolithischen Inventaren (Vogelherd V, Vogelherd IV, Brillenhöhle VII, Petersfels AH 2,3, Munzingen, Speckberg): Versuch einer statistischen Analyse [Hochschulschrift]. Tübingen; 1977.

[62] Valoch K. Evolution of the Palaeolithic in Central and Eastern Europe. Current Anthropol. 1968;9(5, Part 1):351–90. doi: 10.1086/200922

[63] Wagner E. Eine Löwenkopfplastik aus Elfenbein von der Vogelherdhöhle. Fundberichte aus Baden-Württemberg. 1981;6:29–58.

[64] Owen LR. Klingen- und Mikroklingentechnologie im Jungpaläolithikum Südwestdeutschlands. Archäologisches Korrespondenzblatt. 1989;19:103–15.

[65] Uthmeier T. Vier Fundplätze vom Keilberg/Stadt Regensburg und der Beginn des Jungpaläolithikums in Bayern. (unpublished Master Thesis). University Cologne; 1994.

[66] Herkert K, Siegeris M, Chang JY, Conard NJ, Floss H. Zur Ressourcennutzung später Neandertaler und früher moderner Menschen. Fallbeispiele aus dem südlichen Burgund und der Schwäbischen Alb. Mitteilungen der Gesellschaft für Urgeschichte. 2015;24:141–72.

[67] Teyssandier N, Bolus M, Conard N. The early Aurignacian in central Europe and its place in a European perspective. In: Bar-Yosef O, Zilhão J, editors. Towards a definition of the Aurignacian. Lisboa: American School of Prehistoric Research/Instituto Português de Arqueologia; 2006. p. 241–56.

[68] Burkert W. Stratigraphie und Rohmaterialnutzung um Vogelherd. unpublished Magister Thesis. Univertity Tübingen; 1991.

[69] Bolus M. Flake production in the Aurignacian of southwestern Germany: Some examples from the Swabian Jura. In: Pastoors A, Peresani M, editors. Flakes Not Blades: The Role of Flake Production at the Onset of the Upper Palaeolithic in Europe. 5. Mettmann: Wissenschaftliche Schriften des Neanderthal Museums; 2012. p. 153–64.

[70] Burkert W, Floss H. Lithic exploitation areas in the Upper Palaeolithic of West and Southwest Germany - a comparative Study. In: International Flint Symposium. Bochum: Der Anschnitt; 2005. p. 35–49.

[71] Riek G. Eine Mischkultur am Randecker Maar und deren Datierungsfrage. Berlin: de Gruyter; 1932. 257–64 p.

[72] Chang J-Y. Vogelherd. The lithic technology of the Swabian Aurignacian and its importance for early modern humans in Europe. In: Sanz N, editor. Human origin sites and the World Heritage Convention in Eurasia HEADS 4. World heritage papers. 2015. p. 50–60.

[73] Ronen A. The burins of Vogelherd Aurignacian (Germany) and those of the French Aurignacian: a comparison. Quartär. 1970;21:47–55.

[74] Conard NJ, Bolus M, Münzel SC, editors. Geißenklösterle. Chronostratigraphie, Paläoumwelt und Subsistenz im Mittel- und Jungpaläolithikum der Schwäbischen Alb. Tübingen: Kerns Verlag; 2019.

[75] Conard NJ. Excavations at Geißenklösterle Cave. In: Conard NJ, Bolus M, Münzel S, editors. Geißenklösterle - Chronostratigraphie, Paläoumwelt und Subsistenz im Mittel- und Jungpaläolithikum der Schwäbischen Alb. Tübingen: Kerns; 2019. p. 9–21.

[76] Higham T, Basell L, Jacobi R, Wood R, Ramsey CB, Conard NJ. Τesting models for the beginnings of the Aurignacian and the advent of figurative art and music: the radiocarbon chronology of Geißenklösterle. J Hum Evol. 2012;62(6):664–76. doi: 10.1016/j.jhevol.2012.03.003 22575323

[77] Conard NJ, Rots V. Rope making in the Aurignacian of Central Europe more than 35,000 years ago. Sci Adv. 2024;10(5):eadh5217. doi: 10.1126/sciadv.adh5217 38295167 PMC10830101

[78] Boëda E, Geneste J-M, Meignen L. Identification de chaînes opératoires lithiques du Paléolithique ancien et moyen. Paléo, Revue d’Archéologie Préhistorique. 1990;43–80.

[79] Geneste J-M. Systèmes techniques de production lithique: variations techno-economiques dans les processus de réalisation des outillages paléolithiques. Techniques et Culture. 1991;17–18:1–35.

[80] Hahn J. Erkennen und Bestimmen von Steinartefakten Artefaktmorphologie I: Grundbegriffe, Pseudoartefakte u. Grundproduktion; Vorlesungsskript. Tübingen: Verl. Archaeologica Venatoria; 1989. 78 p.

[81] Tixier J. Typologie de l’Epipaléolithique du Maghreb. Paris; 1963.

[82] Schürch B, Rots V, Conard NJ. Technofunctional Analysis Reveals the Role of Carinated Artifacts and End-Scrapers in the Aurignacian of Vogelherd Cave. J Archaeol Method Theory. 2025;32(4):61. doi: 10.1007/s10816-025-09728-2

[83] Klaric L, Lev S, Giria Y, Polanská M. Couteaux de Kostienki et lames aménagées par technique de Kostienki. Bulletin de la Société préhistorique de France. 2015;112:421–74.

[84] Teyssandier N, Bon F, Bordes JG. Within projectile range. Some thoughts on the appearance of the Aurignacian in Europe. J Anthropol Res. 2010;66:209–29. doi: 10.2307/27820882

[85] Owen LR. Blade and microblade technology: selected assemblages from the North American Arctic and the Upper Paleolithic of Southwest Germany [Hochschulschrift]. Oxford: British Archaeological Reports; 1988.

[86] Brun-Ricalens F. Réflexions préliminaires sur le comportement lithotechnologique et l’occupation du territoire du pays des Serres à l’Aurignacien: Le gisement de “toulousète” à Beauville (Lot-et-Garonne). Paléo. 1993;5. doi: 10.3406/pal.1993.1108

[87] Chiotti L. Les industries lithiques des niveaux aurignaciens de l’abri Pataud, Les Eyzies-de-Tayac (Dordogne): étude technologique et typologique - Tome II. 1999.

[88] Aleo A, Duches R, Falcucci A, Rots V, Peresani M. Scraping hide in the early Upper Paleolithic: Insights into the life and function of the Protoaurignacian endscrapers at Fumane Cave. Archaeol Anthropol Sci. 2021;13(8):137. doi: 10.1007/s12520-021-01367-4

[89] Dinnis R. On the technology of late Aurignacian burin and scraper production, and the importance of the Paviland lithic assemblage and the Paviland burin. Lithics J Lithic Stud Soc. 2008;29:18–35.

[90] Hays MA, Lucas G. A Technological and Functional Analysis of Carinates from Le Flageolet I, Dordogne, France. J Field Archaeol. 2000;27(4):455–65. doi: 10.1179/jfa.2000.27.4.455

[91] Domingo R, Mazo C, Utrilla P. Hunting camps and nucleiform endscrapers in the Cantabrian Lower Magdalenian: A lithic microwear analysis. Quater Int. 2012;272–273:105–10. doi: 10.1016/j.quaint.2012.03.027

[92] Dibble HL, McPherron SP. Truncated-faceted pieces: hafting modification, retouch, or cores? In: Tools versus cores: new approaches in the analysis of stone tool assemblages. Cambridge Scholars Publications; 2007. p. 75–90.

[93] Frick JA. Kostenki-Enden (Dorsalabbau an Grundformen). In: Floss H, editor. Steinartefakte vom Altpaläolithikum bis in die Neuzeit. 2nd ed. Tübingen: Tübingen Publications in Prehistory, Kerns; 2013. p. 459–66.

[94] Shalagina AV, Krivoshapkin AI, Kolobova KA. Truncated-faceted pieces in the paleolithic of northern asia. Archaeol Ethnol Anthropol Eurasia. 2015;43(4):33–45. doi: 10.1016/j.aeae.2016.02.004

[95] Dinnis R. The timing of Aurignacian occupation of the British Peninsula. Quartär. 2012;59:67–83. doi: 10.7485/QU59_03

[96] Ducasse S, Langlais M. Interprétation technologique et discussion autour du statut culturel des « pièces de la Bertonne ». L’exemple de la série lithique de Seyresse (Landes, France). Paleo. 2008;20:59–88. doi: 10.4000/paleo.1674

[97] Chemana L, Holzem N, Pelegrin J, Bazin P. La fonction des pièces de la Bertonne: un problème en partie résolu. PALEO. 2009-2010;21:65–102. doi: 10.4000/paleo.1750

[98] Lenoir M. La pièce de la Bertonne “fossile directeur” du Magdalénien ancien? Bulletin de la Société préhistorique de France. 1987;86(6):167–71. doi: 10.3406/bspf.1987.9826

[99] Lombao D, Falcucci A, Moos E, Peresani M. Unravelling technological behaviors through core reduction intensity. The case of the early Protoaurignacian assemblage from Fumane Cave. J Archaeol Sci. 2023;160:105889. doi: 10.1016/j.jas.2023.105889

[100] Boëda E. De la surface au volume, analyse des conceptions, des débitages Levallois et laminaire. Paléolitique moyen et Paléolothique suüérieur ancian en Europe. Mémoires du Musée de Préhistoire d’Île de France. 1990;3:75–92.

[101] Kuhn SL, Zwyns N. Rethinking the initial Upper Paleolithic. Quat Int. 2014;347:29–38. doi: 10.1016/j.quaint.2014.05.040

[102] Kind C-J. Ulm-Eggingen. Die Ausgrabungen 1982 bis 1985 in der bandkeramischen Siedlung und der mittelalterlichen Wüstung. Mit Beiträgen von G. Dombek, S. Gregg, U. Gross, M. Kokabi und H.-Ch. Strien. Stuttgart: Konrad Theiss Verlag; 1989.

[103] Pastoors A. Die mittelpaläolithische Freilandstation Salzgitter-Lebenstedt (Niedersachsen). Archaologisches Korrespondenzblatt. 1999;29:1–9.

[104] Pastoors A. Die mittelpaläolithische Freilandstation von Salzgitter-Lebenstedt Genese der Fundstelle und Systematik der Steinbearbeitung. Salzgitter: Archiv d. Stadt Salzgitter; 2001. p. 347.

[105] Conard NJ, Adler DS, Forrest DT, Kaszas PJ. Preliminary archaeological results from the 1991-1993 excavations in Wallertheim. Archäologisches Korrespondenzblatt. 1995;25:13–27.

[106] Schürch B, Wettengl S, Fröhle S, Conard N, Schmidt P. The origin of chert in the Aurignacian of Vogelherd Cave investigated by infrared spectroscopy. PLoS One. 2022;17(8):e0272988. doi: 10.1371/journal.pone.0272988 35976960 PMC9385009

[107] Burkert W. Lithische Rohmaterialversorgung im Jungpaläolithikum des südöstlichen Baden-Württemberg [Dissertation]. 2001.

[108] Çep B. Ausgangsbasis oder Versorgungsstandort? Raumnutzung im Mittel- und Jungpaläolithikum des Ach- und Blautals bei Blaubeuren. Quartär. 2013;60:61–83. doi: 10.7485/QU60_3

[109] Cziesla E. Refitting of stone artefacts. In: Cziesla E, Eickhoff S, Arts N, Winter D, editors. The Big Puzzle International Symposium on Refitting Stone Artefacts. Bonn: Holos; 1990. p. 9–44.

[110] Alperson-Afil N, Goren-Inbar N. Scarce but Significant: The Limestone Component of the Acheulean Site of Gesher Benot Ya’aqov, Israel. In: Haidle MN, Conard NJ, Bolus M, editors. The Nature of Culture: Based on an Interdisciplinary Symposium ‘The Nature of Culture’, Tübingen, Germany. Vertebrate Paleobiology and Paleoanthropology. Dordrecht: Springer Science+Business Media; 2016.

[111] Pelegrin J. Les techniques de débitage laminaire au Tardiglaciaire: critères de diagnose et quelques réflexions. In: Valentin B, Bodu P, Christensen M, editors. L’Europe centrale et septentrionale au Tardiglaciaire Confrontation des modéles régionaux de peuplement. 7. Nemours: APRAIF, Mémoire du Musée de Préhistoire d’Ile-de-France; 2000. p. 73–86.

[112] Pelegrin J. Sur une recherche technique expérimentale des techniques de débitage laminaire. Archéologie expérimentale. 1991;2:118–28.

[113] Driscoll K, García-Rojas M. Their lips are sealed: identifying hard stone, soft stone, and antler hammer direct percussion in Palaeolithic prismatic blade production. J Archaeol Sci. 2014;47:134–41. doi: 10.1016/j.jas.2014.04.008

[114] Niven L. The palaeolithic occupation of Vogelherd Cave implications for the subsistence behavior of late Neanderthals and early modern humans. Tübingen: Kerns; 2006. p. 312.

[115] Toniato G, Münzel SC, Starkovich B, Conard NJ. Middle and Upper Palaeolithic Bone Retouchers from the Swabian Jura: Raw Materials, Curation and Use. In: Hutson JM, García-Moreno A, Noack ES, Turner E, Villaluenga A, Gaudzinski-Windheuser S, editors. The Origins of Bone Tool Technologies. RGZM – Tagung. Mainz: Verlag des Römisch-Germanischen Zentralmuseums; 2018. p. 251–67.

[116] Taute W. Retoucheure aus Knochen, Zahnbein und Stein vom Mittelpaläolithikum bis zum Neolithikum. Fundberichte aus Schwaben. 1965;17:76–102.

[117] Uthmeier T. Micoquien, Aurignacien und Gravettien in Bayern eine regionale Studie zum übergang vom Mittel- zum Jungpaläolithikum. Bonn: Habelt; 2004. 500, 70 p.

[118] Zwyns N. The Initial Upper Paleolithic in Central and East Asia: Blade Technology, Cultural Transmission, and Implications for Human Dispersals. J Paleo Arch. 2021;4(3):19. doi: 10.1007/s41982-021-00085-6

[119] Geneste J-M. Les industries de la Grotte Vaufrey: technologie du de´ bitage, e´conomie et circulation de la matière première lithique. In: Rigaud J-P, editor. La Grotte Vaufrey—paléoenvironnement, chronologie, activitès humaines. Châlons-sur-Marne: Société Préhistorique Française: Mémoires de la Société Préhistorique Française; 1988. p. 441–518.

[120] Mathias C, Bourguignon L. Cores-on-flakes and ramification during the middle palaeolithic in Southern France: A gradual process from the early to late middle palaeolithic? J Archaeol Sci Rep. 2020;31:102336. doi: 10.1016/j.jasrep.2020.102336

[121] Rios-Garaizar J, Eixea A, Villaverde V. Ramification of lithic production and the search of small tools in Iberian Peninsula Middle Paleolithic. Quater Int. 2015;361:188–99. doi: 10.1016/j.quaint.2014.07.025

[122] Sonneville-Bordes DD. Faciès germanique de l’Aurignacien typique. Bulletin de la Société préhistorique française. 1971:9–14.

[123] Chiotti L. Some Evidence for Flake Production in the Early Aurignacian: Examples from the Pataud and Castanet Rock Shelters (France). In: Pastoors A, Peresani M, editors. Flakes not Blades: The Role of Flake Production at the Onset of the Upper Palaeolithic in Europe. Wissenschaftliche Schriften des Neanderthal Museums. 5. Mettmann: Neanderthal Museum; 2012.

[124] Binford LR. Isolating the transition to cultural adaptions: An organizational approach. In: Trinkhaus E, editor. The emergence of modern humans: Biocultural adaptions in the later pleitocene. Cambridge: Cambridge University Press; 1989. p. 18–41.

[125] Gould RA, Saggers S. Lithic procurement in central Australia: A closer look at Binford’s idea of embeddedness in archaeology. American Antiquity. 1985;50(1):117–36. doi: 10.2307/280637

[126] Binford LR. Organization and formation processes: looking at curated technologies. J Anthropol Res. 1979;35(3):255–73.

[127] Binford LR. Dimensional analysis of behavior and site structure: learning from an Eskimo hunting stand. American Antiquity. 1978;43:330–61.

[128] Binford LR. Forty seven Trips: A Case Study in the Character of Archaeological Formation Processes. In: Wright RVS, editor. Stone Tools als Cultural Markers. Canberra: Australian Institute of Aboriginal Studies; 1977. p. 24–36.

[129] Andrefsky W Jr. Raw-Material Availability and the Organization of Technology. Am Antiq. 1994;59(1):21–34. doi: 10.2307/3085499

[130] Conard NJ. The Vogelherd Horse and the Origins of Art. Tübingen: Mut; 2016. 89 p.

[131] Münzel SC, Wolf S, Drucker DG, Conard NJ. The exploitation of mammoth in the Swabian Jura (SW-Germany) during the Aurignacian and Gravettian period. Quater Int. 2017;445:184–99. doi: 10.1016/j.quaint.2016.08.013

[132] Niven L. From carcass to cave: large mammal exploitation during the Aurignacian at Vogelherd, Germany. J Hum Evol. 2007;53(4):362–82. doi: 10.1016/j.jhevol.2007.05.006 17663999

[133] Bloß T. Palaeoproteomics (ZooMS) Analysis of Prehistoric Remains from Vogelherd Cave, Germany. unpublished: Universität Wien; 2023.

[134] Wang N, Nicholas J. C, Douka K. Integrating Morphological and ZooMS-Based Approaches to Zooarchaeology at Vogelherd Cave in Southwestern Germany. PalaeoAnthropology. 2024;(Special Issue: Integrating ZooMS and Zooarchaeology: Methodological Challenges and Interpretive Potentials). doi: 10.48738/2024.iss2.1094

[135] Schürch B, Venditti F, Wolf S, Conard NJ. Glycymeris molluscs in the context of the Upper Palaeolithic of Southwestern Germany. Quartär. 2023;68(2021):131–56.

[136] Moreau L. Le Gravettien ancien d’Europe centrale revisité: mise au point et perspectives. L’Anthropologie. 2012;116(5):609–38. doi: 10.1016/j.anthro.2011.10.002

[137] Münzel S. Die jungpleistozäne Großsäugerfauna aus dem Geißenklösterle. In: Conard NJ, Bolus M, Münzel SC, editors. Geißenklößterle Chronostratigraphie, Paläoumwelt und Subsistenz im Mittel- und Jungpaläolithikum der Schwäbischen Alb. Tübingen: Kerns; 2019.

[138] Floss H, Schürch B. Paläolithische Oberﬂächenfunde von der Blaubeurer Alb. Mitteilungen der Gesellschaft für Urgeschichte. 2015;24:121–40.

[139] Floss H, Fröhle S, Schürch B, Wettengl S. Open Air Occupations In A Cave Dominated Archaeological Landscape – New Perspectives On The Palaeolithic Of The Swabian Jura (Southern Germany). Anthropologie. 2017;1/2(Focus on the lithics. Special issue on the occasion of the 65th birthday of Martin Oliva):43–73.

[140] Floss H, Fröhle S, Poenicke H-W, Wettengl S. Die mittel- und jungpaläolithische Freilandfundstelle Börslingen-Eisenberg (Alb-Donau-Kreis). Archäologisches Korrespondenzblatt. 2015;45(4):459–73.

[141] Schürch B. Paläolithische Freilandfundstellen auf der Blaubeurer Alb. Ulm und Oberschwaben – Zeitschrift für Geschichte, Kunst und Kultur. 2017;60:9–27.

[142] Bataille G. Der Übergang vom Mittel- zum Jungpaläolithikum auf der Halbinsel Krim und in der Kostenki-Borshchevo-Region am Mittel-Don. Köln: University Cologne; 2013.

[143] Bataille G, Tafelmaier Y, Weniger G-C. Living on the edge – A comparative approach for studying the beginning of the Aurignacian. Quater Int. 2018;474:3–29. doi: 10.1016/j.quaint.2018.03.024

[144] Vanhaeren M, d’Errico F. Aurignacian ethno-linguistic geography of Europe revealed by personal ornaments. J Archaeol Sci. 2006;33(8):1105–28. doi: 10.1016/j.jas.2005.11.017

[145] Conard NJ. Cultural modernity: consensus or conundrum? Proc Natl Acad Sci U S A. 2010;107(17):7621–2. doi: 10.1073/pnas.1001458107 20410459 PMC2867877

