# Supplementary figures and images for: Reconstructing flexible pathways of Aurignacian blade and bladelet production at Vogelherd
Source: PLoS One. 2025 Sep 16;20(9):e0331921. doi: 10.1371/journal.pone.0331921 (PMC12440199; doi:10.1371/journal.pone.0331921)

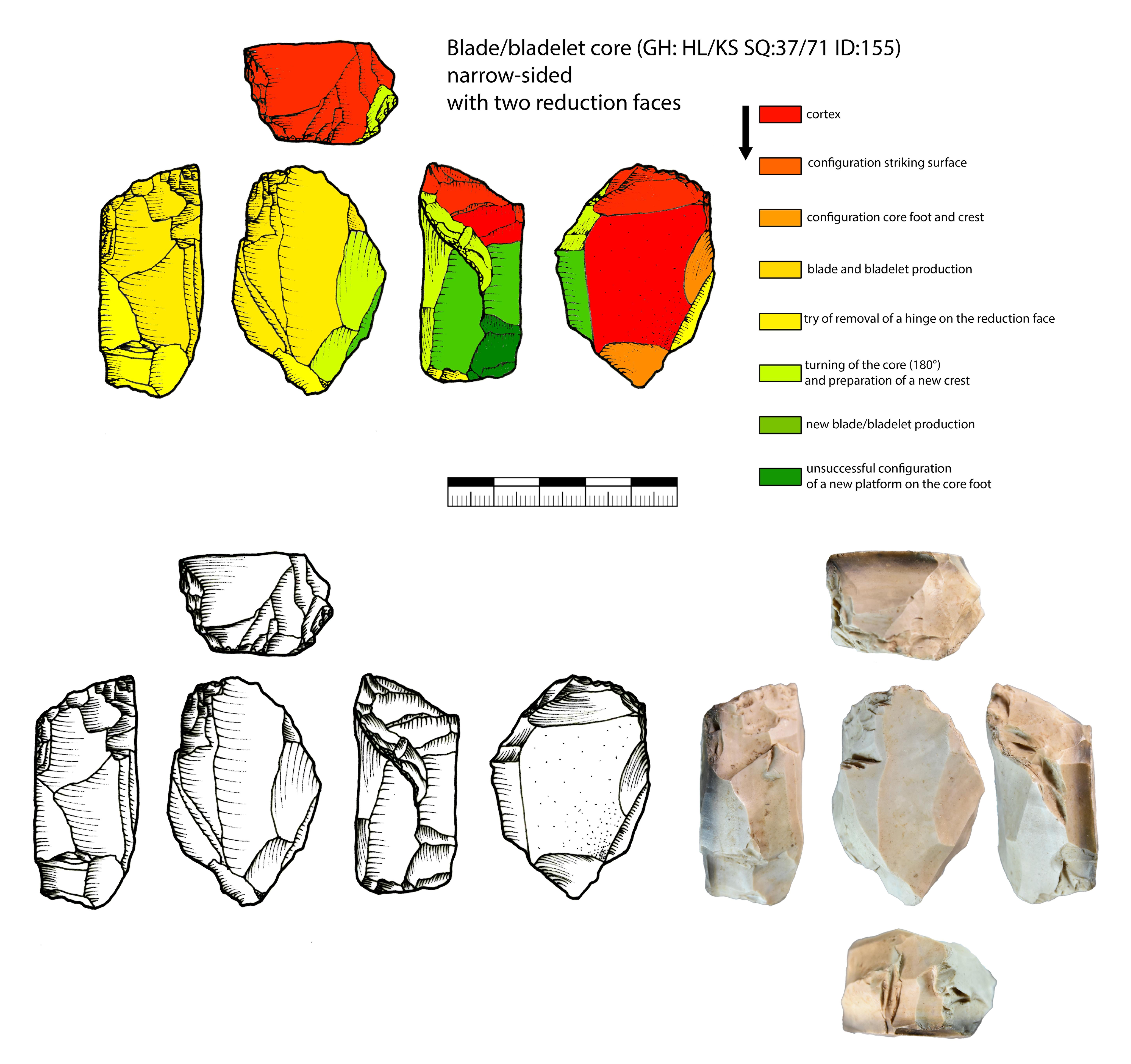

Supplement: S1 Fig — (TIF) [file pone.0331921.s005.tif]

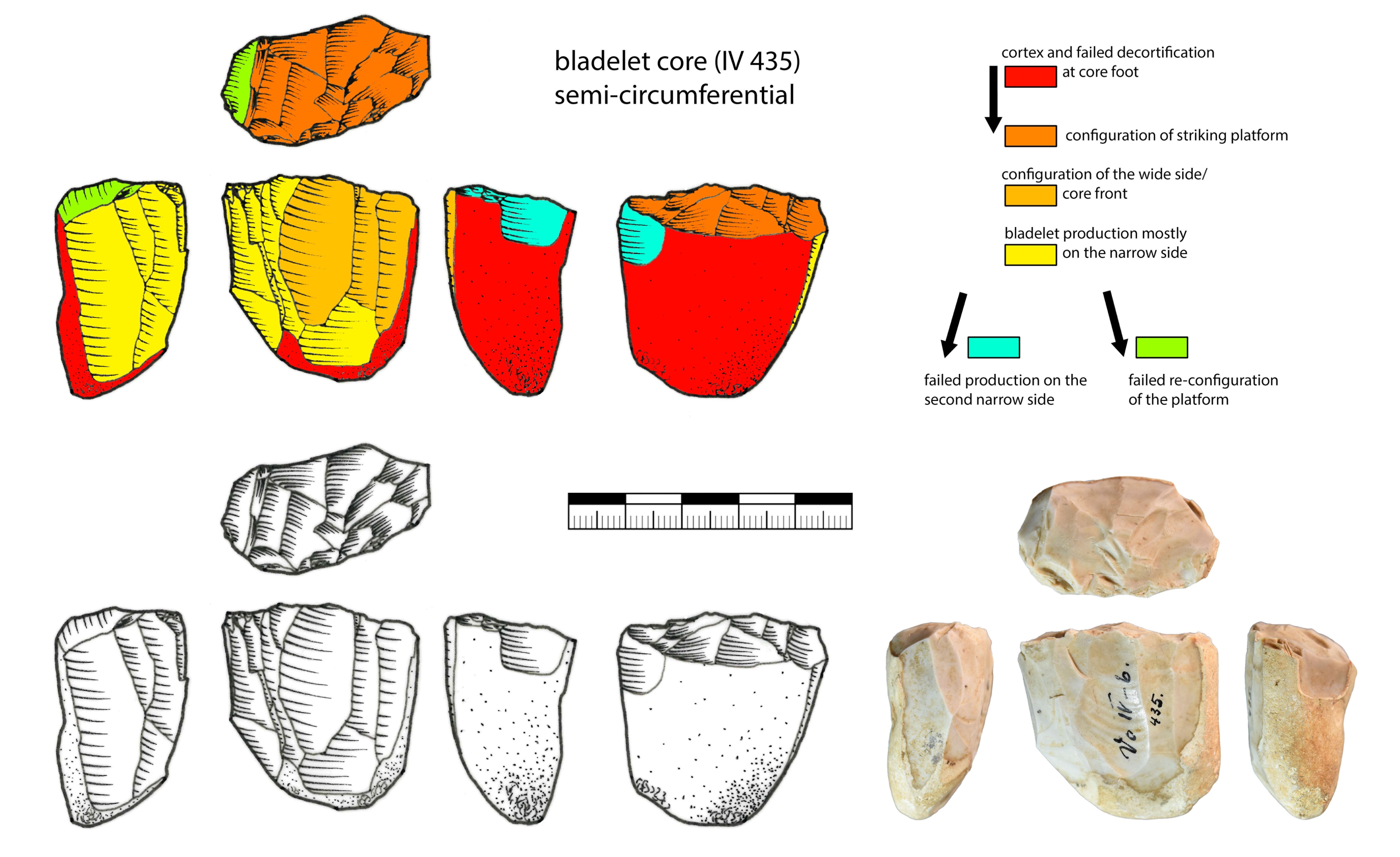

Supplement: S2 Fig — (TIF) [file pone.0331921.s006.tif]

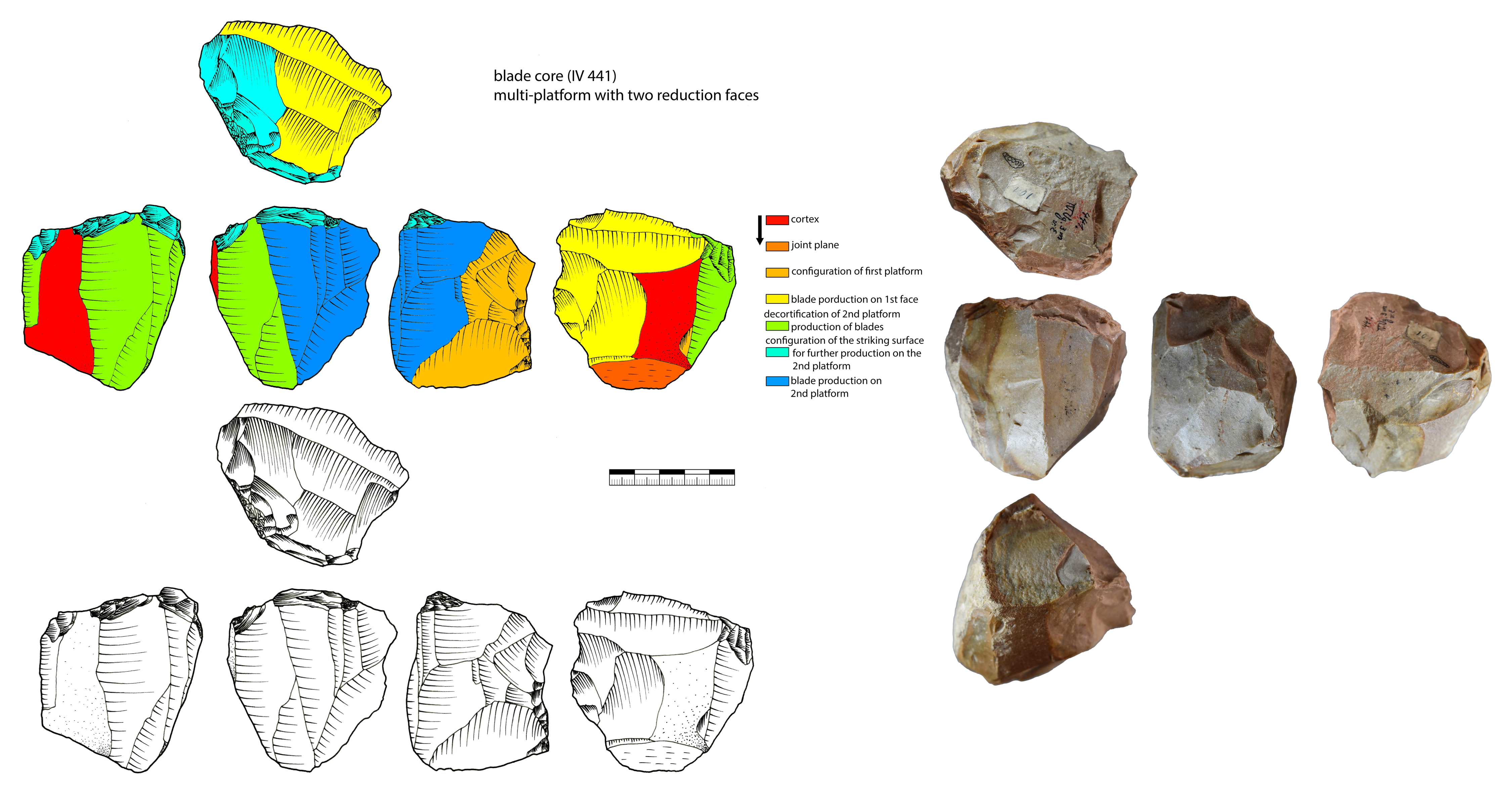

Supplement: S3 Fig — (TIF) [file pone.0331921.s007.tif]

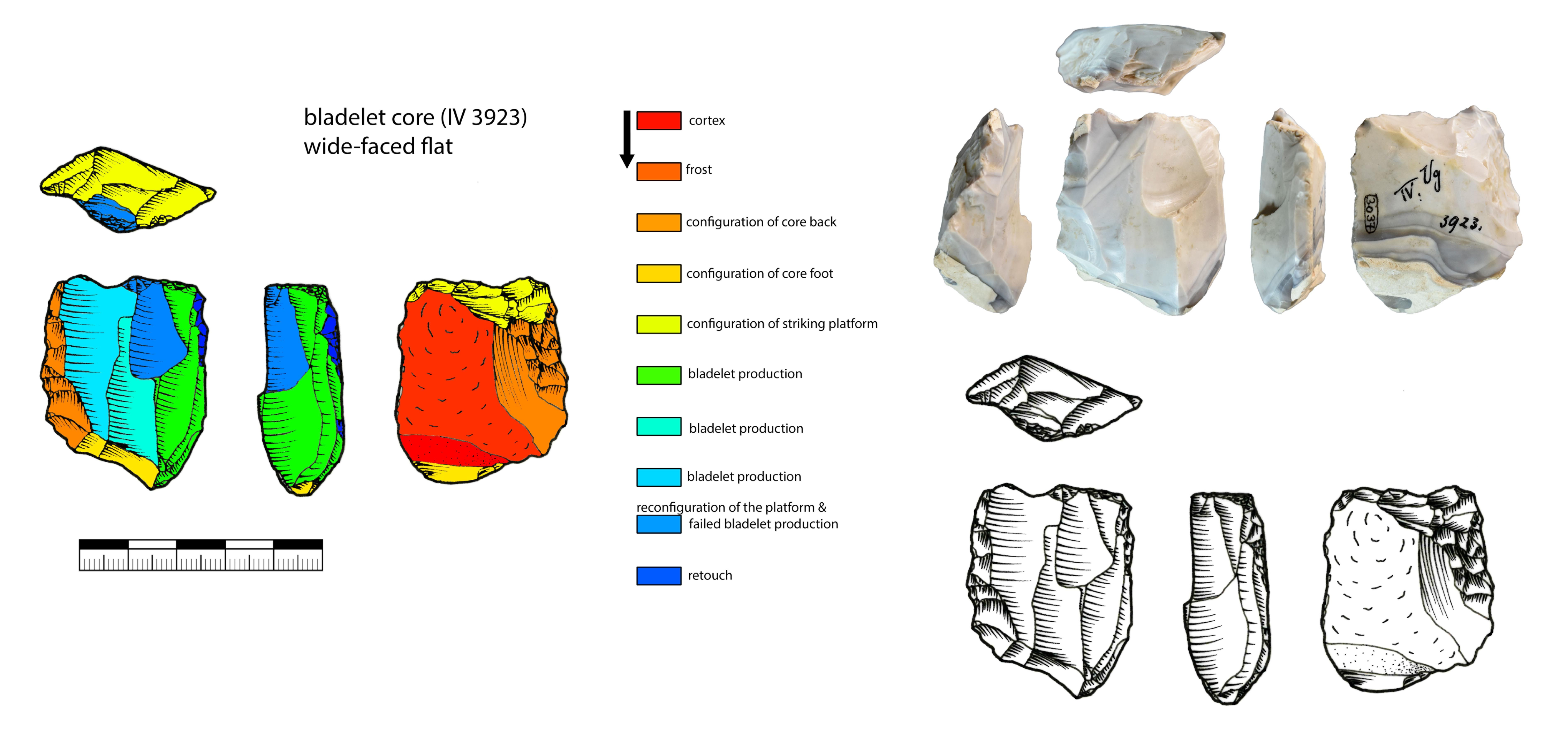

Supplement: S4 Fig — (TIF) [file pone.0331921.s008.tif]

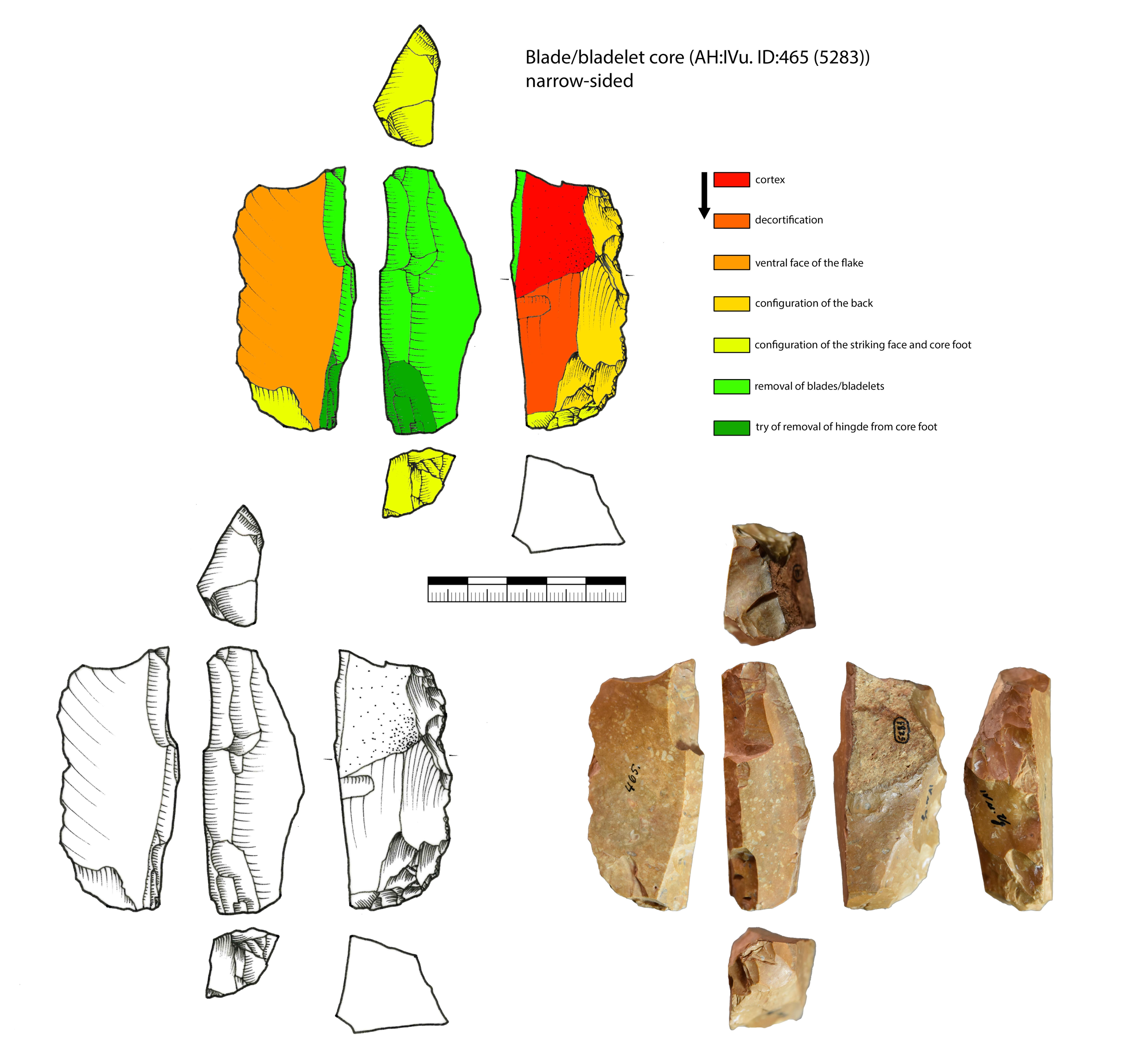

Supplement: S5 Fig — (TIF) [file pone.0331921.s009.tif]

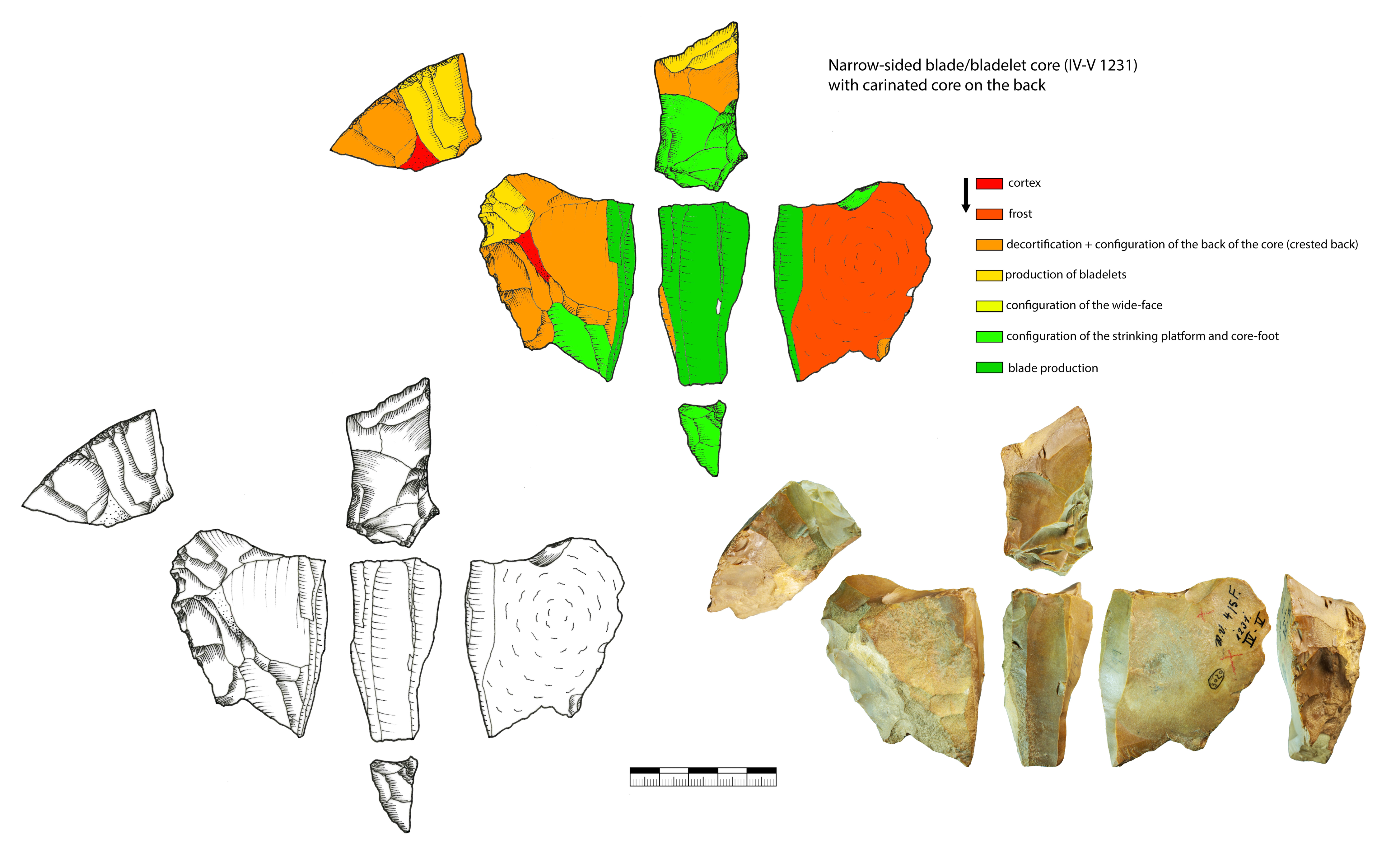

Supplement: S6 Fig — (TIF) [file pone.0331921.s010.tif]

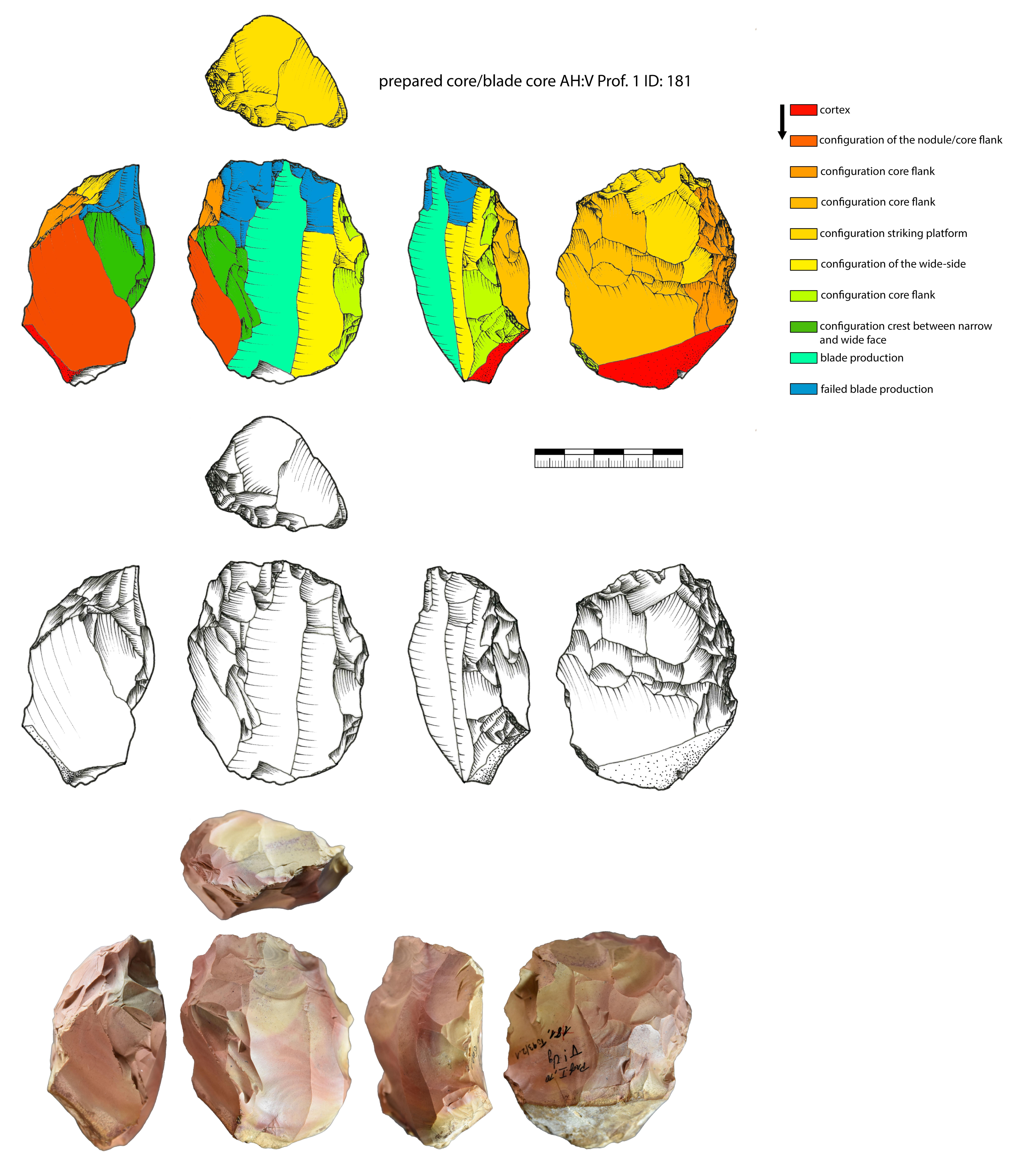

Supplement: S7 Fig — (TIF) [file pone.0331921.s011.tif]

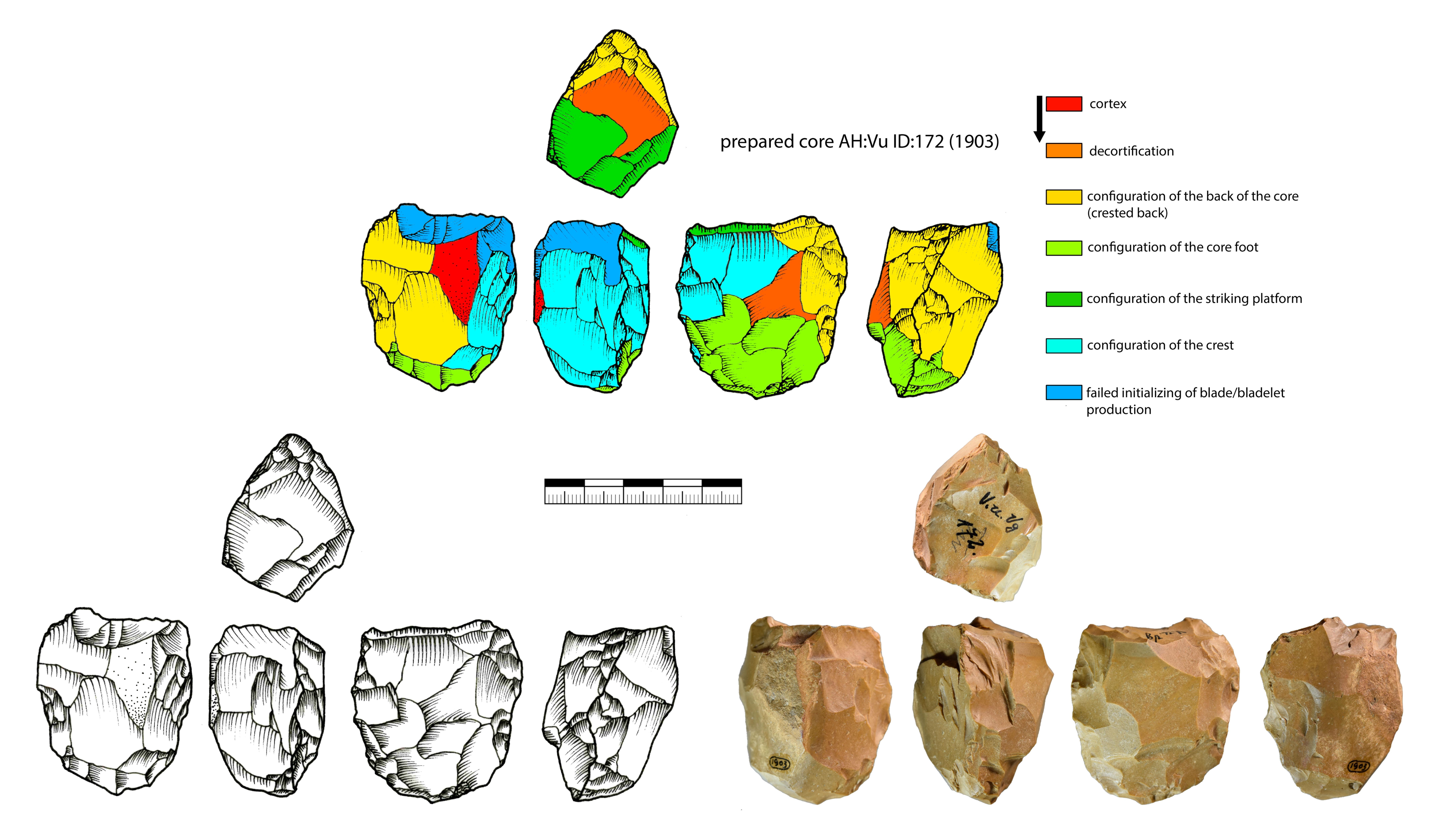

Supplement: S8 Fig — (TIF) [file pone.0331921.s012.tif]

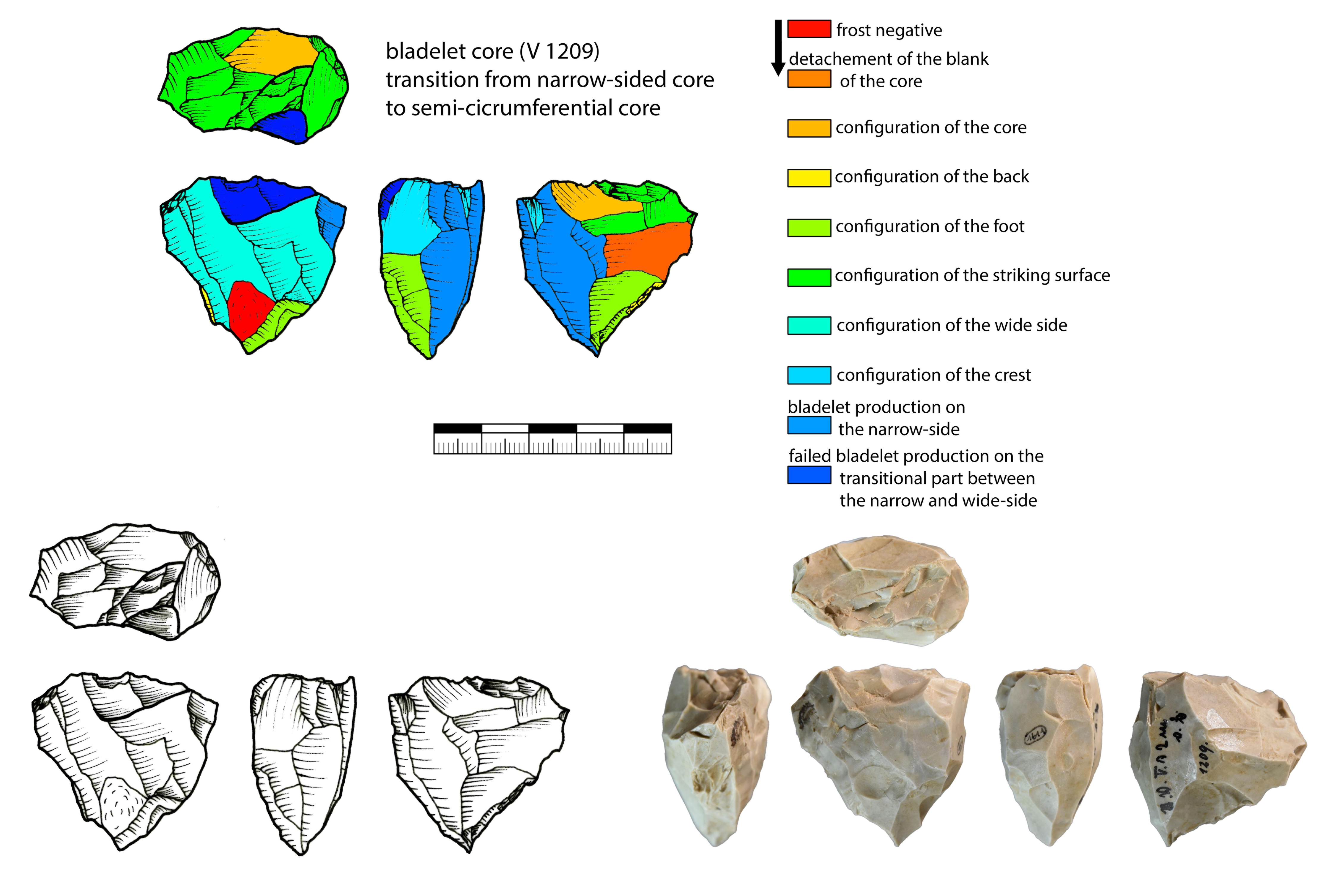

Supplement: S9 Fig — (TIF) [file pone.0331921.s013.tif]

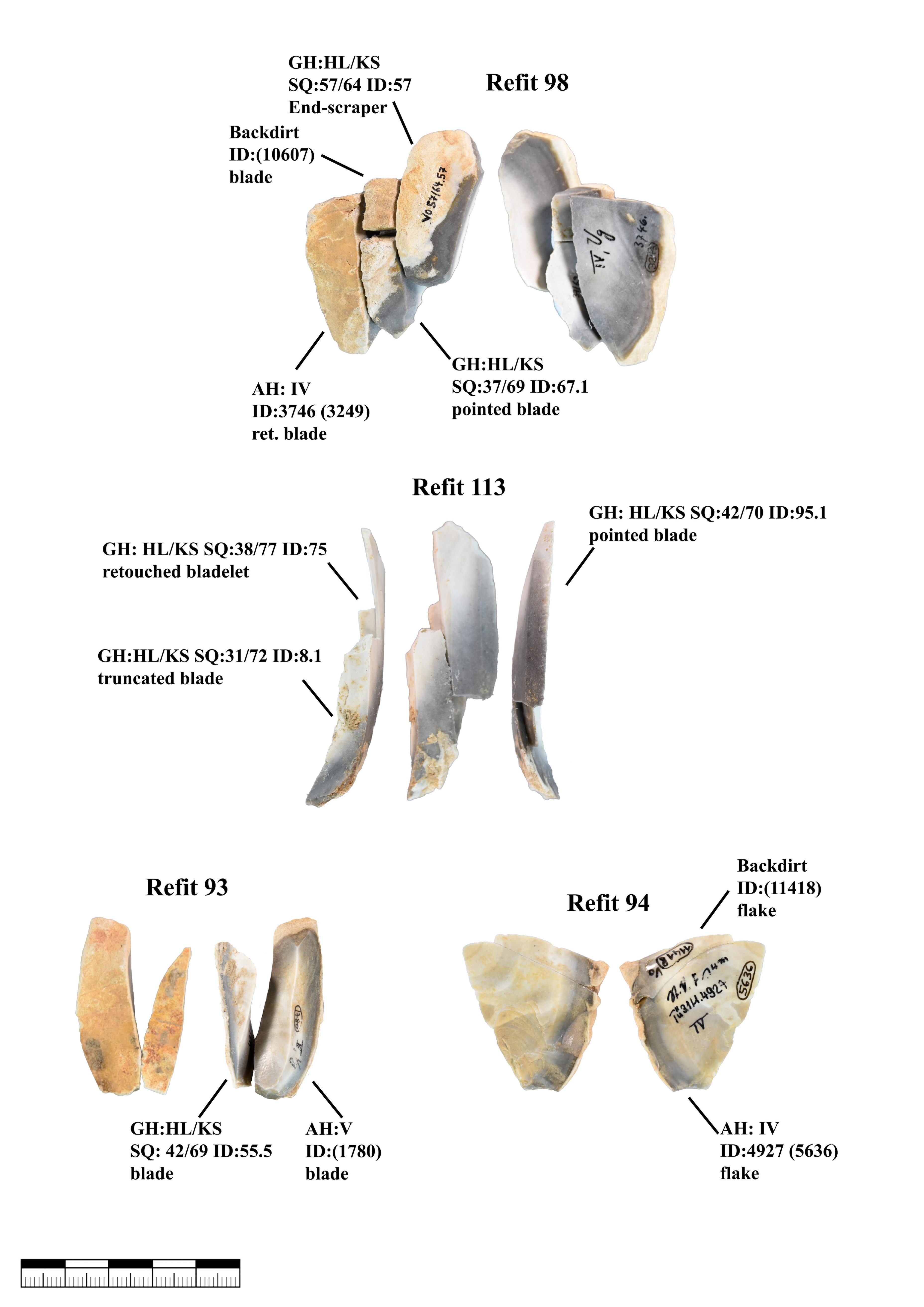

Supplement: S10 Fig — (TIF) [file pone.0331921.s014.tif]

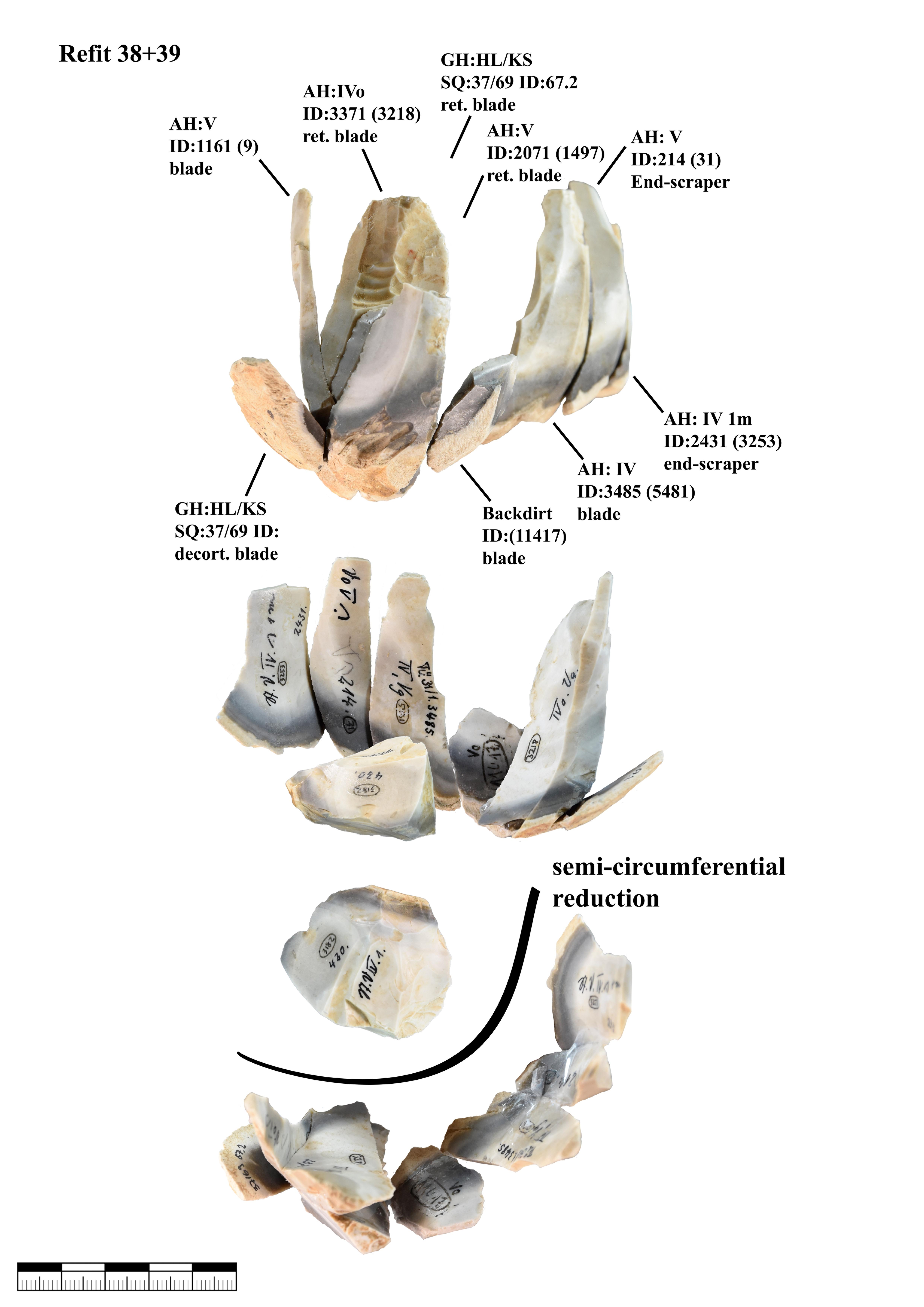

Supplement: S11 Fig — (TIF) [file pone.0331921.s015.tif]

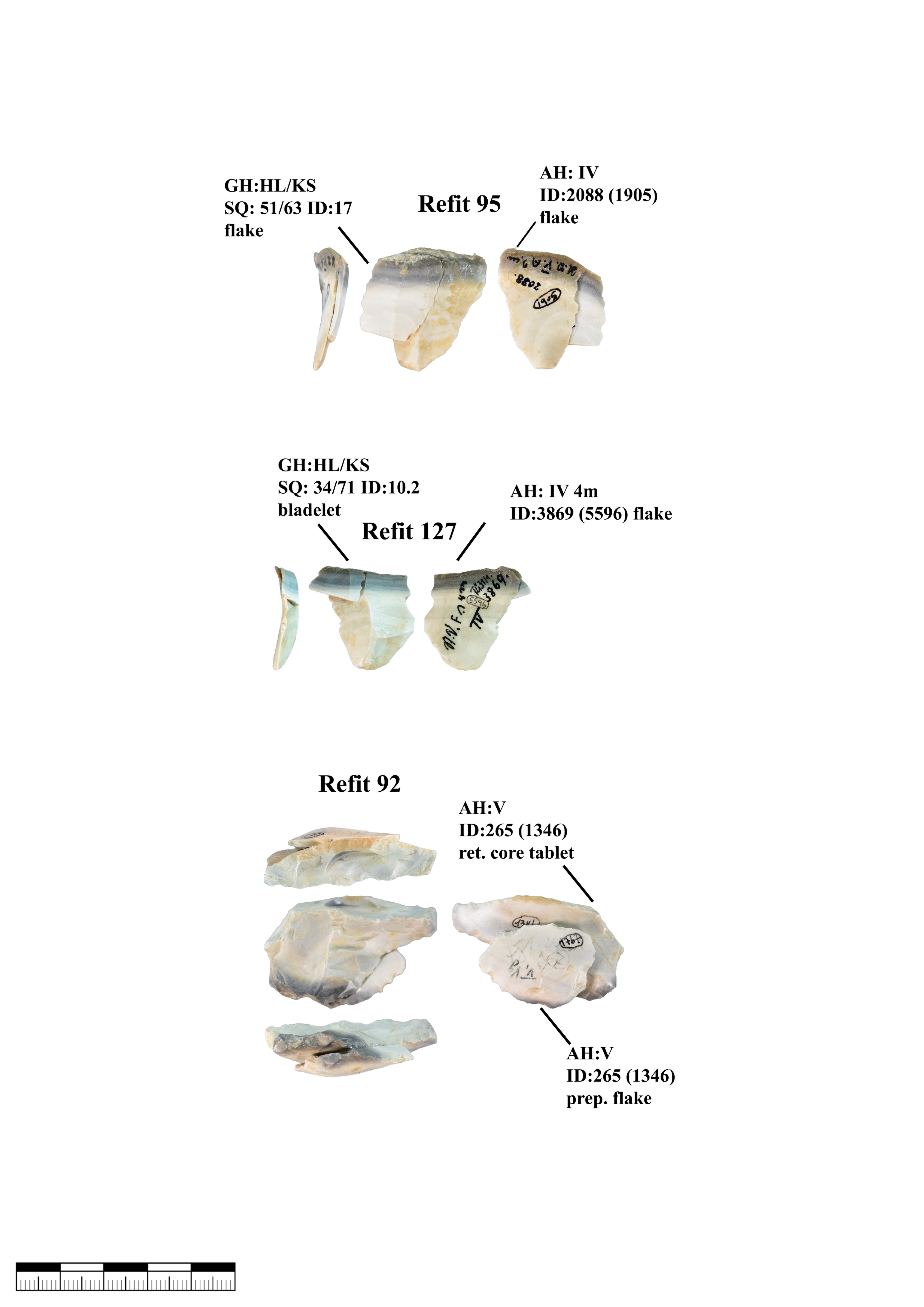

Supplement: S12 Fig — (TIF) [file pone.0331921.s016.tif]

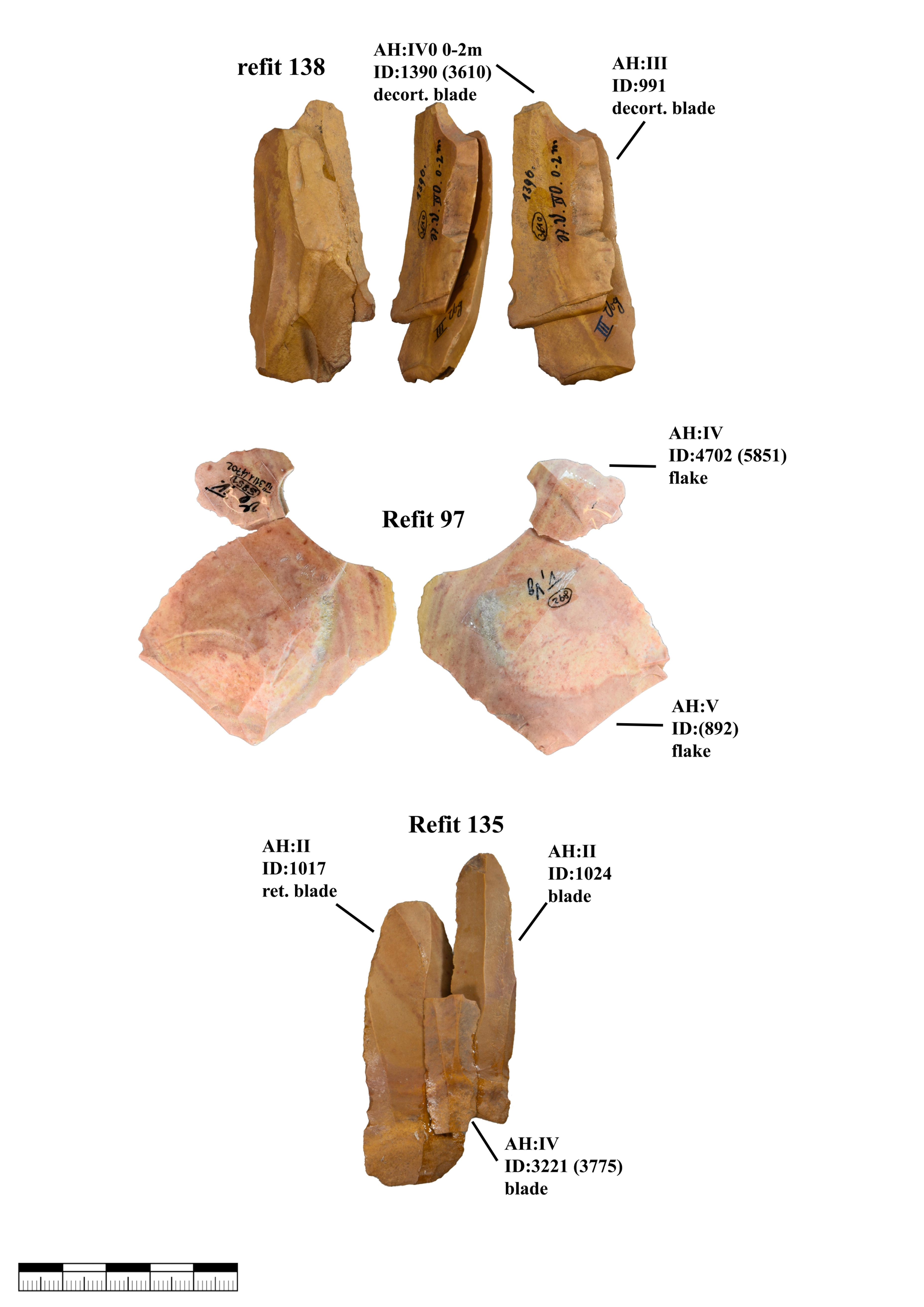

Supplement: S13 Fig — (TIF) [file pone.0331921.s017.tif]

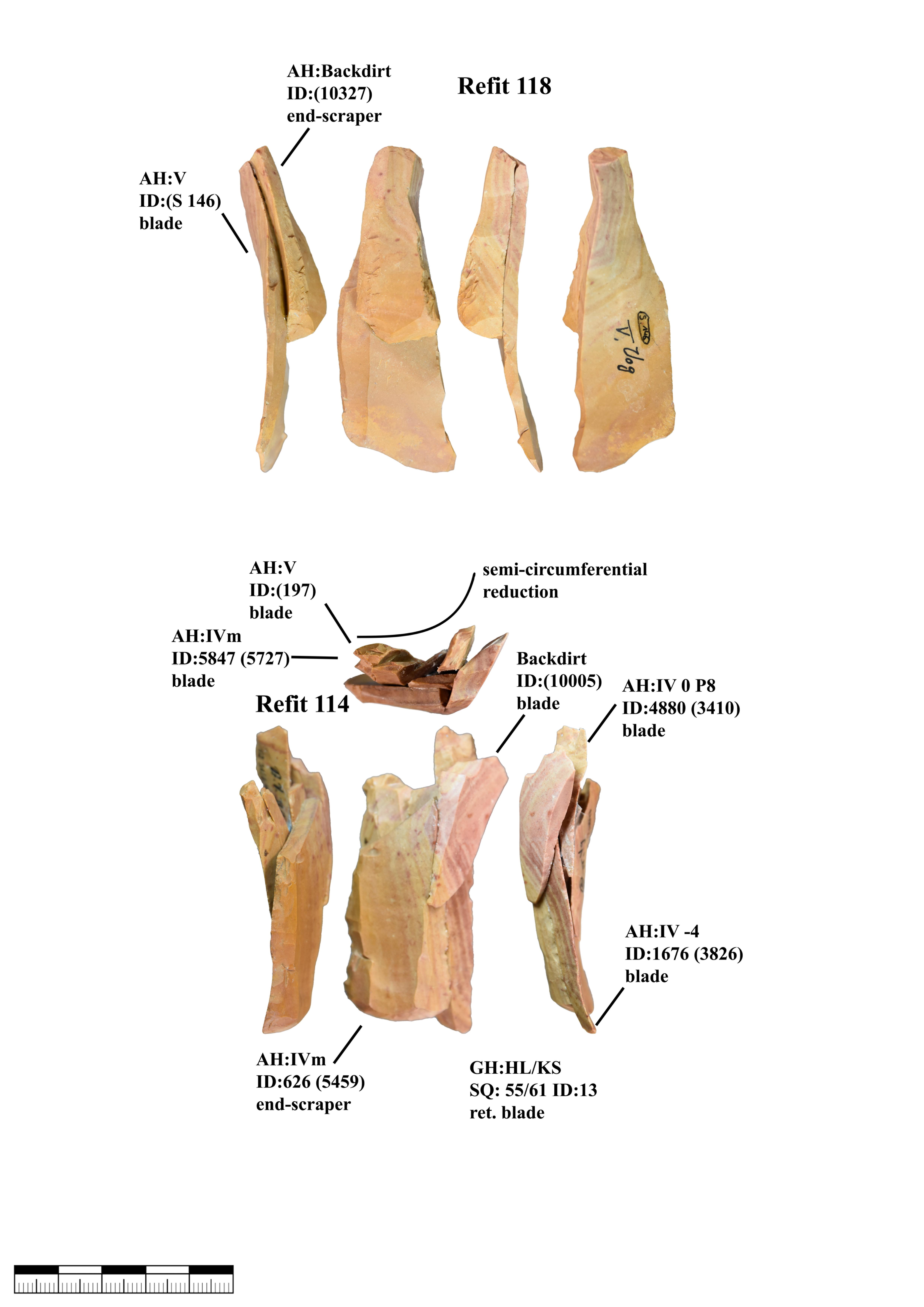

Supplement: S14 Fig — (TIF) [file pone.0331921.s018.tif]

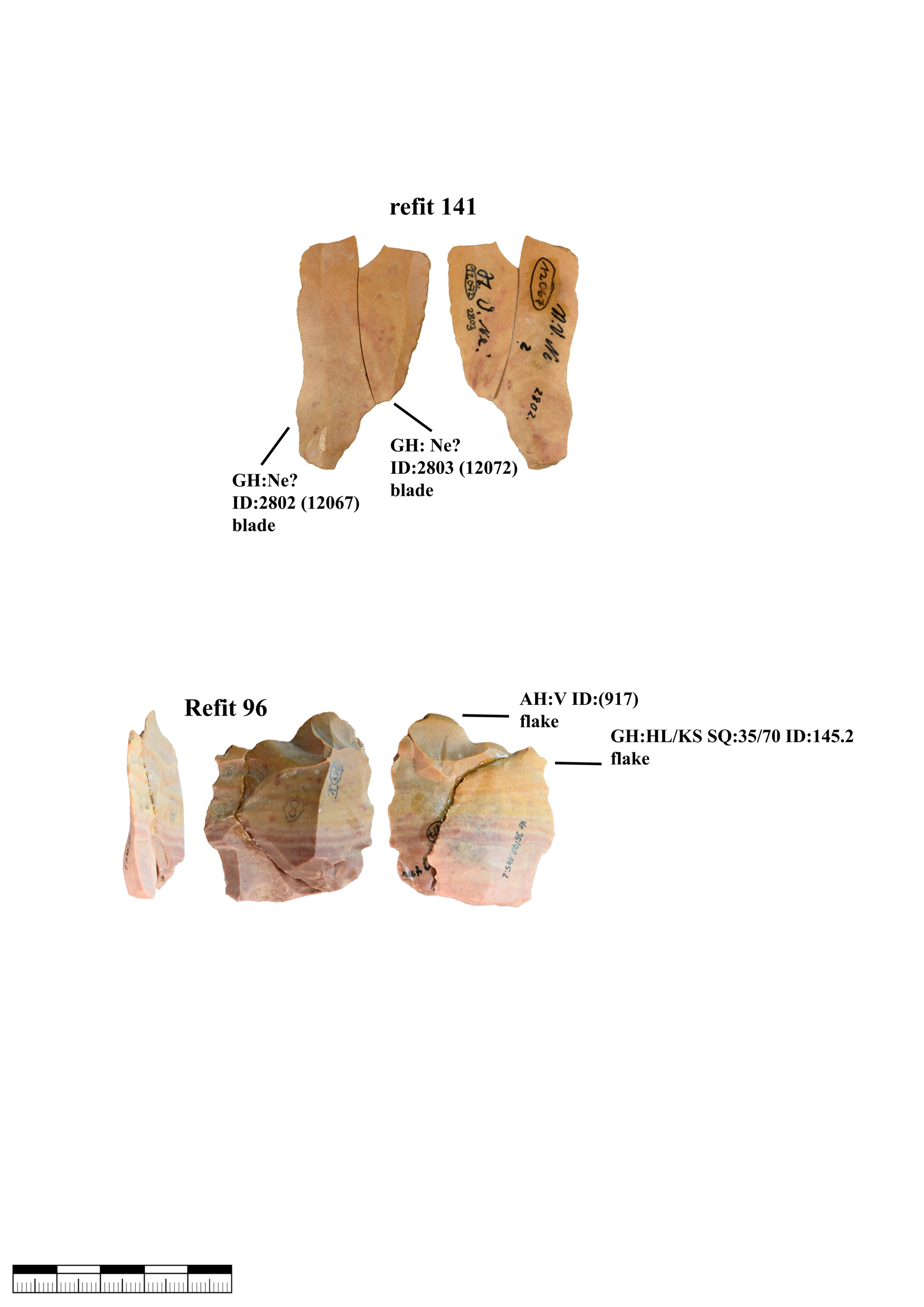

Supplement: S15 Fig — (TIF) [file pone.0331921.s019.tif]

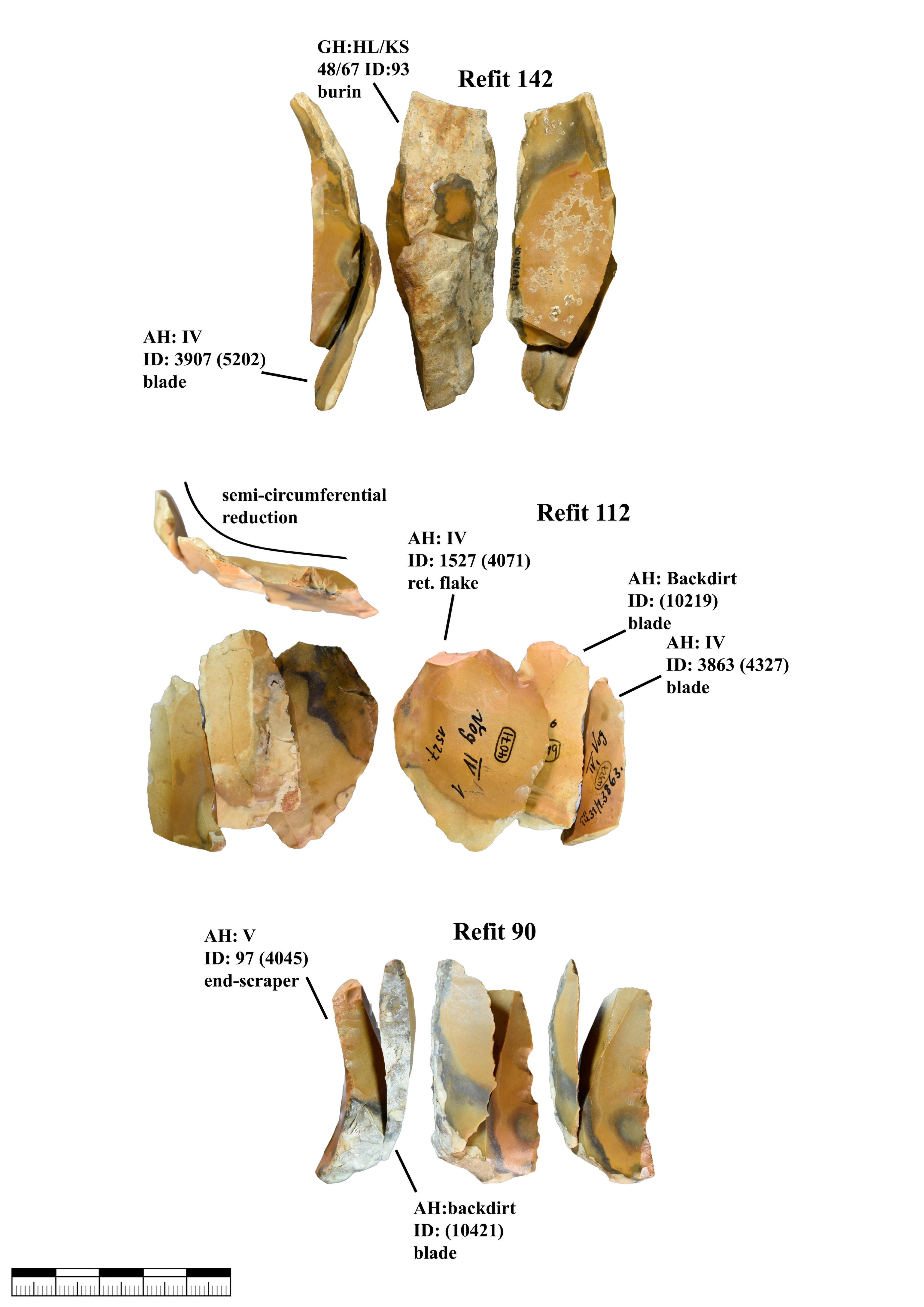

Supplement: S16 Fig — (TIF) [file pone.0331921.s020.tif]

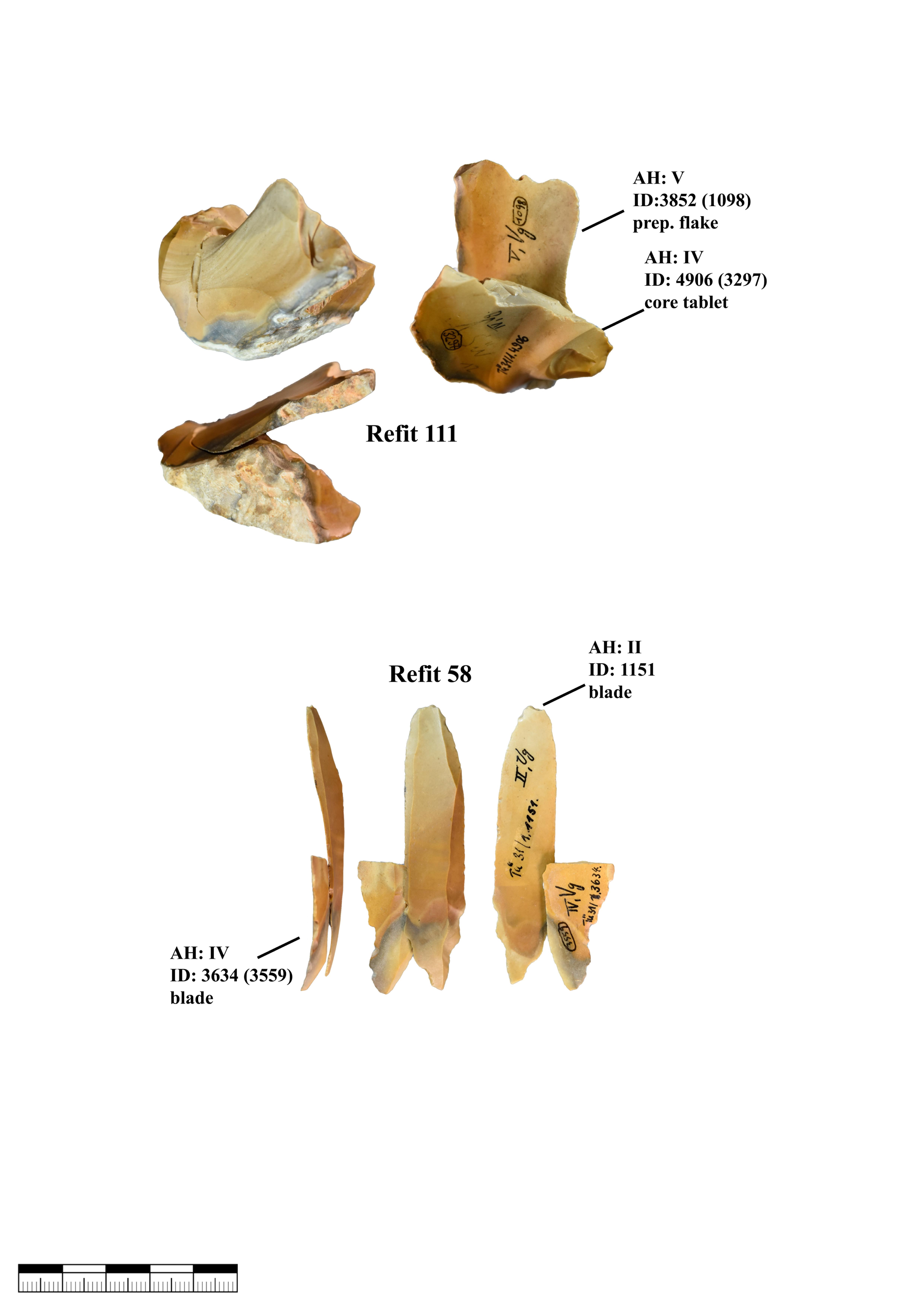

Supplement: S17 Fig — (TIF) [file pone.0331921.s021.tif]

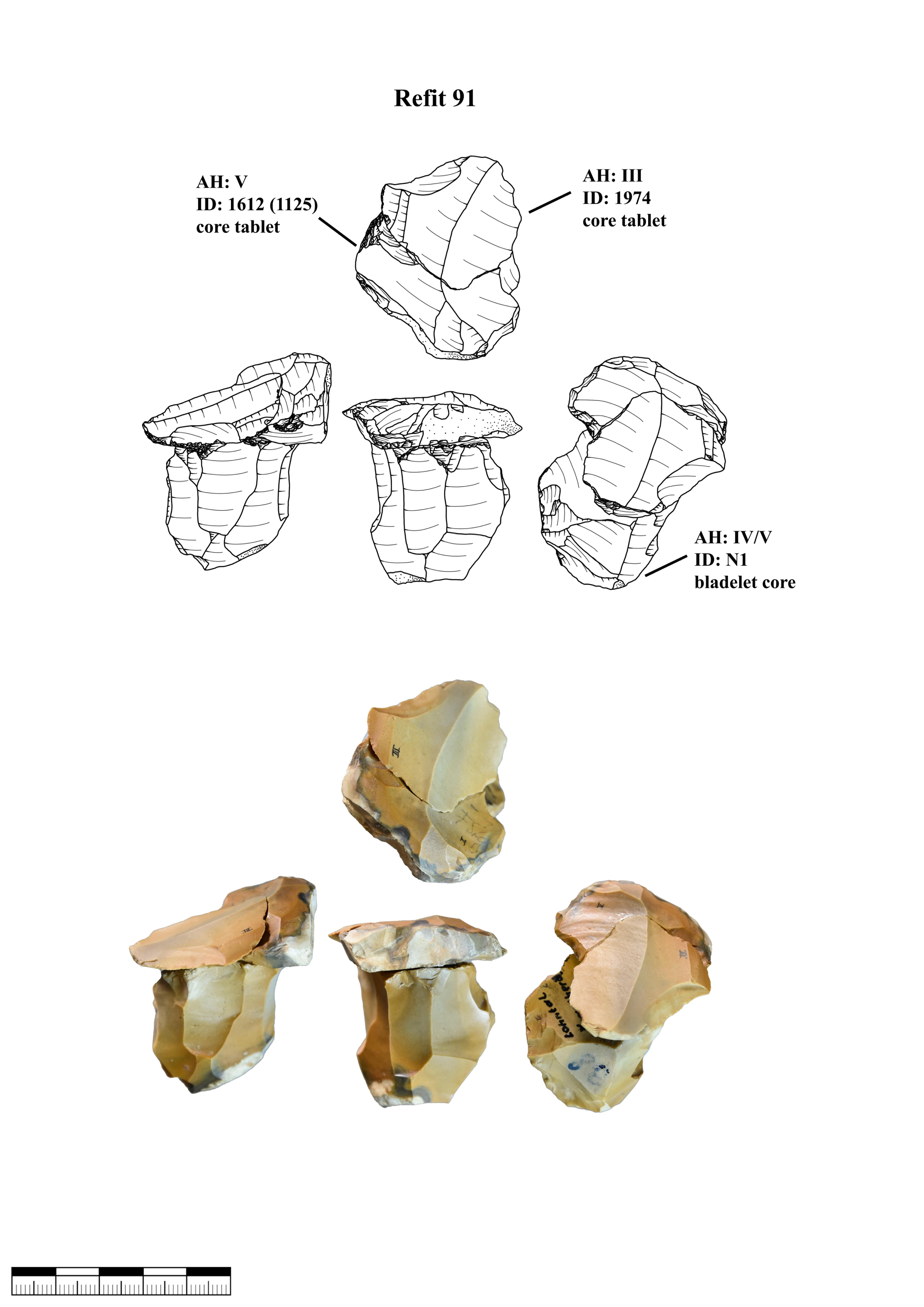

Supplement: S18 Fig — (TIF) [file pone.0331921.s022.tif]
